# Tandem UGT71B5s Catalyze Lignan Glycosylation in *Isatis indigotica* With Substrates Promiscuity

**DOI:** 10.3389/fpls.2021.637695

**Published:** 2021-03-31

**Authors:** Xiao Chen, Junfeng Chen, Jingxian Feng, Yun Wang, Shunuo Li, Ying Xiao, Yong Diao, Lei Zhang, Wansheng Chen

**Affiliations:** ^1^Center of Chinese Traditional Medicine Resources and Biotechnology, Institute of Chinese Materia Medica, Shanghai University of Traditional Chinese Medicine, Shanghai, China; ^2^School of Biomedical Sciences, Huaqiao University, Fujian, China; ^3^Biomedical Innovation R&D Center, School of Medicine, Shanghai University, Shanghai, China; ^4^Department of Pharmaceutical Botany, School of Pharmacy, Second Military Medical University, Shanghai, China

**Keywords:** Lignan glucosides, uridine diphosphate glycosyltransferase, *Isatis indigotica* Fort, recombinant enzyme catalysis, diversity

## Abstract

Lignans are a class of chemicals formed by the combination of two molecules of phenylpropanoids with promising nutritional and pharmacological activities. Lignans glucosides, which are converted from aglycones catalyzed by uridine diphosphate (UDP) glycosyltransferases (UGTs), have abundant bioactivities. In the present study, two UGTs from *Isatis indigotica* Fort., namely *Ii*UGT71B5a and *Ii*UGT71B5b, were characterized to catalyze the glycosylation of lignans with promiscuities toward various sugar acceptors and sugar donors, and pinoresinol was the preferred substrate. *Ii*UGT71B5a was capable of efficiently producing both pinoresinol monoglycoside and diglycoside. However, *Ii*UGT71B5b only produced monoglycoside, and exhibited considerably lower activity than *Ii*UGT71B5a. Substrate screening indicated that ditetrahydrofuran is the essential structural characteristic for sugar acceptors. The transcription of *IiUGT71B5s* was highly consistent with the spatial distribution of pinoresinol glucosides, suggesting that *Ii*UGT71B5s may play biological roles in the modification of pinoresinol in *I. indigotica* roots. This study not only provides insights into lignan biosynthesis, but also elucidates the functional diversity of the UGT family.

## Introduction

**Figure 1:**
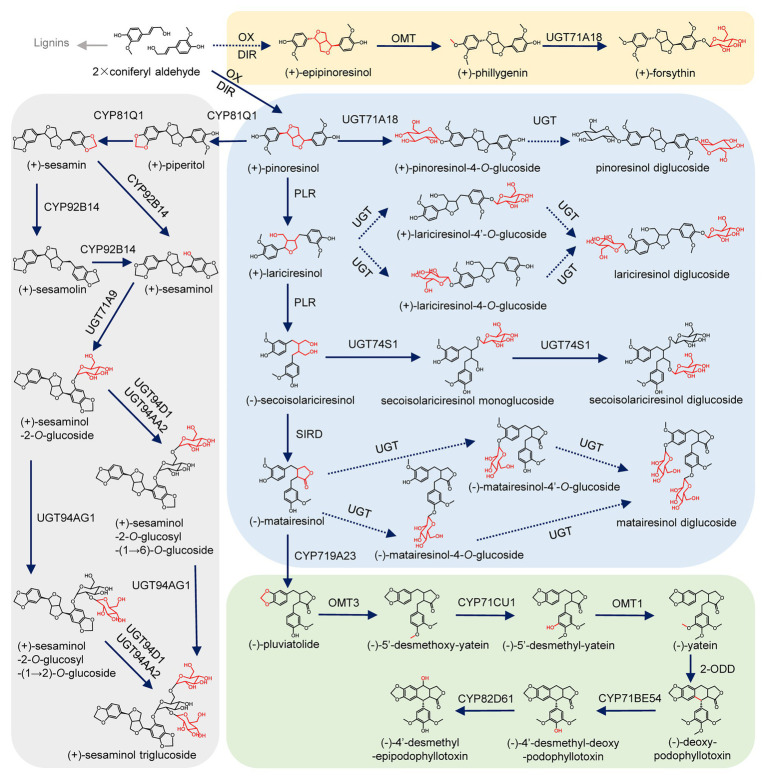
Biosynthetic pathway of lignans. Oxidation (OX), dirigent protein (DIR), cytochrome P450 (CYP), pinoresinol/lariciresinol reductase (PLR), secoisolariciresinol dehydrogenase (SIRD), O-methyltransferase (OMT), O-methyltransferase (OMT), 2-oxoglutarate/Fe(II)-dependent dioxygenase (2-ODD), piperitol/sesamin synthase (PSS), and uridine diphosphate-dependent glycosyltransferases (UGT). Uncharacterized steps are indicated by dashed lines.

Lignans, with a wide variety of clinically and dietarily important biological activities (Milder et al., 2005; Saarinen et al., 2007; Wang et al., 2011; Shi et al., 2012; Satake et al., 2013; Nantarat et al., 2020), are a class of derivatives formed by the combination of two molecules of phenylpropanoids (Fang and Hu, 2018). Lignans can be classified into eight subclasses depending on the way in which oxygen is incorporated into the skeleton and the cyclization pattern (Supplementary Figure S1; Teponno et al., 2016; Fang and Hu, 2018). The biosynthesis of lignans has been well-studied in *Isatis indigotica* Fort., *Linum usitatissimum* L., *Sinopodophyllum hexandrum (Royle)* Ying., *Sesamum indicum* Linn., *Forsythia koreana*, *Arabidopsis thaliana*, and other plants (Ono et al., 2010; Ghose et al., 2014; Lau and Sattely, 2015; Okazawa et al., 2015; Xiao et al., 2015; Teponno et al., 2016; Murata et al., 2017). As shown in Figure 1, two molecules of coniferyl alcohol undergo an oxidative coupling reaction to generate pinoresinol with the participation of dirigent protein (DIR; Davin et al., 1997). Then pinoresinol can be reduced by pinoresinol/lariciresinol reductase (PLR) to lariciresinol, which can be subsequently reduced to secoisolariciresinol under the catalysis of PLR. Next, the oxidative dehydrogenation of isolariciresinol produces matairesinol under the catalysis of secoisolariciresinol dehydrogenase (SIRD). The conversion from coniferyl alcohol to matairesinol represents the general biosynthetic pathway of plant lignans (Ghose et al., 2014; Xiao et al., 2015; Teponno et al., 2016). Matairesinol can be continuely converted into (−)-deoxypodophyllotoxin by cytochrome P450s (CYPs), *O*-methyltransferases (OMTs), and 2-oxoglutarate/Fe(II)-dependent dioxygenase (2-ODD), and finally hydroxylated by CYPs to produce (−)-4'-desmethyl-epipodophyllotoxin in *S. hexandrum* (Lau and Sattely, 2015). In another lignan biosynthesis pathway that mainly occurs in the seeds of *S. indicum*, pinoresinol can be catalyzed by CYP81Q1 to sequentially generate piperitol and sesamin, then sesamin can be converted into sesamolin and sesaminol (Murata et al., 2017; Ono et al., 2020). In *Forsythia suspensa*, after the transformation of coniferyl alcohol into epipinoresinol, phillygenin is produced by OMT (Ono et al., 2010). Ultimately, lignans are usually glycosylated by uridine diphosphate (UDP) glycosyltransferases (UGTs), and stored stably in plant cells (Lorenc-Kukula et al., 2005; Hano et al., 2006; Ono et al., 2010, 2020; Ghose et al., 2014; Teponno et al., 2016; Murata et al., 2017).

*Isatis indigotica* is a traditional Chinese medicinal herb, and its dried root (*Radix Isatidis*) is widely used for the treatment of influenza with lignan glucosides as the proven antiviral active ingredients (Lin et al., 2005; Yang et al., 2013; Li et al., 2015; Zhou et al., 2017; Runfeng et al., 2020). Currently, a variety of lignans and their glucosides have been identified from *I. indigotica*, including pinoresinol, lariciresinol, secoisolariciresinol, matairesinol, (+)-pinoresinol-4-*O*-glucoside, lariciresinol-*4*-*O*-glucoside, lariciresinol-4'-*O*-glucoside, pinoresinol diglucoside, lariciresinol diglucoside, etc. (Chen et al., 2013; Zhang et al., 2016, 2019). Each of these lignan glycoside exhibits both similar and specific bioactivities in mammals (Wang et al., 2011; Yang et al., 2013; Li et al., 2015, 2019; Zhou et al., 2017). Previous studies have elucidated the lignan biosynthetic pathway of *I. indigotica* from pinoresinol to secoisolariciresinol (Xiao et al., 2015). However, the catalytic enzymes involved in lignan glycosylation remain unknown.

Generally, glycosylation is catalyzed by UGT, which transfers a sugar moiety from a UDP-sugar to an acceptor molecule (Noguchi et al., 2008). To gain insights into lignan biosynthesis, we identified two lignan *UGT* genes, named *IiUGT71B5a* and *IiUGT71B5b*, which are responsible for glucosylation at the 4-position of pinoresinol. Meanwhile, the comprehensive catalytic properties and expression profiles of these two *Ii*UGTs were also characterized. Thus, our findings will be important for understanding the biosynthesis of lignans, as well as for elucidating the functional diversity of the UGT family.

## Materials and Methods

### Plant Materials

*Isatis indigotica* Fort. and *Nicotiana benthamiana*: the seeds were kept in our laboratory. The plants were growing under a constant temperature of 25°C, light for 16 h, and a constant temperature of 18°C in a dark environment for 8 h, with humidity of approximately 75%.

### Chemical Standards

The names and manufacturers of the standards are as follows: pinoresinol, (+)-pinoresinol-4-*O*-glucoside, lariciresinol, secoisolariciresinol, secoisolariciresinol monoglucoside, secoisolariciresinol diglucoside, isolariciresinol, (−)-isolariciresinol-9'-*O*-glucoside, matairesinol, matairesinoside, matairesinol monoglucoside, phillygenin, and forsythin (BioBioPha, China); pinoresinol diglucoside, clemaphenol A, trans-coniferin, coniferyl alcohol, sesaminol, quercetin, quercetin-3-*O*-glucoside, and quercetin-7-*O*-glucoside (SHYuanYe, China); quercetin-4'-*O*-glucoside (extracted by our laboratory), UDP-glucoside (UDP-Glc; Sigma, United States); UDP-xylose (UDP-Xyl), UDP-rhamnose (UDP-Rha), UDP-arabinose (UDP-Ara), and UDP-galacturonic acid (CarboSource, United States). All chemicals used in this study were of analytical or HPLC grade.

### UHPLC-Q-TOF/MS Based Metabolic Profiling

Lignans and phenylpropanoids profiling was carried out on an Agilent 1290A Infinity II ultra-performance liquid chromatography (UHPLC) system coupled with an Agilent 6530A accurate mass quadrupole-time of flight mass spectrometer (Q-TOF/MS; Agilent, United States) equipped with a dual AJS electrospray ionization source (ESI) operated in negative ion mode. The parameters were as follows: nitrogen drying gas temperature, 350°C; flow, 11 L∙min^−1^; nebulizer pressure, 45 psi; sheath gas temperature and flow rate were the same as those of the drying gas; capillary voltage, 4 kV; fragment voltage, 120 V; skimmer voltage, 60 V; octopole 1 RF peak voltage, 750 V; and mass range, 100–3,200 m/z. Chromatographic separations were performed using an Agilent Poroshell 120 SB-C18 column (2.7 μm, 2.1 mm × 150 mm; Agilent) at 35°C with the mobile phase consisting of (0.01% formic acid + 2 mM ammonium acetate) aqueous solution (phase A) and mass spectrometry grade acetonitrile (phase B), and the following elution method: 5% ACN at 0 min, 20% ACN at 2 min, 25% ACN at 10 min, 95% ACN at 20 min, and a final 4.5 min of equilibration post run. The injection volume was 3.0 μl, and the flow rate was 0.3 ml/min. The main mass spectrometry parameters of the target compound were all designated in the negative ion mode of UHPLC-Q-TOF/MS. Mass spectrometry parameters of the target compound in the negative ion mode of UHPLC-Q-TOF/MS are listed in Supplementary Table S1. All data acquisition and analysis were controlled by Agilent MassHunter Workstation Software (Agilent Technologies, United States).

### LC/MS Based Lignans and Phenylpropanoids Assay

The liquid phase mass spectrometer (LC/MS) was an Agilent 1200–6410 LC/MS, the chromatographic column was an Agilent ZORBAX SB-C18 (3.5 μm, 2.1 mm × 100 mm), the column temperature was 30°C, the flow rate was controlled at 0.3 ml/min, and the injection volume was 5 μl. The mobile phase was composed of acetonitrile (phase A) and 5 mM ammonium acetate aqueous solution (phase B), and the elution method was as follows: 14% ACN at 0 min, 50% ACN at 6 min, 85% ACN at 6.5 min, 85% ACN at 12 min, and a final 4.5 min of equilibration post run. The main mass spectrometry parameters of the target compound were all designated in the negative ion mode of LC/MS 6410 (Supplementary Table S2).

### Expression and Purification of UGTs

The coding regions of each *UGT* gene were subcloned into the pET-32a^+^ expression vector and then transformed into *Escherichia coli* strain BL21(DE3; primers are listed in Supplementary Table S3). The cell cultures were induced by 1 mM isopropyl-*β*-D-thiogalactoside (IPTG) until the OD_600_ reached 0.5–0.7. After 10–16 h of incubation at 16°C at 200 rpm, the cells were harvested by centrifugation at 4°C. The tagged recombinant proteins were purified by Ni-NTA affinity chromatography (Bio-Scale Mini Profinity IMAC Cartridges, BIO-RAD, United States).

### Activity Assays *in vitro*

Pinoresinol, (+)-pinoresinol-4-*O*-glucoside, lariciresinol, secoisolariciresinol, matairesinol, isolariciresinol, phillygenin, sesaminol, clemaphenol A, and coniferyl alcohol were selected as sugar acceptors. UDP-glucose, UDP-Xyl, UDP-Rha, UDP-Ara, and UDP-galacturonic acid were tested as sugar donors. The reaction was carried out in 50 μl of 100 mM phosphate buffer (pH 8.0), containing 2 mM sugar donor, 200 μM substrate, and 1 μg of purified protein. The reaction mixture without enzyme was preincubated at 30°C for 10 min, and then the purified protein was added and incubated at 30°C for 5 min to 12 h. The reaction was stopped by adding 150 μl of absolute ethanol. The reaction solution was evaporated to dryness, and reconstituted with methanol before chemical analysis.

### Sequence Analyses

Multiple sequence alignments of target UGTs were performed using the Clustal-W program, and phylogenetic trees were constructed using MEGA 7.0 (Kumar et al., 2016). The neighbor-joining statistical method was used to calculate the phylogenetic tree, with 1,000 bootstrap replications. The homology models of *Ii*UGT71B5a and *Ii*UGT71B5b were built using the crystal structure of *Medicago truncatula* UGT71G1 [Shao et al., 2005; Protein Data Bank (PDB) code: 2acv.1.A] as a template with the SWISS-MODEL server at http://swissmodel.expasy.org. UDP-glucose and sesaminol bound in GTB were taken as the sugar donor and sugar acceptor, respectively, and were docked into the built model of *Ii*UGT71B5a using Autodock 4.2. The models were visualized with the PyMOL molecular graphics system.^1^

### Transcription Analysis

Total RNA was extracted by a TransZol Plus RNA Kit (TransZol Up Plus RNA Kit, ER501, TransGen, China). cDNA was synthesized by one-step reverse transcription (PrimeScript™ 1st Strand cDNA Synthesis Kit, 6110A, TAKARA, China) using 2 μg of total RNA as a template. Gene expression levels were detected using real-time quantitative PCR [qRT-PCR; TB Green® Premix Ex Taq™ (Tli RNaseH Plus), RR420A, TAKARA, China; QuantStudio™ 3, Applied Biosystems, United States]. Gene specific primers are listed in Supplementary Table S3. Each group of samples had six biological replicates, and each biological replicate was assayed three times.

### Subcellular Localization of UGTs

The coding regions of *Ii*UGT71B5a and *Ii*UGT71B5b were cloned into the plant expression vector PHB-yellow fluorescent protein (YFP; Primers are listed in Supplementary Table S3). PHB-YFP vectors carrying *Ii*UGT71B5a and *Ii*UGT71B5b were transferred into *Agrobacterium tumefaciens* strain GV3101. Cultures were inoculated in 10 ml of YEB medium (containing 75 μg∙ml^−1^ kanamycin and 25 μg∙ml^−1^ rifampicin) overnight (28°C, 200 rpm) and collected by centrifugation (5,000 *g*, 10 min, RT). The collected cells were resuspended in Murashige and Skoog medium (10 mM MES, 100 μM acetosyringone, pH 5.8) to a final OD_600_ of 0.3–0.6. After incubation for 2–3 h at RT, the mixed *A. tumefaciens* was injected into the abaxial surface of leaves of 4-week-old *N. benthamiana* plantlets by needle-free syringes. The infected leaves were harvested 48–72 h after infiltration. *Agrobacterium tumefaciens* containing PHB-YFP was infiltrated as a negative control. The YFP fluorescence was imaged using a laser scanning confocal microscope (Leica TCS SP3, Germany).

### Synteny and Collinearity Analysis in Plant Genomes

Syntenic blocks were assigned *via* all-by-all BLASP with cutoffs of identity ≥40% and *e*-value ≤ 1e^−10^. Synteny comparison and Microsynteny visualization were performed using JCVI with LASTAL (Tang et al., 2008).

### Data Availability

The sequence data of IiUGT71B5a, IiUGT71B5b, and IiUGT71B5c have been submitted to the GenBank databases under accession numbers: MW051594, MW051595, and MW051596, respectively.

## Results

### Identification of UGTs

**Figure 2:**
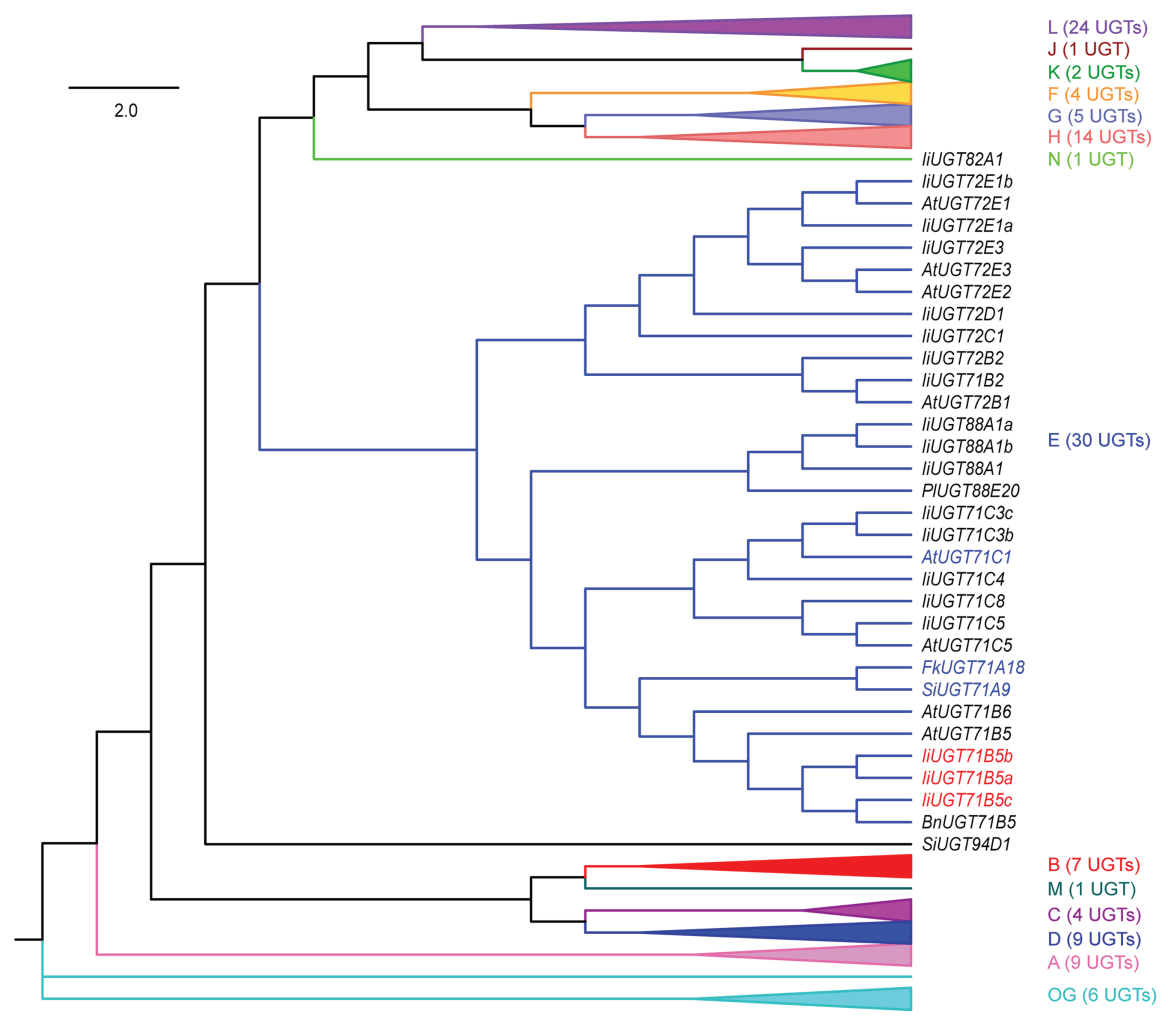
A neighbor-joining phylogenetic tree of 118 UGTs from selected plants. Groups A–N and the outgroup (OG) contain 83 *Ii*UGTs, *Fk*UGT71A18, *Bn*UGT71B5, *Lu*UGT74S1, *Si*UGT71A9, *Si*UGT94D1, *Pg*UGT84A24, *Pl*UGT88A20, and 28 *At*UGTs. For simplicity, only group E UGTs are shown, and the other 14 groups are folded. The expanded tree is shown in Supplementary Figure S2, and the protein sequences are listed in Supplementary Table S4. The neighbor-joining tree was constructed using MEGA 7.0 software with 1,000 bootstrap replicates. The tree is drawn to scale, with branch lengths measured in the relative numbers of substitutions per site. Blue color words indicate the UGTs that had been reported for pinoresinol glycosylation, red color words indicate the candidate *Ii*UGTs for pinoresinol glycosylation. *Ii*, *Isatis indigotica* Fort.; *Fk*, *Forsythia koreana*; *Lu*, *Linum usitatissimum* L.; *Si*, *Sesamum indicum* Linn.; *Bn*, *Brassica napus*; *Pg*, *Punica granatum*; *Pl*, *Pueraria lobata*; and *At*, *Arabidopsis thaliana*.

To annotate *UGT* genes from the *I. indigotica* genome (VHIU00000000; Kang et al., 2020), HMMER was used to search UGTs according to the plant secondary product glycosyltransferase (PSPG) motif (Yonekura-Sakakibara and Hanada, 2011; Caputi et al., 2012). As a result, 83 putative UGTs were identified, which were further assigned to 15 previously characterized groups based on phylogenetic tree construction (Figure 2; Wilson and Tian, 2019). Three *Ii*UGT71B5s (*Ii*UGT71B5a, *Ii*UGT71B5b, and *Ii*UGT71B5c) were suggested to have lignan catalytic activity as they have close phylogenetic relationship with a known pinoresinol glycosyltransferase (*Fk*UGT71A18) from *F. koreana* (Figure 2; Ono et al., 2010).

### Cloning and Functional Characterization of *Ii*UGT71B5a and *Ii*UGT71B5b

**Figure 3:**
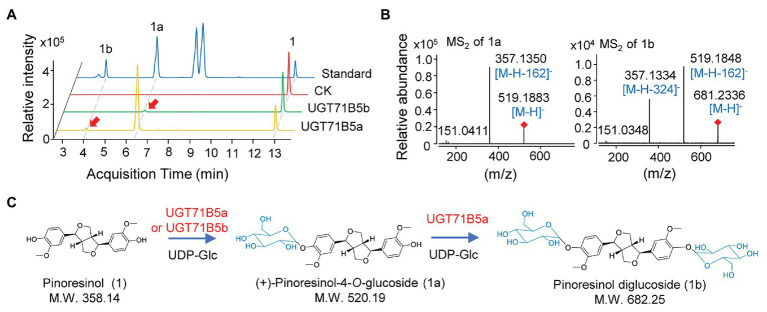
*O*-glucosylation of pinoresinol. **(A)** Ultra-performance liquid chromatography-quadrupole-time of flight mass spectrometer (UHPLC-Q-TOF/MS) chromatogram of the enzymatic reaction solution of recombinant UGT71B5a and UGT71B5b on pinoresinol and **(B)** the secondary mass spectra of 1a and 1b. The reaction was carried out in 50 μl of 100 mM phosphate buffer (pH 8.0), containing 2 mM uridine 5'-diphosphate glucoside (UDP-Glc), 200 μM pinoresinol, and 1 μg of purified protein, and then incubated at 30°C for 1 h. **(C)** The two-step glycosylation of pinoresinol (1), produced (+)-pinoresinol-4-*O*-glucoside (1a), and pinoresinol diglucoside (1b) in sequence.

*IiUGT71B5a* has an open reading frame (ORF) of 1,449 bp encoding 482 amino acids (aa), and *IiUGT71B5b* has an ORF of 1,443 bp encoding 480 aa. However, *IiUGT71B5c* only shows an ORF of 435 bp encoding a protein (145 aa) without the PSPG motif (Supplementary Figure S3). Thus, *Ii*UGT71B5a and *Ii*UGT71B5b were chosen for further studies. To identify the catalytic capability of the two *Ii*UGTs *in vitro*, recombinant *Ii*UGT71B5a and *Ii*UGT71B5b driven by pET-32a^+^ expression vectors were obtained using *E. coli* BL21(DE3) cells (Supplementary Figure S4), and pinoresinol (1) was tested as the sugar acceptor. With uridine 5'-diphosphate glucose (UDP-Glc) as the sugar donor, *Ii*UGT71B5a could efficiently convert pinoresinol (1) into 1a and 1b, while *Ii*UGT71B5b could only convert pinoresinol (1) into 1a (Figure 3A). The mass spectrum of 1a was identified with the [M-H]^−^ ion at m/z 519, which could produce fragments at m/z 357 ([M-H-162]^−^), indicating that 1a is a mono-*O*-glucoside (Figure 3B, 1a). The mass spectrum of 1b showed an [M-H]^−^ ion at m/z 682, which could produce fragments at m/z 519 ([M-H-162]^−^) and m/z 357 ([M-H-324]^−^), indicating that 1b is a di-*O*-glucoside (Figure 3B, 1b). Notably, 1a and 1b were detected with the same retention time as the authentic compounds of (+)-pinoresinol-4-*O*-glucoside and pinoresinol diglucoside, respectively (Figure 3A). The above results suggest that both *Ii*UGT71B5a and *Ii*UGT71B5b may be involved in lignan biosynthesis in *I. indigotica*, while only *Ii*UGT71B5a may contribute to the production of pinoresinol diglucoside (Figure 3C).

### Biochemical Properties of Recombinant *Ii*UGT71B5a

**Figure 4:**
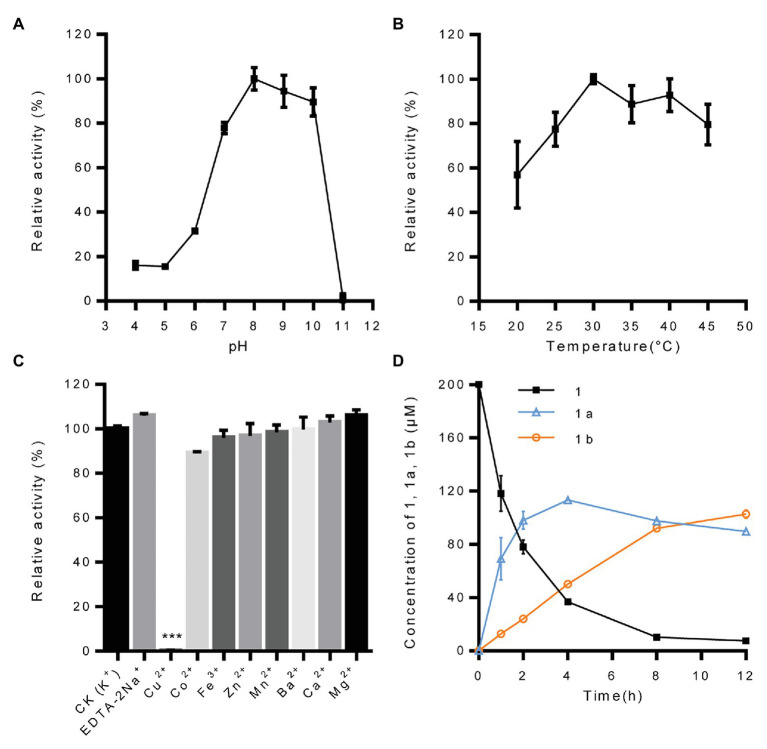
Biochemical properties of recombinant protein UGT71B5a. **(A)** Effect of pH on the enzyme activity of UGT71B5a. The relative activity of UGT71B5a at pH = 8.0 was set as 100%. **(B)** Effect of temperature on the enzyme activity of UGT71B5a. The relative activity of UGT71B5a at 30°C was set as 100%. **(C)** Effect of metal cations on the enzyme activity of UGT71B5a. The relative activity of UGT71B5a at 1 mM potassium chloride was set as 100%. **(D)** The concentration of pinoresinol (1), pinoresinol-4-*O*-glucoside (1a), and pinoresinol diglucoside (1b) changes with the reaction time (1–12 h). The changes of the three compounds (1, 1a, and 1b) in the enzyme reaction system within 60 min are shown in Supplementary Figure S10. The reaction was carried out in 50 μl of 100 mM phosphate buffer (pH 8.0), containing 2 mM UDP-Glc, 200 μM pinoresinol, 1 mM metal chlorine salt, and 1 μg of purified protein, and then incubated at 30°C for 1 h. When conducting a single factor (pH, temperature, metal cations, and reaction time) investigation, only the corresponding single variable is changed, and other variables remain unchanged. The ordinate value is expressed as the average value, and the error bar indicates SD (*n* = 3). ^***^p < 0.001.

With pinoresinol as the sugar acceptor and UDP-glucose as the sugar donor, the biochemical properties of *Ii*UGT71B5a were investigated. We found that *Ii*UGT71B5a exhibits its maximum activity at pH 8.0 and 30°C (Figures 4A,B). The effect of metal cations showed that *Ii*UGT71B5a is a non-cation-dependent protein, which is significantly inhibited by Cu^2+^ (Figure 4C). Under the catalysis of *Ii*UGT71B5a, pinoresinol (1) was first transferred into pinoresinol-4-*O*-glucoside (1a). As the reaction continued, pinoresinol-4-*O*-glucoside (1a) was gradually converted into pinoresinol diglucoside (1b). At 12 h after reaction, pinoresinol (1) was almost completely converted into pinoresinol-4-*O*-glucoside (1a) and pinoresinol diglucoside (1b; Figure 4D).

### Substrate Heterogeneities of *Ii*UGT71B5a and *Ii*UGT71B5b

**Figure 5:**
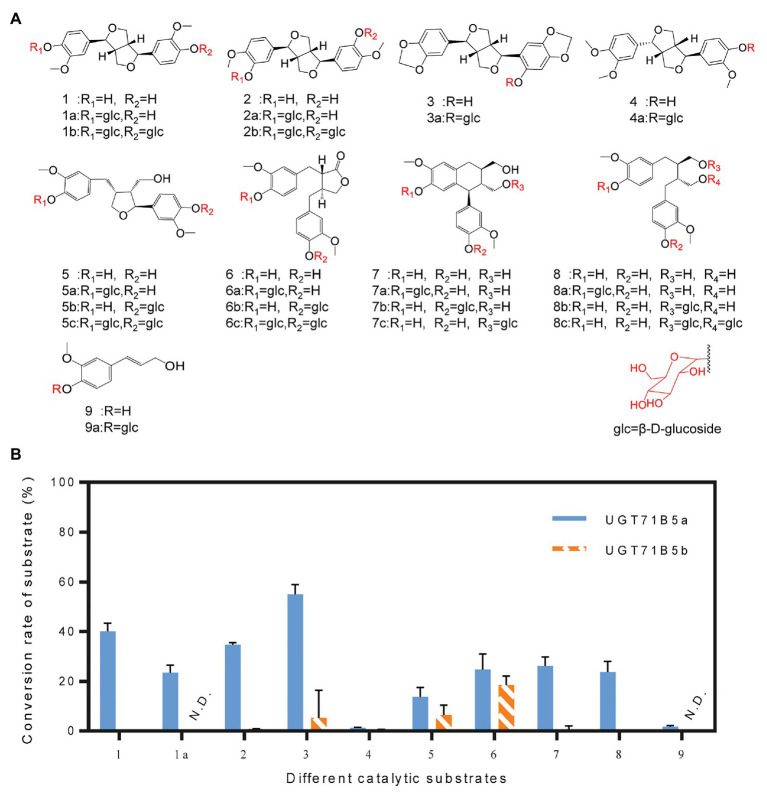
Substrate heterogeneities of UGT71B5a and UGT71B5b. **(A)** Structures of 1–9 and part of the glycosylated products (1: pinoresinol, 1a: (+)-pinoresinol-4-*O*-glucoside, 2: clemaphenol A, 3: sesaminol, 4: phillygenin, 5: lariciresinol, 6: matairesinol, 7: isolariciresinol, 8: secoisolariciresinol, and 9: coniferyl alcohol). **(B)** Conversion rates of glycosylated products for substrates 1–9, using UDP-Glc as the sugar donor. The reaction was carried out in 50 μl of 100 mM phosphate buffer (pH 8.0), containing 2 mM sugar donor, 200 μM substrate, and 1 μg of purified protein, and then incubated at 30°C for 1 h. The ordinate value is expressed as the average value, and the error bar indicates SD (*n* = 3).

It has been reported that UGTs have wide range of reorganization properties toward substrates (Koeller and Wong, 2001; Ono et al., 2010; Ban et al., 2012; Okazawa et al., 2015). To determine whether *Ii*UGT71B5a and *Ii*UGT71B5b are lignan glycosyltransferases with substrate promiscuity, a variety of lignans, including pinoresinol (1), (+)-pinoresinol-4-*O*-glucoside (1a), clemaphenol A (2), sesaminol (3), phillygenin (4), lariciresinol (5), matairesinol (6), isolariciresinol (7), and secoisolariciresinol (8), were tested as sugar acceptors (Figure 5A). Coniferyl alcohol (9), the common precursor of lignans, was also tested. The reactions were performed in 50 μl of 100 mM phosphate buffer (pH 8.0) containing 1 μg of purified protein, 2 mM UDP-Glc, and 200 μM sugar receptors. Using the UHPLC-Q-TOF/MS method, according to chemical standards (Supplementary Figure S5) or their MS^2^ fragments (Supplementary Figure S6), we found that *Ii*UGT71B5a could catalyze all tested substrates, and *Ii*UGT71B5b could catalyze eight of them (1–8), indicating that both *Ii*UGT71B5a and *Ii*UGT71B5b have high promiscuity. Moreover, *Ii*UGT71B5a had significantly higher catalytic activity toward each substrate compared to that of *Ii*UGT71B5b (Figure 5B). According to the characteristics of each glycosylated product, we proposed that *Ii*UGT71B5s have two catalytic properties. First, the conversional efficiency of *Ii*UGT71B5a varies depending on the substrate (Figure 5B). Compared to other types of lignans, *Ii*UGT71B5a has higher catalytic activities toward ditetrahydrofuran lignans (1, 1a, 2, and 3), including both backbones and their monoglucosides. Second, the glycosylation sites of *Ii*UGT71B5s are aromatic hydroxyl groups rather than aliphatic hydroxyl groups, supported by the productions of secoisolariciresinol and isolariciresinol (Supplementary Figure S5).

### Determination of Enzyme Kinetic Parameters

Considering that *Ii*UGT71B5b had much lower catalytic properties than *Ii*UGT71B5a, we focused on the kinetics of *Ii*UGT71B5a. The detailed catalytic properties of recombinant *Ii*UGT71B5a were measured using UDP-glucose as the sugar donor and a variety of lignan substrates as sugar acceptors. The kinetic parameters (Table 1) were analyzed through Lineweaver Burk plots (Supplementary Figure S7). As a result, *Ii*UGT71B5a had the highest catalytic efficiency for sesaminol (*kcat/Km* = 1.17 × 10^4^ s^−1^·M^−1^), followed by pinoresinol (*kcat/Km* = 1.04 × 10^3^ s^−1^·M^−1^), (+)-pinoresinol-4-*O*-glucoside (*kcat/Km* = 9.39 × 10^2^ s^−1^·M^−1^), clemaphenol A (*kcat/Km* = 7.16 × 10^2^ s^−1^·M^−1^), matairesinol (*kcat/Km* = 6.46 × 10^2^ s^−1^·M^−1^), and secoisolariciresinol (*kcat/Km* = 5.71 × 10^2^ s^−1^·M^−1^), with the lowest catalytic efficiency for lariciresinol (*kcat/Km* = 2.85 × 10^2^ s^−1^·M^−1^). In contrast, the catalytic efficiency of *Ii*UGT71B5b for sesaminol (*kcat/Km* = 1.06 × 10^3^ s^−1^·M^−1^) and matairesinol (*kcat/Km* = 4.51 × 10^2^ s^−1^·M^−1^) were relatively weak. According to the structures of these lignans, *Ii*UGT71B5a seemed to have a substrate preference for ditetrahydrofuran lignans. Given that sesaminol does not exist in *I. indigotica*, we proposed that *Ii*UGT71B5a is the major UGT contributing to the biosynthesis of pinoresinol glucosides.

**Table 1:** Enzyme kinetic parameters of recombinant UGT71B5a and UGT71B5b with lignans as substrates (20–200 μM) and uridine diphosphate (UDP)-glucose (2 mM) as a sugar donor.

UGT	Sugar receptor	*V_max_* (nmol·s^−1^)	*K_m_* (μM)	*k_cat_* (s^−1^)	*k_cat_*/*K_m_* (s^−1^·M^−1^)
UGT71B5a	Sesaminol	8.17 × 10^−3^	48.00 ± 0.79	5.63 × 10^−1^	1.17 × 10^4^
	Pinoresinol	1.02 × 10^−3^	67.16 ± 2.00	7.00 × 10^−2^	1.04 × 10^3^
	(+)-Pinoresinol-4-*O*-glucoside	1.85 × 10^−3^	136.06 ± 6.56	1.28 × 10^−1^	9.39 × 10^2^
	Clemaphenol A	1.28 × 10^−3^	123.20 ± 4.95	8.82 × 10^−2^	7.16 × 10^2^
	Matairesinol	5.84 × 10^−4^	62.35 ± 1.53	4.03 × 10^−2^	6.46 × 10^2^
	Secoisolariciresinol	1.39 × 10^−3^	168.48 ± 9.18	9.61 × 10^−2^	5.71 × 10^2^
	Lariciresinol	2.35 × 10^−3^	568.29 ± 16.08	1.62 × 10^−1^	2.85 × 10^2^
UGT71B5b	Sesaminol	2.08 × 10^−3^	135.48 ± 10.90	1.43 × 10^−1^	1.06 × 10^3^
	Matairesinol	4.01 × 10^−4^	61.34 ± 1.48	2.77 × 10^−2^	4.51 × 10^2^

The kinetic parameters were analyzed through Lineweaver Burk plots (Supplementary Figure S7). The detailed lignans and the reaction time of recombined UGT71B5s to different substrates were shown in Supplementary Table S6.

### Sugar Donor Preference of *Ii*UGT71B5a

**Figure 6:**
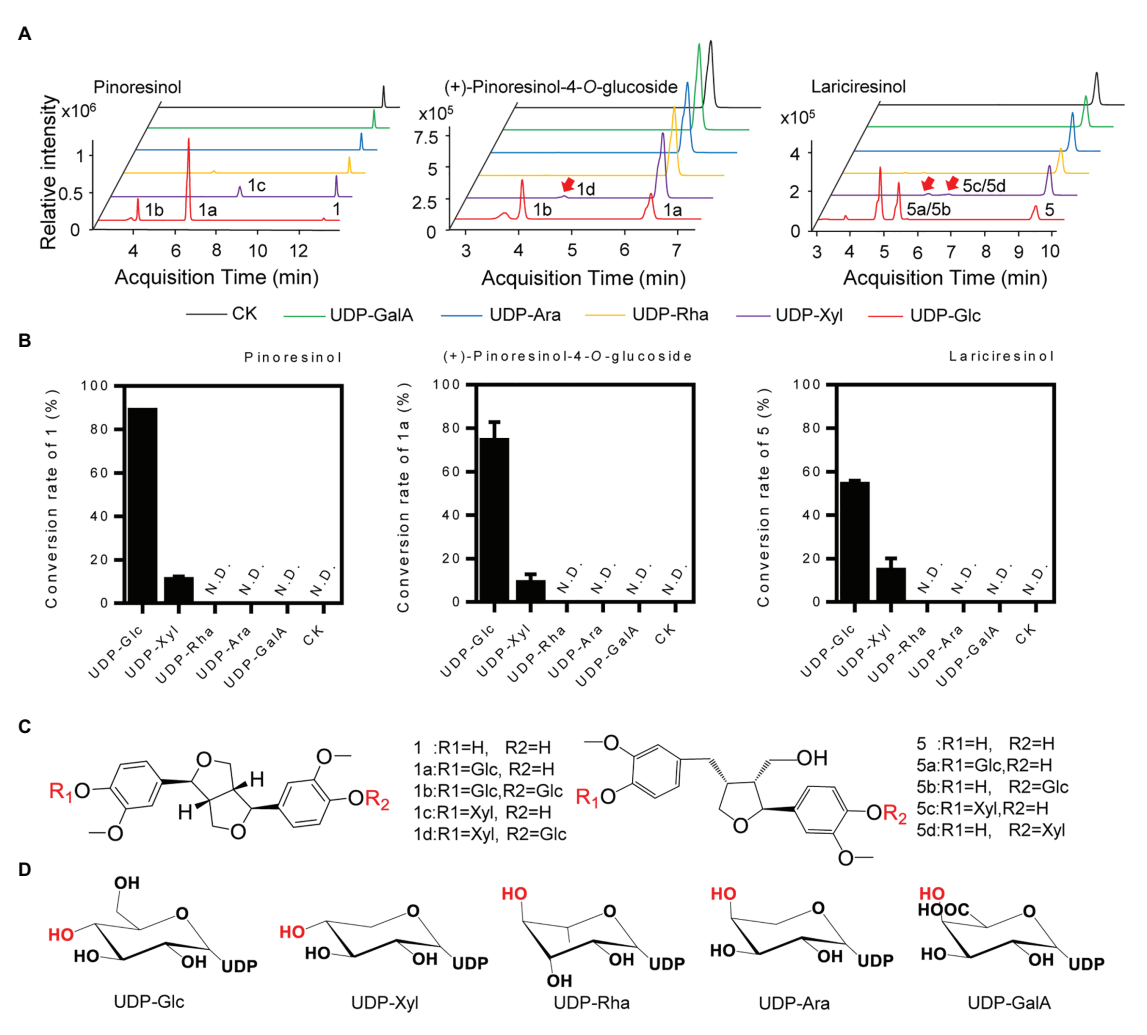
Sugar donor selectivity of UGT71B5a. **(A)** UHPLC-Q-TOF/MS chromatograms of purified recombinant UGT71B5a for the enzymatic reaction of three sugar receptors (1: pinoresinol, 1a: (+)-pinoresinol-4-*O*-glucoside, and 5: lariciresinol). **(B)** Conversion rates of substrates (1, 1a, and 5) catalyzed by recombinant UGT71B5a, using different sugar donors [UDP-Glc, UDP-xylose (UDP-Xyl), UDP-rhamnose (UDP-Rha), UDP-arabinose (UDP-Ara), and UDP-glucuronic acid (UDP-GalA)] separately. The reaction was carried out in 50 μl of 100 mM phosphate buffer (pH 8.0), containing 2 mM sugar donor, 200 μM substrate, and 1 μg of purified protein, and then incubated at 30°C for 12 h. The ordinate value is expressed as the average value, and the error bar indicates SD (*n* = 3). **(C)** The structures and glycosyl sites of sugar receptors. **(D)** The structures of sugar donors.

To determine the sugar donor specificity of *Ii*UGT71B5a, UDP-Glc, UDP-Xyl, UDP-Rha, UDP-Ara, and UDP-glucuronic acid (UDP-GalA) were tested (Figure 6A). When pinoresinol (1) was used as the substrate, UGT71B5a could efficiently utilize UDP-Glc (conversion rate of pinoresinol > 85%) and UDP-Xyl (conversion rate of pinoresinol > 10%), but not UDP-Rha, UDP-Ara, or UDP-GlcA (Figure 6B). In support of this, similar results were observed when using (+)-pinoresinol-4-*O*-glucoside (1a) or lariciresinol (5) as the substrate (Figure 6C). Consistent with previous report (Zhang et al., 2020), by comparing the structures of the five sugar donors (Figure 6D), we confirmed that the 4-OH configuration of the sugar group is an essential structural trait that affects the selective binding to the sugar donor. Furthermore, diglucosides (1b and 1d) with glucose and xylose moieties were also detected (Supplementary Figure S8).

### Modeling and Docking of *Ii*UGT71B5a and *Ii*UGT71B5b

**Figure 7:**
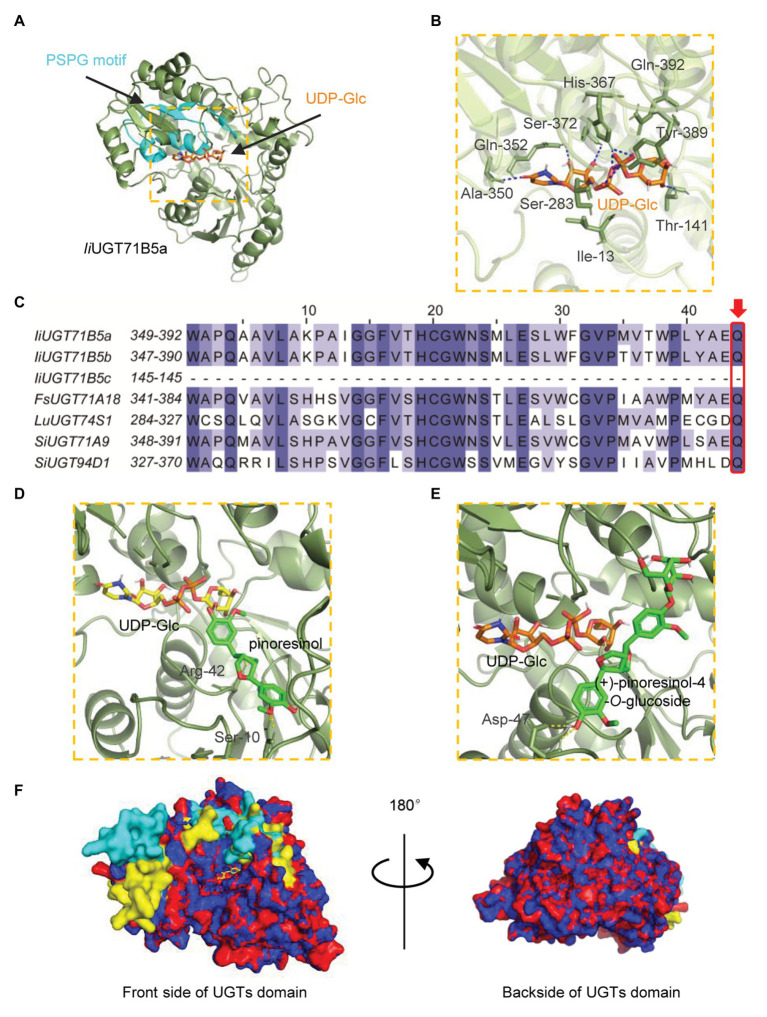
Homology modeling and multiple sequence alignment of *Ii*UGT71B5a and *Ii*UGT71B5b. **(A)** Cartoon representation of the structure of *Ii*UGT71B5a-UDP-glucose. UDP-glucose is shown as sticks. The structure of *Ii*UGT71B5a is shown as a dark green cartoon and the product glycosyltransferase (PSPG) motif is colored light blue. **(B)** Interactions between UDP-glucose and the residues of *Ii*UGT71B5a. Hydrogen bonds are represented by dark blue dashed lines. **(C)** The PSPG boxes of *Ii*UGT71B5a, *Ii*UGT71B5b, *Ii*UGT71B5c, *Fk*UGT71A18, *Lu*UGT74S1, *Si*UGT71A9, and *Si*UGT94D1. The key residues determining the UDP-sugar donor activity specificity are denoted with a red frame. **(D)** Binding mode of pinoresinol with *Ii*UGT71B5a-UDP-glucose predicted by molecular dynamics simulations. The structure is shown as a dark green cartoon, pinoresinol is shown as light green sticks, and UDP-glucose is shown as yellow sticks. Hydrogen bonds are represented by yellow dashed lines. **(E)** Binding model of (+)-pinoresinol-4-*O*-glucoside with *Ii*UGT71B5a-UDP-glucose predicted by molecular dynamics simulations. The structure is shown as a dark green cartoon, (+)-pinoresinol-4-*O*-glucoside is shown as light green sticks, and UDP-glucose is shown as orange sticks. Hydrogen bonds are represented by yellow dashed lines. **(F)** The divergent residues between *Ii*UGT71B5a and *Ii*UGT71B5b were mostly located near the entrance of the pocket. The overall structures of *Ii*UGT71B5a and *Ii*UGT71B5b are shown with an overlaid cartoon representation. Red and yellow represent *Ii*UGT71B5a. Blue and green represent *Ii*UGT71B5b. The different amino acid residues of the two *Ii*UGTs are shown in yellow and green, respectively.

To explore the potential structural basis for the catalytic properties of *Ii*UGT71B5a, molecular docking was performed to model the binding sites. A glycosyltransferase from *M. truncatula* was selected as the template (PBD: 2ACV; Shao et al., 2005). Binding sites for UDP-glucose were modeled first (Figure 7A). Several residues (Ile13, Thr141, Ser283, Ala350, Gln352, His367, Ser372, Tyr389, and Gln392) were shown to form hydrogen bonds (2.1–2.7 Å) with UDP-glucose, with a predicted affinity of −9.2 kcal·mol^−1^ (Figure 7B). Among these residues, most were located on the plant secondary product PSPG motif (Gln352, His367, Ser372, Tyr389, and Gln392; Figure 7C). Given that Gln392 is conserved and considered the critical residue for the preference toward UDP-glucose (Kubo et al., 2004), pinoresinol and pinoresinol-4-*O*-glucose were then docked into the predicted pocket (*Ii*UGT71B5a-UDP-Glc). Two amino acids (Ser10 and Arg42) were predicted to interact with pinoresinol, with a predicted affinity of −7.7 kcal·mol^−1^ (Figure 7D). Meanwhile, only one residue (Asp47) was predicted to form a hydrogen bond with pinoresinol-4-*O*-glucose, with a affinity of −6.9 kcal·mol^−1^ (Figure 7E). Notably, the two ligands approached UDP-glucose at different angles and positions is in the predicted pocket with an estimated volume of 1,121 Å^3^, which was a broad binding space. This might be caused by the structural basis for the substrate promiscuity of *Ii*UGT71B5s. Interestingly, when comparing the secondary structure of *Ii*UGT71B5s (90% sequence similarity), most of the different residues were located on the surface of the proteins and near the entrance of the pocket (Figure 7F), which might be critical for their difference in catalytic capability.

### Correlation Between Spatial Distribution of Lignans and Transcription of *IiUGT71B5s*

**Figure 8:**
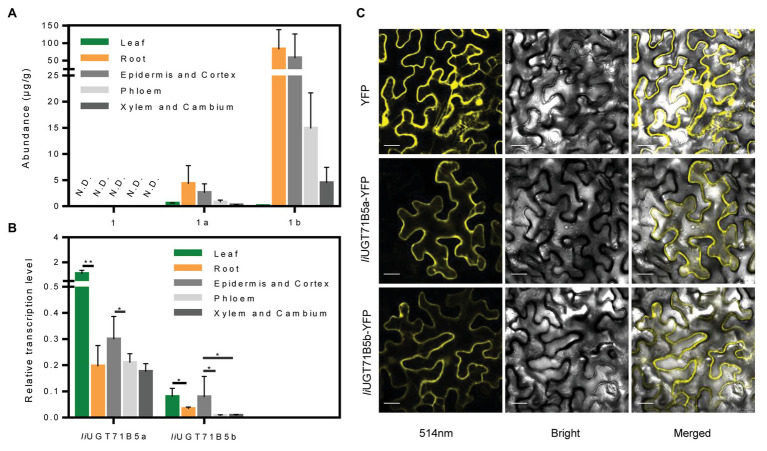
Expression characteristics of *Ii*UGTs and distribution characteristics of lignans. **(A)** The abundance of pinoresinol (1), (+)-pinoresinol-4-*O*-glucoside (1a), and pinoresinol diglucose (1b) in different organs (roots, leaves) and different root tissues (epidermis and cortex, phloem, xylem, and cambium) of *Isatis indigotica*. N.D. indicates not detected. **(B)** Relative gene expression levels of *IiUGT71B5a* and *IiUGT71B5b* in different organs (roots, leaves) and different root tissues (epidermis and cortex, phloem, xylem, and cambium) of *I. indigotica* (*n* = 6, ^*^p < 0.05, ^**^p < 0.01). **(C)** Subcellular localization of *Ii*UGT71B5a-yellow fluorescent protein (YFP) and *Ii*UGT71B5b-YFP in leaf cells of *Nicotiana benthamiana*. YFP exhibited yellow fluorescence under excitation light with a wavelength of 514 nm. Scale bars represent 20 μm.

To verify the correlation between *IiUGT71B5*s and correlated lignan glucosides, chemical profiling of lignans was carried out in different organs (leaf, root) and specific root cells (epidermis and cortex, phloem, xylem, and cambium). LC/MS profiling showed that at least three lignans and three lignan glucosides were present in *I. indigotica*, including pinoresinol, (+)-pinoresinol-4-*O*-glucoside, pinoresinol diglucose, lariciresinol, secoisolariciresinol, and secoisolariciresinol monoglucoside (Supplementary Table S5). Only a single lignan was analyzed and found that (+)-pinoresinol-4-*O*-glucoside and pinoresinol diglucose were mainly distributed in the roots, with the highest accumulation in the epidermis and cortex (Figure 8A). In contrast, pinoresinol was not detected in any root cells, which could be due to an extremely low accumulation. Besides, as a precursor of lignan biosynthesis, we also characterized coniferyl alcohol and found that coniferyl alcohol was not detected in these tissues (Supplementary Table S5). It is mainly stored in the form of trans-coniferin in plants because coniferyl alcohol may have a toxic effect on cells (Vaisanen et al., 2015). Interestingly, trans-coniferin has a high accumulation in roots, but it is almost undetectable in leaves, and the abundance of lignans in roots is much higher than that in leaves (Supplementary Table S5). These above results indicate that the process of synthesis and accumulation of lignans is mainly carried out in the roots, not in the leaves.

The correlated transcription level of *IiUGT71B5*s was analyzed by qRT-PCR. In different organs, the transcription levels of the two *IiUG71B5s* in leaves were much higher than those in roots. Among different root cells, the highest transcription levels of both *IiUGT71B5a* and *IiUGT71B5b* were detected in the epidermis and cortex cells, followed by phloem cells, and their expression in xylem and cambium cells were slightly lower than those in phloem (Figure 8B). The transcription of *IiUG71B5s* and the metabolism of pinoresinol glucosides are highly consistent in the roots of *I. indigotica*, suggesting that *IiUG71B5s* could possibly be a major contributor to the accumulation of pinoresinol glucosides in roots. However, the high expressions of *IiUG71B5s* did not increase the accumulation of pinoresinol glucosides in the leaves, which may be due to the extremely low biosynthesis of pinoresinol in leaves. Obviously, the low accumulation of pinoresinol is also caused by the low abundance of its precursor (trans-coniferin) in the leaves (Supplementary Table S5). In addition, the high transcription levels of *IiUG71B5s* in leaves imply that they may also assume wider glycosylation functions in plants, and the substrate heterogeneities of *Ii*UGT71B5s also support this conjecture (Figure 5).

### Subcellular Localizations of *Ii*UGT71B5a and *Ii*UGT71B5b

To investigate the subcellular localizations of *Ii*UGT71B5a and *Ii*UGT71B5b, their ORFs were fused with YFP at the *C*-terminus and transiently expressed in *N. benthamiana* leaves. As shown in Figure 8C, the signals of both fused proteins presented in the cytoplasm, which was consistent with those of previously reported UGTs, such as *Mt*UGT72L1, *Ph*UGT79B31, and *Pb*UGT72AJ2 (Pang et al., 2013; Knoch et al., 2018; Cheng et al., 2019). This result also indicated that the cytoplasm might be the subcellular site for lignan glycoside biosynthesis.

## Discussion

### Substrate Heterogeneities of *Ii*UGT71B5s

Although there are diverse types of lignans, only a few UGTs involved in lignan glycosylation have been discovered, including lignans (pinoresinol and lariciresinol) glycosyltransferase UGT71C1 from *A. thaliana*, pinoresinol glycosyltransferase UGT71A18 from *F. koreana*, secoisolariciresinol glycosyltransferase UGT74S1 from *L. usitatissimum*, and sesaminol glycosyltransferases UGT71A9, UGT94D1, UGT94AG1, and UGT94AA2 from *S. indicum* (Ono et al., 2010, 2020; Ghose et al., 2014; Okazawa et al., 2015; Teponno et al., 2016; Murata et al., 2017). UGTs, such as *At*UGT71C1, *Fk*UGT71A18, and *Ii*UGT71B5s are able to catalyze glycosylation of pinoresinol, however, their substrate catalytic characteristics are not the same. For example, *At*UGT71C1 has a more extensive substrate heterogeneity than *Ii*UGT71B5s, which can catalyze phenylpropanoids, flavonoids, as well as lignans (Lim et al., 2003; Okazawa et al., 2015). *Fk*UGT71A18 also exhibits relatively broad sugar acceptor specificity for lignans with a preference for ditetrahydrofuran lignans, but it performs well catalytic activity for phillygenin (Figure 5B, **compound 4**) compared with *Ii*UGT71B5s (Ono et al., 2010). The identification of novel lignan glucosyltransferases from *I. indigotica* based on the similarity to the *Fs*UGT71A18, *At*UGT71C1, and *Si*UGT71A9 highlights the structural conservation of lignan UGTs across plant species (Figure 2). Nevertheless, similar to flavonoid UGTs forming independent phylogenetic clades based on their various regio-specificities (Noguchi et al., 2009), the structural diversity of lignan glucosides strongly suggests that not all lignan UGTs belong to the UGT71 family, such as *Lu*UGT74S1, *Si*UGT94D1, *Si*UGT94AG1, and *Si*UGT94AA2 (Supplementary Figure S2). In addition to the catalytic activities of lignans, glycosylation 3-OH of quercetin by *At*UGT71B5 was also reported (Lim et al., 2004). *Ii*UGT71B5s, as homologous genes of *At*UGT71B5, are preferred to glycosylate 3'-OH of quercetin, indicating that functional differentiation occurs in UGT71B5s (Figure 2; Supplementary Figure S11).

Among these known UGTs, most have been validated to have functional plasticity with a wide range of substrate recognition toward a variety of lignans. In this study, the promiscuity of both sugar acceptors and donors of two *Ii*UGT71B5s was demonstrated. On the other hand, although *Ii*UGT71B5s are capable of catalyzing multiple lignan substrates (Figure 5), we surmise that two *Ii*UGT71B5s may contribute mainly to the catalysis of pinoresinol, owing to its substrate preference (Figure 5B) and high consistency with the accumulation of pinoresinol glucosides in the roots of *I. indigotica* (Figures 8A,B). Notably, the similar catalytic activities and transcription patterns suggested functional redundancy between *IiUGT71B5a* and *IiUGT71B5b* (Laruson et al., 2020). In support of this, a tandem duplication, including three loci (*Ii4G26670*, *Ii4G26680*, and *Ii4G26690*) coding *UGT71B5* genes was discovered on chromosome 4 (Supplementary Figure S12), which could be the primary source for redundancy of *UGT71B5* genes. In addition, *Ii4G26690* (*IiUGT71B5c*) represented as a partial gene with an ORF region of 435 bp, accompanied by two extra introns (2,009 and 104 bp; Supplementary Figures S3, S13), indicating the pseudogenization of this locus. Although transcription of *IiUGT71B5c* was detected in the leaves and roots of *I. indigotica* (Supplementary Figure S9), without the PSPG box it could only become a nonfunctional UGT (Supplementary Figure S3). However, it is also possible that the *in vivo* activity of *Ii*UGT71B5s does not match its *in vitro* activity, which has been reported in many plant UGT proteins, including *At*UGT73C6, *Mt*UGT78G1, and *Lj*UGTs (Peel et al., 2009; Husar et al., 2011; Yin et al., 2017). Therefore, further validation of the roles of UGT71B5s *in planta* is required. Interestingly, although sesaminol (Figure 5B, **compound 3**) and its glucosides are not produced in *I. indigotica*, *Ii*UGT71B5a had the strongest activity toward sesaminol, indicating that *Ii*UGT71B5a might be used as an efficient catalytic element for biosynthesis.

### Catalytic Properties of *Ii*UGT71B5s

*Ii*UGT71B5s seemed to exhibit similar functions as previously reported lignan UGTs such as *Fk*UGT71A18, with a wide range substrate promiscuity (Ono et al., 2010). However, specificities of substrate were also discovered in *Ii*UGT71B5s catalyzation. First, although *Ii*UGT71B5s catalyzed various lignan substrates, they have showed obvious substrate preferences toward lignans containing ditetrahydrofuran on the skeleton, such as pinoresinol (1), clemaphenol A (2), and sesaminol (3; Figure 5). In addition, among the tested four ditetrahydrofuran lignan substrates (Figure 5A, **compounds 1–4**), *Ii*UGT71B5s had extremely low activities toward phillygenin (4), the only substrate in the S configuration with a phenolic group at *C*-1, supporting the strong stereoselectivity of *Ii*UGT71B5s. Furthermore, conserved activities toward sugar donors were also observed. *Ii*UGT71B5s only showed high conversion efficiency with UDP-Glc, which was supposed to correlate with the conserved glutamine (Gln, Q) located at the end of the PSPG motif (Supplementary Figure S3; Kubo et al., 2004).

### Evolution of the Lignan Biosynthesis Pathway

Lignans represent eight categories according to the differences in their basic skeleton (Teponno et al., 2016; Fang and Hu, 2018), and they have diverse chemical compositions and distributions in the plant kingdom (Ono et al., 2010; Lau and Sattely, 2015; Murata et al., 2017; Zhang et al., 2019). Thus, the diversity of lignan biosynthesis pathways in different plants provides an ideal example to study the evolution of the origins and the loss of plant chemical diversity. For example, the major lignans accumulated in the roots of *A. thaliana* are lariciresinol glucosides but not secoisolariciresinol, which is determined by variance in the activity of PLR (Nakatsubo et al., 2008). In this study, we found that the pinoresinol in *I. indigotica* is not only catalyzed by PLR to produce lariciresinol and secoisolariciresinol (Xiao et al., 2015; Zhang et al., 2016), but also accumulates in the form of its glucosides (Figure 1; Supplementary Table S5), which may be influenced by functional differentiation of UGT71B5s. In addition, some categories of lignans are not produced in *I. indigotica*, such as sesaminol and podophyllotoxin, which is due to the functional diversity of gene families involved in the lignan biosynthesis network, including CYPs (Murata et al., 2017; Harada et al., 2020), OMTs (Lau and Sattely, 2015), and UGTs (Ono et al., 2020). Studying the activity, diversity, and evolution of these families will help to reveal mechanisms for the diversity of lignans in plants.

In summary, we identified two UGTs that may primarily contribute to the modification of pinoresinol. We discussed the structural insights of their functional diversity, which will provide an in-depth understanding of lignan biosynthesis and the functional diversity of the UGT family in plants. In addition, these novel UGTs may facilitate further enzyme engineering to produce bioactive lignan glucosides.

## Data Availability Statement

The datasets presented in this study can be found in online repositories. The names of the repository/repositories and accession number(s) can be found in the article/Supplementary Material.

## Author Contributions

WC, LZ, and JC were the leading investigators of this research program. JC and XC designed the experiments and wrote the manuscript. XC performed the most of experiments and analyzed the data. JF performed the molecular docking. YW, SL, YX, and YD assisted in experiments and discussed the results. All authors contributed to the article and approved the submitted version.

### Conflict of Interest

The authors declare that the research was conducted in the absence of any commercial or financial relationships that could be construed as a potential conflict of interest.

## Footnotes

^1^http://www.pymol.org
