# Supplementary material for: Tandem UGT71B5s Catalyze Lignan Glycosylation in Isatis indigotica With Substrates Promiscuity
Source: Front Plant Sci. 2021 Mar 31;12:637695. doi: 10.3389/fpls.2021.637695 (PMC8044456; doi:10.3389/fpls.2021.637695)
Supplement: Supplementary file 1 [file Data_Sheet_1.docx]

Supplementary Material

Tandem UGT71B5s Catalyze Lignan Glycosylation in *Isatis indigotica* with Substrates Promiscuity

Xiao Chen^1,2^†, Junfeng Chen^1^†, Jingxian Feng^1^†, Yun Wang^4^, Shunuo Li^1^, Ying Xiao^1^, Yong Diao^2^, Lei Zhang^3^* and Wansheng Chen^1^*

^1^ Center of Chinese Traditional Medicine Resources and Biotechnology, Institute of Chinese Materia Medica, Shanghai University of Traditional Chinese Medicine, Shanghai, China

^2^ School of Biomedical Sciences, Huaqiao University, Fujian, China

^3^ Department of Pharmaceutical Botany, School of Pharmacy, Second Military Medical University, Shanghai, China

^4^ Biomedical Innovation R&D Center, School of Medicine, Shanghai University, Shanghai, China

† These authors have contributed equally to this work.

*** Correspondence:**Wansheng Chen
chenwansheng@shtcm.edu.cn

Lei Zhang
zhanglei@smmu.edu.cn

# Supplementary Figures and Tables

## Supplementary Figures


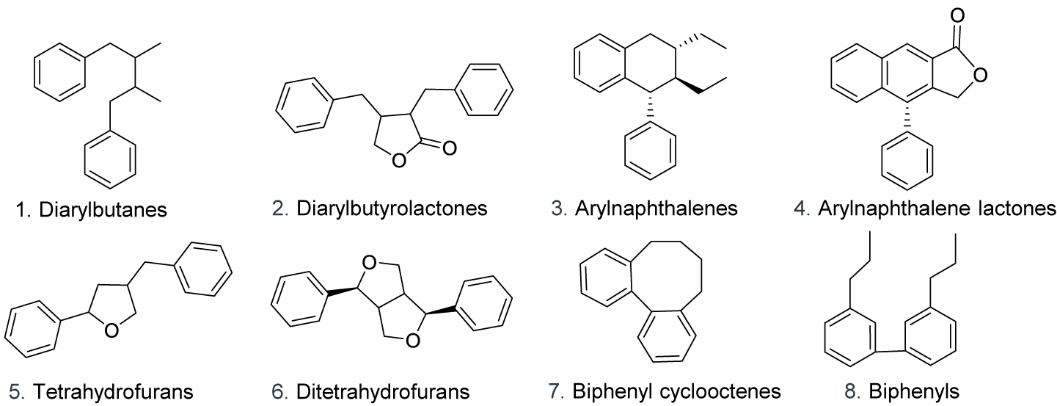


**Supplementary Figure 1.** The chemical structural formulas of eight lignan compounds.


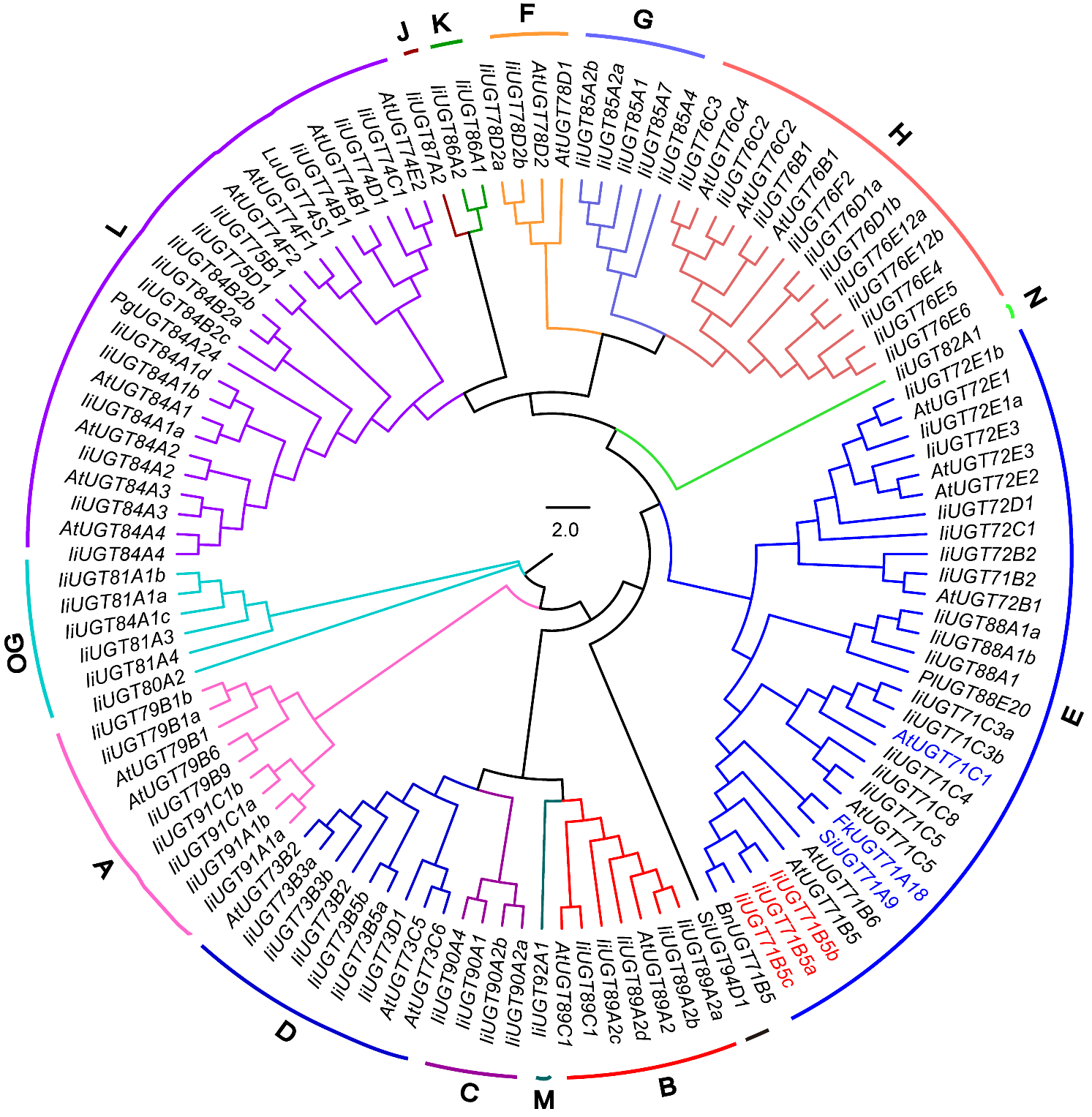


**Supplementary Figure 2.** A neighbor-joining phylogenetic tree of 118 UGTs from selected plants.

Groups A–N and the outgroup (OG) contain 83 *Ii*UGTs, *Fk*UGT71A18, *Bn*UGT71B5, *Lu*UGT74S1, *Si*UGT71A9, *Si*UGT94D1, *Pg*UGT84A24, *Pl*UGT88A20 and 28 *At*UGTs. The protein sequences were listed in **Supplementary Table 4**. The neighbor-joining tree was constructed using MEGA 7.0 software with 1000 bootstrap replicates. The tree is drawn to scale, with branch lengths measured in the relative numbers of substitutions per site. Blue color words indicate the UGTs that had been reported for pinoresinol glycosylation, red color words indicate the candidate *Ii*UGTs for pinoresinol glycosylation. *Ii*, *Isatis indigotica* Fort.; *Fk*, *Forsythia koreana*; *Lu*, *Linum usitatissimum* L.; *Si*, *Sesamum indicum* Linn.; *Bn*, *Brassica napus*; *Pg*, *Punica granatum*; *Pl*, *Pueraria lobata*; *At*, *Arabidopsis thaliana*.


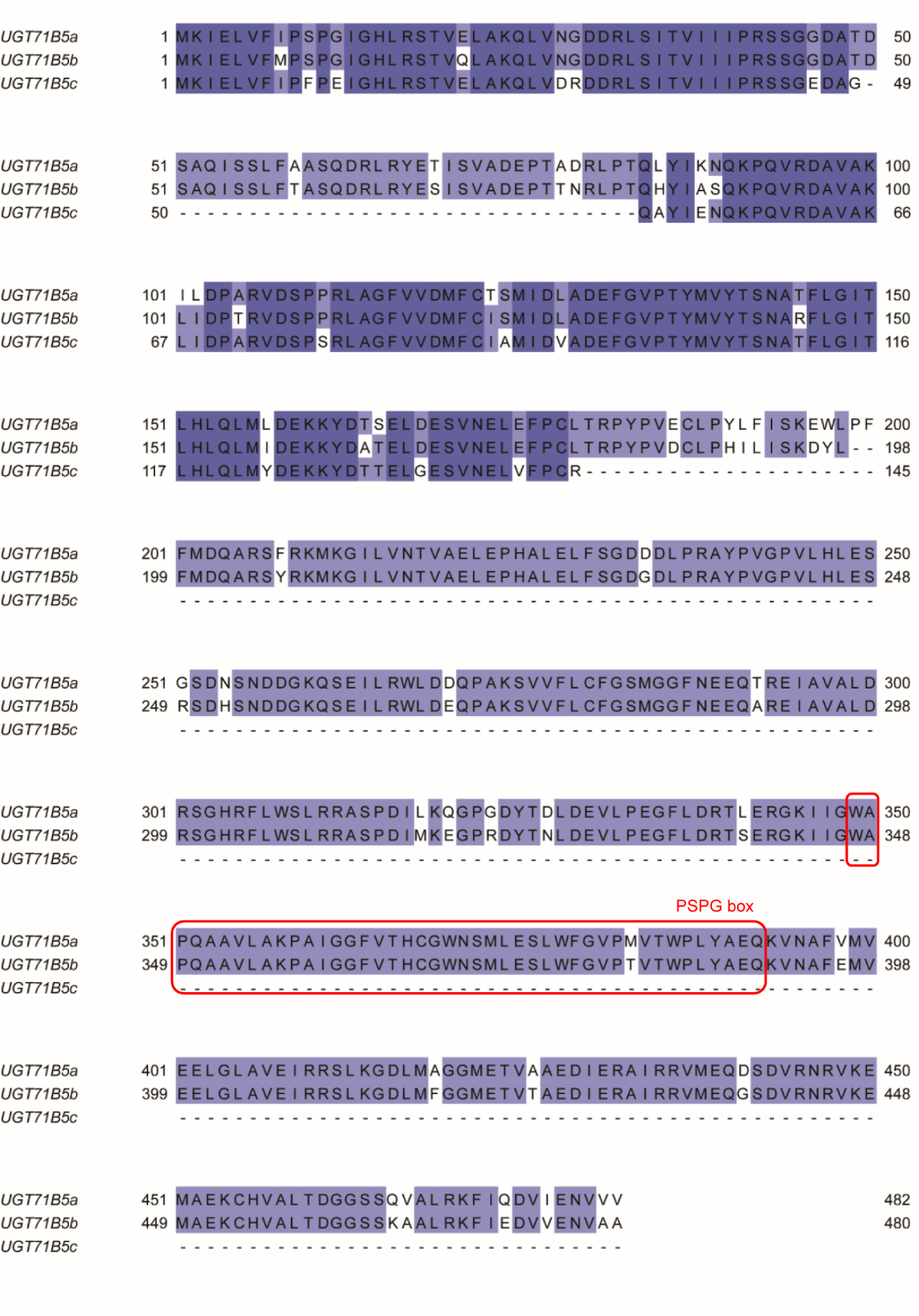


**Supplementary Figure 3.** Multiple sequence alignment of *Ii*UGT71B5a, *Ii*UGT71B5b and *Ii*UGT71B5.

Multiple sequence alignment was performed using the Clustal-W program (MEGA 7.0). The PSPG box of these aligned sequences is indicated by a red frame.


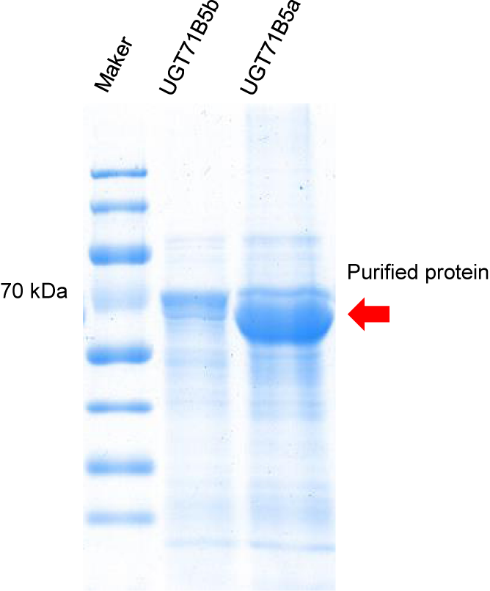


**Supplementary Figure 4.** SDS-PAGE analysis of purified proteins.

SDS-PAGE showed purified *Ii*UGT71B5a (53.8 kDa) and *Ii*UGT71B5b (53.5 kDa) fused with a His-tag (18 kDa).


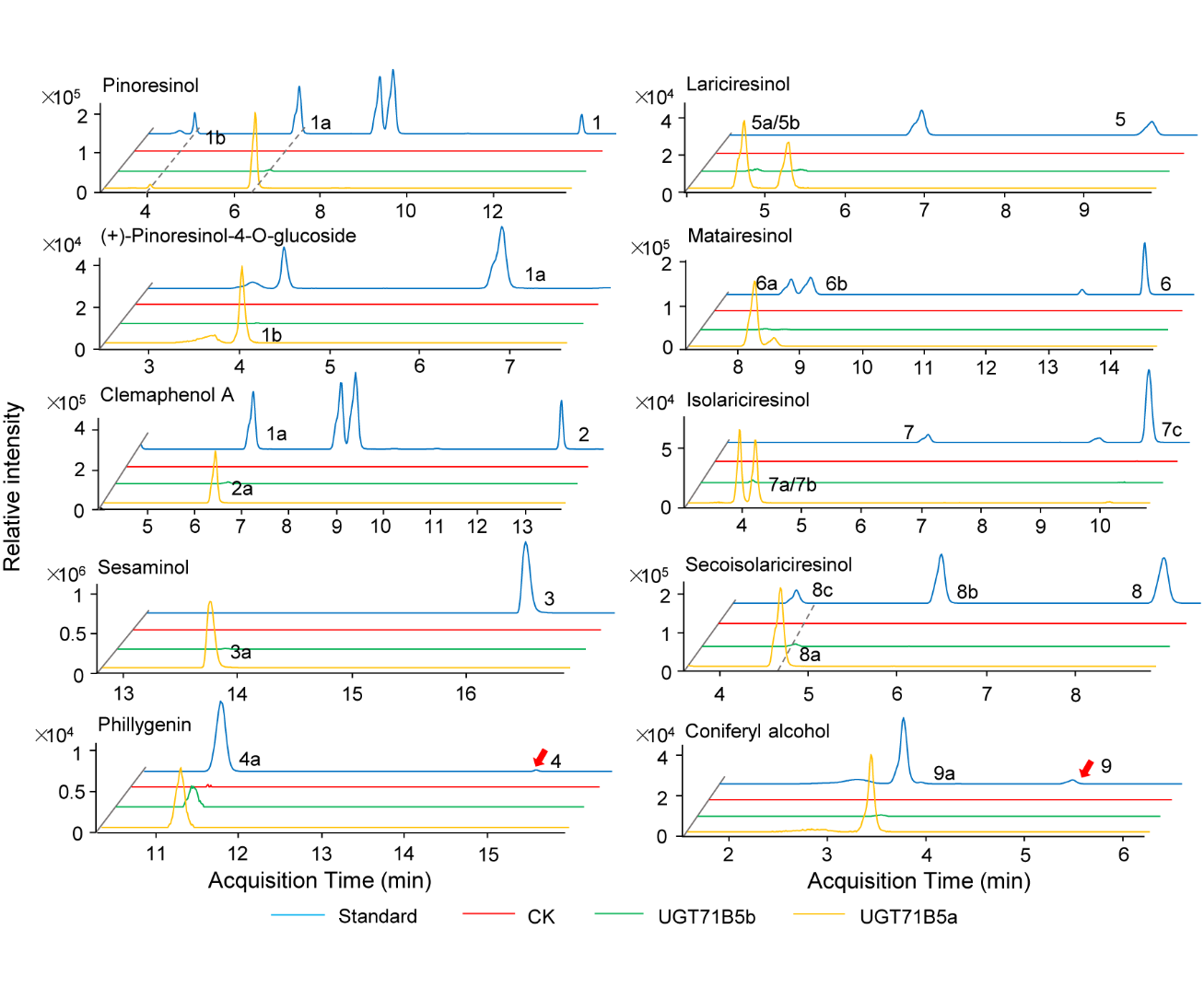


**Supplementary Figure 5.** UHPLC-Q-TOF/MS chromatogram of *Ii*UGT71B5a and *Ii*UGT71B5b enzymatic reactions on different substrates.

The annotation of each chemical is consistent with Figure 5.


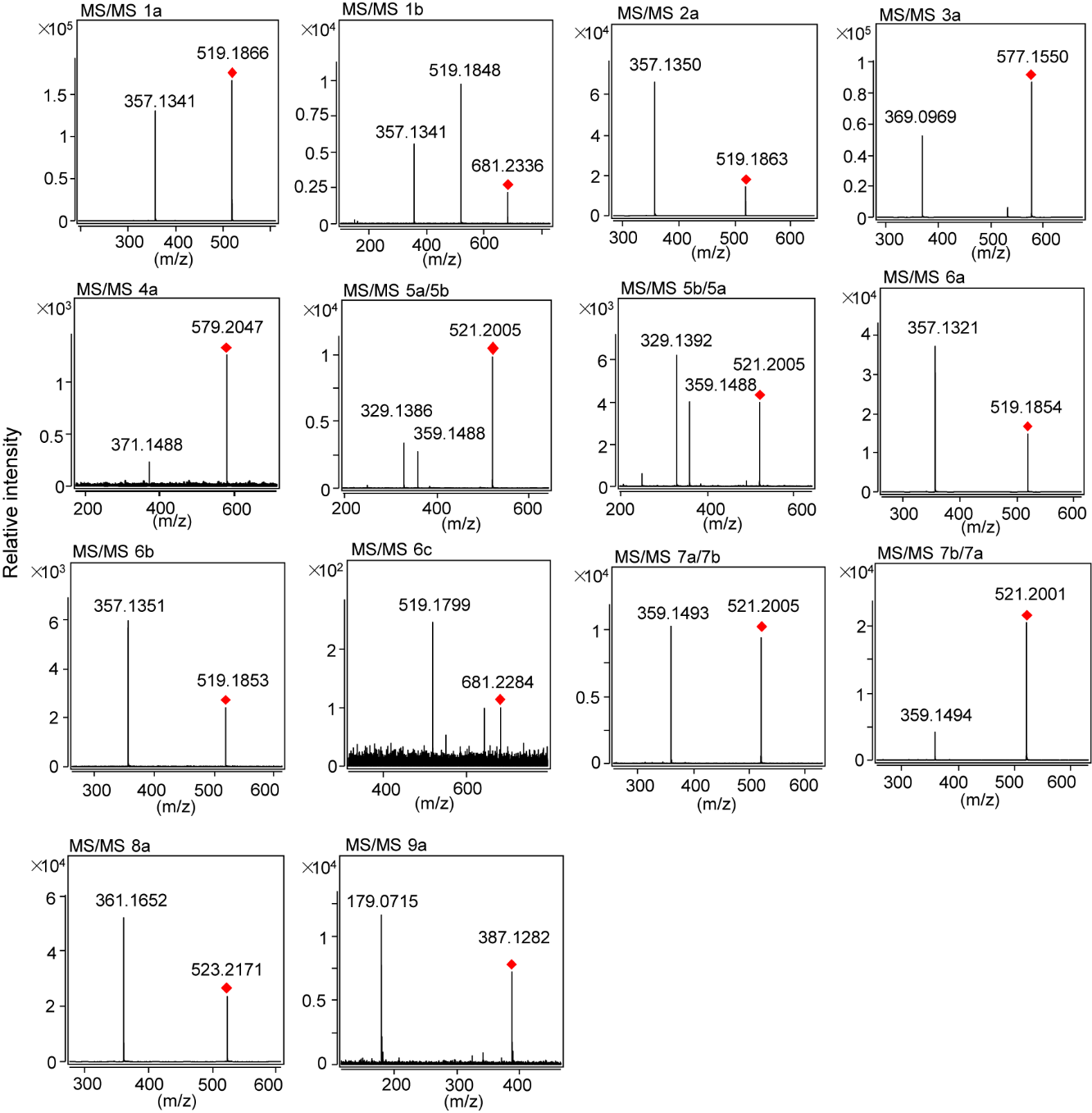


**Supplementary Figure 6.** The secondary mass spectra of the substrates and products in the enzymatic reaction of the recombinases *Ii*UGT71B5a and *Ii*UGT71B5b on different substrates in UHPLC-Q-TOF/MS negative ion mode.

The annotation of each chemical is consistent with Figure 5.


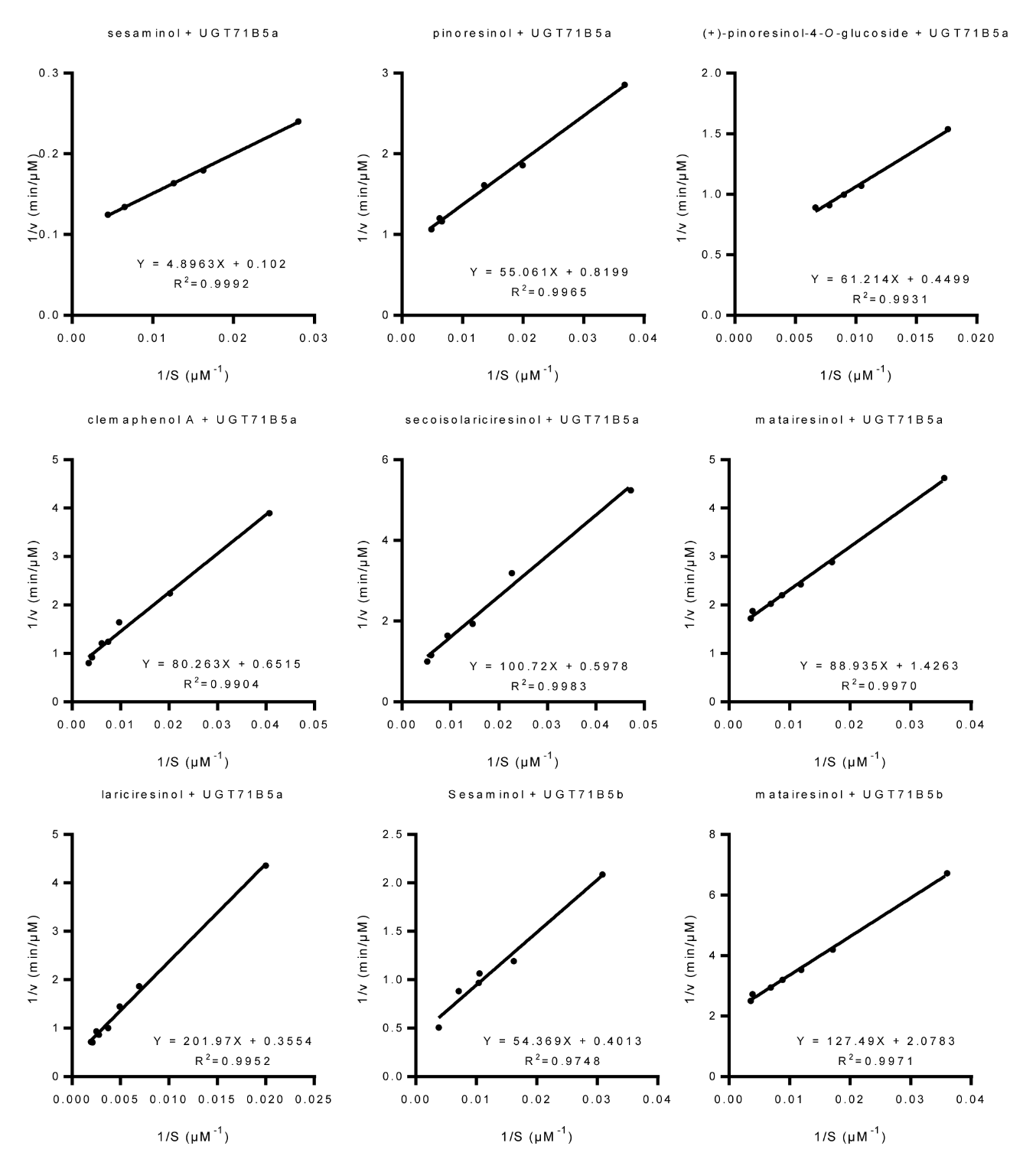


**Supplementary Figure 7.** The double reciprocal equations of substrate concentration (S) and the initial rate of enzyme catalysis (v).

The reaction was carried out in 50 μL of 100 mM phosphate buffer (pH 8.0) containing 2 mM UDP-glucose, 20-200 μM substrate, and 1 μg of purified protein. The reaction mixture without enzyme was preincubated at 30 ℃ for 10 min, and then the purified protein was added. Notes: The detailed lignans and the reaction time of recombined UGT71B5s to different substrates were shown in Supplementary Table 6.


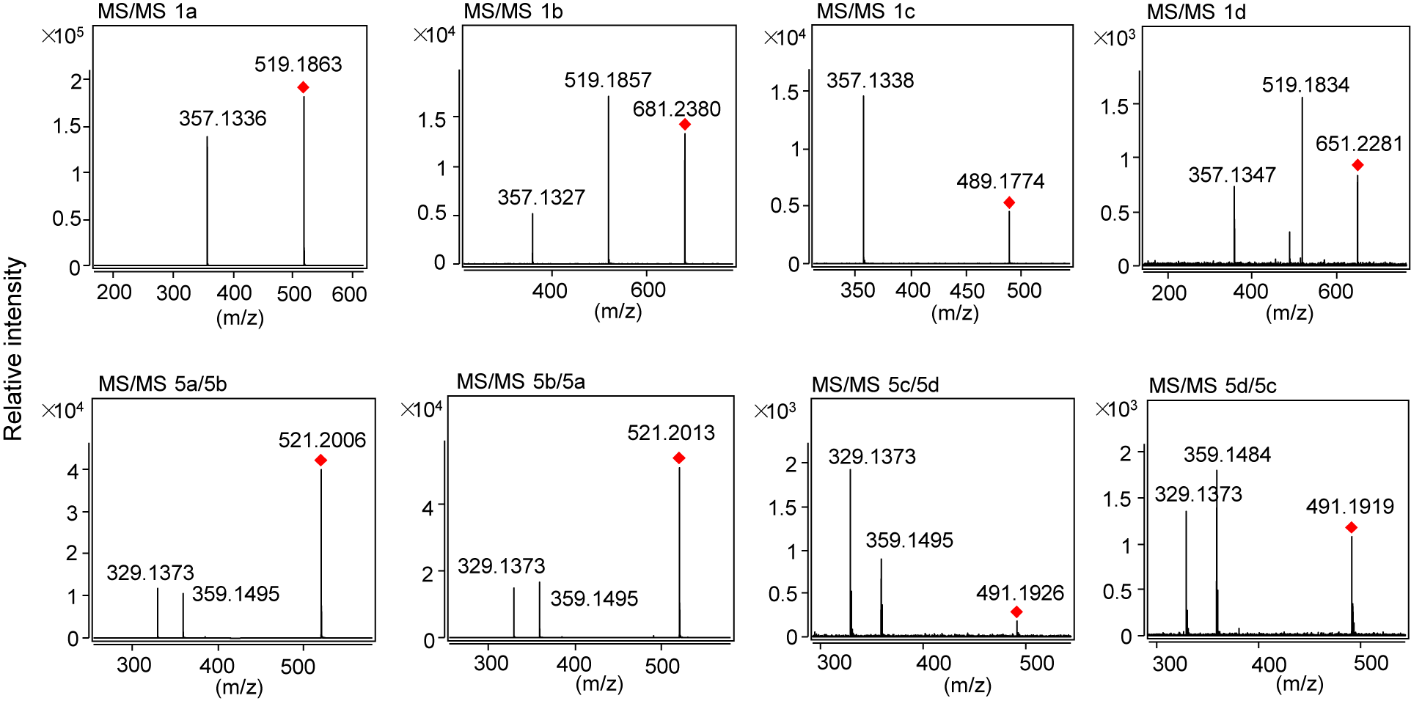


**Supplementary Figure 8.** The secondary mass spectra of the enzymatic reaction products of recombinant *Ii*UGT71B5a with different sugar donors in the negative ion mode of UHPLC-Q-TOF/MS.

The annotation of each chemical is consistent with Figure 6.


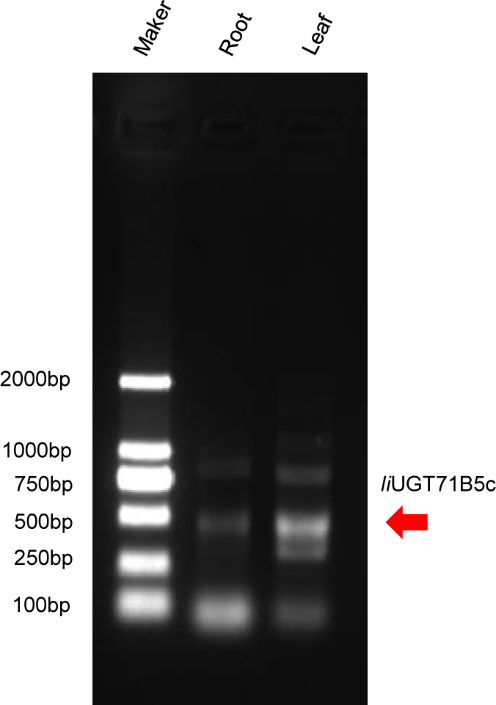


**Supplementary Figure 9.** Electropherogram of the PCR product of *Ii*UGT71B5c.

The ORF region of *Ii*UGT71B5c was 435 bp. PCR templates included total RNA from the leaves and roots of *Isatis indigotica*.


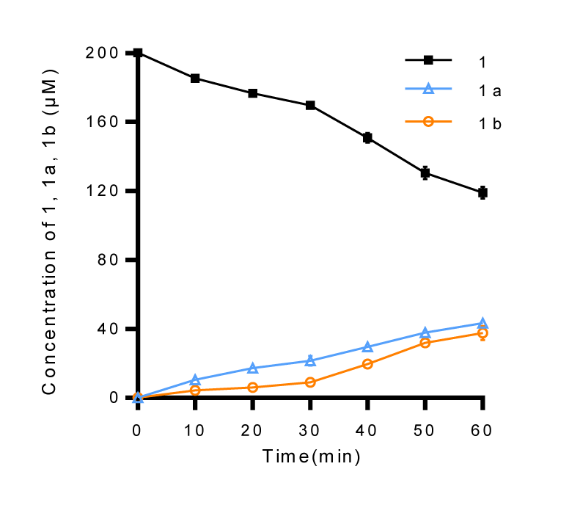


**Supplementary Figure 10.** The concentration of pinoresinol (1), pinoresinol-4-*O*-glucoside (1a) and pinoresinol diglucoside (1b) changes with the reaction time (0-60 min).

The ordinate value is expressed as the average value, and the error bar indicates SD (n = 3).


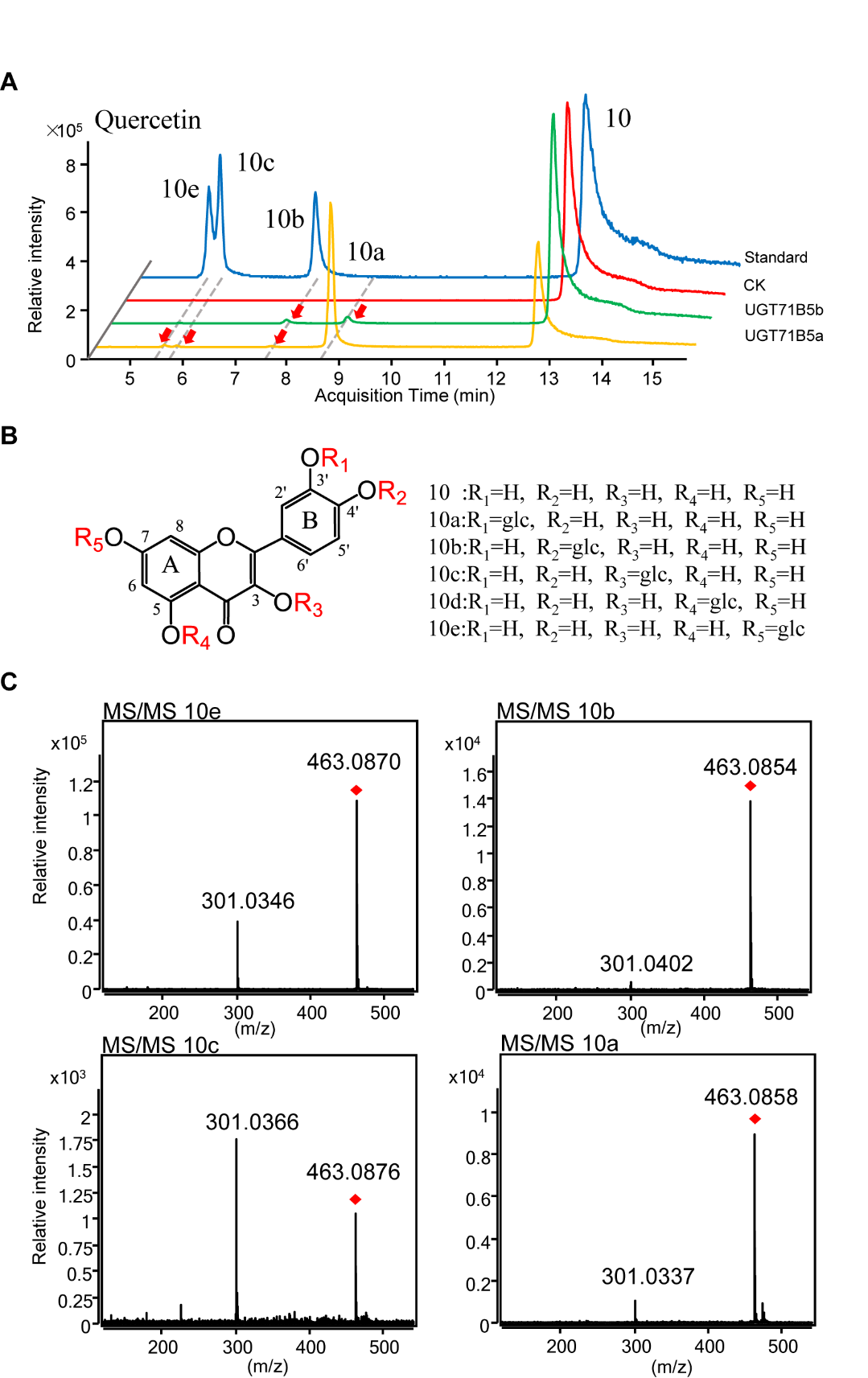


### Supplementary Figure 11. UHPLC-Q-TOF/MS analysis of glycosylation of quercetin (10) by recombinant UGT71B5a and UGT71B5b.

**(A)** HPLC chromatograms of the enzymatic reactions. **(B)** The chemical structure of quercetin and its glucosides (10: quercetin, 10a: quercetin-3’-*O*-glucoside, 10b: quercetin-4’-*O*-glucoside, 10c: quercetin-3-*O*-glucoside, 10d: quercetin-5-*O*-glucoside, 10e: quercetin-7-*O*-glucoside). **(C)** Typical positive ion MS spectra for peaks of 10a, 10b, 10c and 10e.


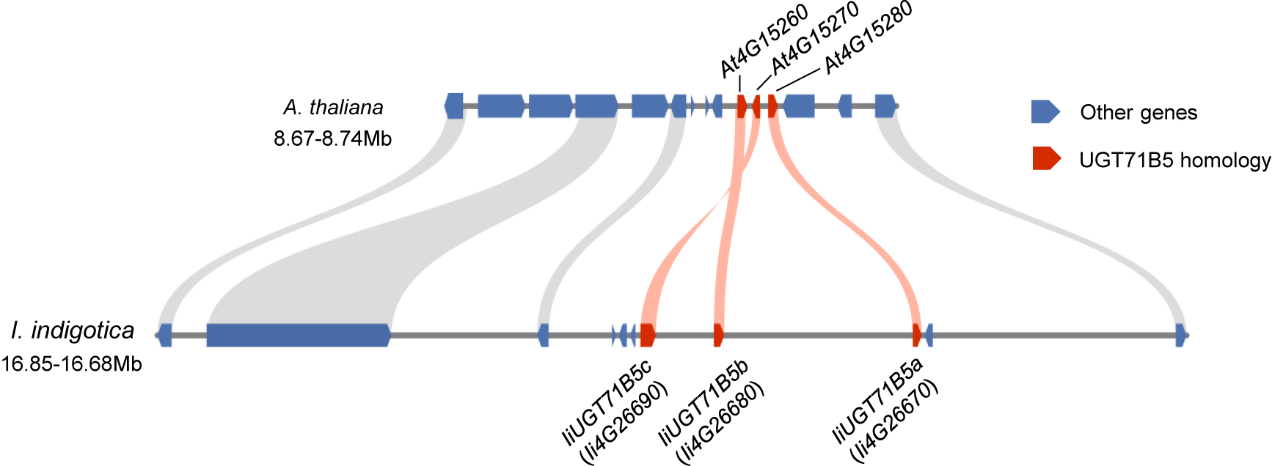


**Supplementary Figure 12.** Tandem repeats of *IiUGT71B5* genes on chromosome 4 of *I. indigotica*.

Genomic organization and syntenic relationships of *IiUGT71B5* genes in *A. thaliana* and *I. indigotica*. Wedges connect matching gene pairs, with *UGT71B5s* set highlighted in red.


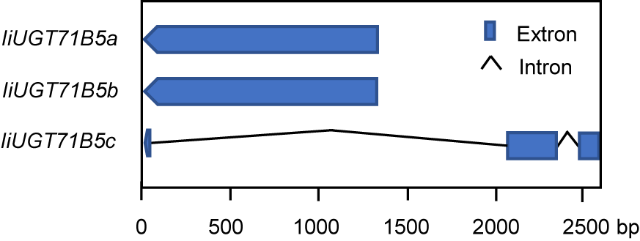


**Supplementary Figure 13.** The genomic structures of three *IiUGT71B5* genes from *I. indigotica.*

## Supplementary Tables

| **Supplementary Table 1**. Mass spectrometry parameters of the target compound in the negative ion mode of UHPLC-Q-TOF/MS | | | | | |
| --- | --- | --- | --- | --- | --- |
| **Number** | **Compounds** | **Molecular formula** | **Molecular mass** | **Ionic species** | **m/z** |
| 1 | Pinoresinol | C_20_H_22_O_6_ | 358.1416 | [M-H]^-^ | 357.1344 |
| 2 | (+)-Pinoresinol-4-*O*-glucoside | C_26_H_32_O_11_ | 520.1945 | [M-H]^-^ | 519.1872 |
| 3 | Pinoresinol diglucoside | C_32_H_42_O_16_ | 682.2473 | [M-H]^-^ | 681.2400 |
| 4 | Lariciresinol | C_20_H_24_O_6_ | 360.1573 | [M-H]^-^ | 359.1500 |
| 5 | Lariciresinol-4-*O*-glucoside | C_26_H_34_O_11_ | 522.2101 | [M-H]^-^ | 521.2028 |
| 6 | Lariciresinol-4′-*O*-glucoside | C_26_H_34_O_11_ | 522.2101 | [M-H]^-^ | 521.2028 |
| 7 | Lariciresinol diglucoside | C_32_H_44_O_16_ | 684.2629 | [M-H]^-^ | 683.2557 |
| 8 | Secoisolariciresinol | C_20_H_26_O_6_ | 362.1729 | [M-H]^-^ | 361.1657 |
| 9 | Secoisolariciresinol monoglucoside | C_26_H_36_O_11_ | 524.2258 | [M-H]^-^ | 523.2185 |
| 10 | Secoisolariciresinol diglucoside | C_32_H_46_O_16_ | 686.2786 | [M-H]^-^ | 685.2713 |
| 11 | Isolariciresinol | C_20_H_24_O_6_ | 360.1573 | [M-H]^-^ | 359.1500 |
| 12 | (-)-Isolariciresinol-9′-*O*-glucoside | C_26_H_34_O_11_ | 522.2101 | [M-H]^-^ | 521.2028 |
| 13 | (-)-Isolariciresinol-4′-*O*-glucoside | C_26_H_34_O_11_ | 522.2101 | [M-H]^-^ | 521.1660 |
| 14 | (-)-Isolariciresinol-4-*O*-glucoside | C_26_H_34_O_11_ | 522.2101 | [M-H]^-^ | 521.1660 |
| 15 | Isolariciresinol diglucoside | C_32_H_44_O_16_ | 684.2629 | [M-H]^-^ | 683.2557 |
| 16 | Matairesinol | C_20_H_22_O_6_ | 358.1416 | [M-H]^-^ | 357.1344 |
| 17 | Matairesinol-4-*O*-glucoside | C_26_H_32_O_11_ | 520.1945 | [M-H]^-^ | 519.1872 |
| 18 | Matairesinol-4′-*O*-glucoside | C_26_H_32_O_11_ | 520.1945 | [M-H]^-^ | 519.1872 |
| 19 | Matairesinol diglucoside | C_32_H_42_O_16_ | 682.2473 | [M-H]^-^ | 681.2400 |
| 20 | Clemaphenol A | C_20_H_22_O_6_ | 358.1416 | [M-H]^-^ | 357.1344 |
| 21 | (+)-Clemaphenol-3-*O*-glucoside | C_26_H_32_O_11_ | 520.1945 | [M-H]^-^ | 519.1872 |
| 22 | Clemaphenol diglucoside | C_32_H_42_O_16_ | 682.2473 | [M-H]^-^ | 681.2400 |
| 23 | Coniferyl alcohol | C_10_H_12_O_3_ | 180.0786 | [M-H]^-^ | 179.0714 |
| 24 | trans-Coniferin | C_16_H_22_O_8_ | 342.1315 | [M+COOH]^-^ | 387.1297 |
| 25 | Sesaminol | C_20_H_18_O_7_ | 370.1053 | [M-H]^-^ | 369.0980 |
| 26 | (+)-Sesaminol-2′-*O*-glucoside | C_26_H_28_O_12_ | 532.1581 | [M+COOH]^-^ | 577.1563 |
| 27 | Phillygenin | C_21_H_24_O_6_ | 372.1573 | [M-H]^-^ | 371.1500 |
| 28 | Forsythin | C_27_H_34_O_11_ | 534.2101 | [M+COOH]^-^ | 579.2083 |
| 29 | Quercetin | C_15_H_10_O_7_ | 302.0432 | [M-H]^-^ | 301.0354 |
| 30 | Quercetin-3-*O*-glucoside | C_21_H_20_O_12_ | 464.0960 | [M-H]^-^ | 463.0882 |
| 31 | Quercetin-7-*O*-glucoside | C_21_H_20_O_12_ | 464.0960 | [M-H]^-^ | 463.0882 |
| 32 | Quercetin-4′-*O*-glucoside | C_21_H_20_O_12_ | 464.0960 | [M-H]^-^ | 463.0882 |
| 33 | Quercetin-3′-*O*-glucoside | C_21_H_20_O_12_ | 464.0960 | [M-H]^-^ | 463.0882 |

| **Supplementary Table 2**. Mass spectrometry detection conditions for standard products in LC/MS negative ion mode | | | | | | |
| --- | --- | --- | --- | --- | --- | --- |
| **Compound Name** | **Precursor ion** | **Product ion** | **Dwell** | **Fragmentor** | **Collision Energe** | **Polarity** |
| Secoisolariciresinol diglucoside | 685.3 | 523.1 | 50 | 240 | 35 | Negative |
| Pinoresinol diglucoside | 681.2 | 519.2 | 50 | 120 | 7 | Negative |
| Forsythin | 533.2 | 371.2 | 50 | 105 | 5 | Negative |
| Secoisolariciresinol monoglucoside | 523.2 | 361.2 | 50 | 100 | 26 | Negative |
| (-)-Isolariciresinol-9′-*O*-glucoside | 521.2 | 315.1 | 50 | 95 | 24 | Negative |
| Matairesinol monoglucoside | 519.2 | 357.2 | 50 | 120 | 10 | Negative |
| Matairesinol glucoside | 519.1 | 357.0 | 50 | 120 | 9 | Negative |
| (+)-Pinoresinol-4-*O*-glucoside | 519.1 | 357.0 | 50 | 120 | 9 | Negative |
| Phillygenin | 371.2 | 356.1 | 50 | 120 | 18 | Negative |
| Sesaminol | 369.1 | 340.1 | 50 | 160 | 24 | Negative |
| Secoisolariciresinol | 361.1 | 164.9 | 50 | 135 | 30 | Negative |
| Isolariciresinol | 359.2 | 344.0 | 50 | 160 | 21 | Negative |
| Lariciresinol | 359.2 | 329.0 | 50 | 100 | 3 | Negative |
| Clemaphenol A | 357.2 | 151.1 | 50 | 120 | 20 | Negative |
| Pinoresinol | 357.1 | 151.1 | 50 | 135 | 20 | Negative |
| Matairesinol | 357.1 | 121.9 | 50 | 135 | 26 | Negative |
| Piperitol | 355.1 | 340.1 | 50 | 120 | 18 | Negative |
| trans-Coniferin | 341.1 | 179.1 | 50 | 85 | 9 | Negative |
| Warfarin | 306.7 | 250.0 | 50 | 130 | 20 | Negative |
| Coniferyl alcohol | 179.0 | 146.0 | 50 | 75 | 5 | Negative |

| **Supplementary Table 3**. Primers used in this study | | |
| --- | --- | --- |
| **Function** | **Primer name** | **Sequence (5'- 3')** |
| Plasmids construction for recombinant proteins | pET32a^+^-*Ii*UGT71B5a -F | CGACGACAAGGCCATGGCTATGAAGATCGAGCTC |
|  | pET32a^+^-*Ii*UGT71B5a -R | gtggtggtggtgctcgaggaccacaacAttctcg |
|  | pET32a^+^-*Ii*UGT71B5b -F | CGACGACAAGGCCATGGCTATGAAGATCGAGCTTG |
|  | pET32a^+^-*Ii*UGT71B5b -R | gtggtggtggtgGCGGCCGCAGCCGCAACATTCTCG |
| Real-time quantitative PCR | q-*β*-actin-F | ATCCTCCGTCTTGACCTTGC |
|  | q-*β*-actin-R | TTTCCCGTTCTGCTGTTGTG |
|  | q-*Ii*UGT71B5a -F | GTGGTGACGCCACTGACTCC |
|  | q-*Ii*UGT71B5a -R | TAGATCTATCATCGAGGTAC |
|  | q-*Ii*UGT71B5b -F | ACTCATCGATCCAACACGAGT |
|  | q-*Ii*UGT71B5b -R | CAAGTAATCCTTGGAAATGAGG |
| Transient expression in *Nicotiana benthamiana* | PHB-YFP-*Ii*UGT71B5a -F | GACGAGCTGTACAAGGGATCCATGAAGATCGAGCTC |
|  | PHB-YFP-*Ii*UGT71B5a -R | CTAGAGGATCAATTCGAGCTCGACCACAACATTCTCG |
|  | PHB-YFP-*Ii*UGT71B5b -F | GACGAGCTGTACAAGGGATCCATGAAGATCGAGCTTG |
|  | PHB-YFP-*Ii*UGT71B5b -R | CTAGAGGATCAATTCGAGCTCAGCCGCAACATTCTCG |

| **Supplementary Table 4**. One hundred eighteen putative genes encoding UGTs with complete coding sequences (CDSs) | |
| --- | --- |
| **UGTs**  **(Accession numbers)** | **Sequences** |
| *Ii*UGT71B5a | MKIELVFIPSPGIGHLRSTVELAKQLVNGDDRLSITVIIIPRSSGGDATDSAQISSLFAASQDRLRYETISVADEPTADRLPTQLYIKNQKPQVRDAVAKILDPARVDSPPRLAGFVVDMFCTSMIDLADEFGVPTYMVYTSNATFLGITLHLQLMLDEKKYDTSELDESVNELEFPCLTRPYPVECLPYLFISKEWLPFFMDQARSFRKMKGILVNTVAELEPHALELFSGDDDLPRAYPVGPVLHLESGSDNSNDDGKQSEILRWLDDQPAKSVVFLCFGSMGGFNEEQTREIAVALDRSGHRFLWSLRRASPDILKQGPGDYTDLDEVLPEGFLDRTLERGKIIGWAPQAAVLAKPAIGGFVTHCGWNSMLESLWFGVPMVTWPLYAEQKVNAFVMVEELGLAVEIRRSLKGDLMAGGMETVAAEDIERAIRRVMEQDSDVRNRVKEMAEKCHVALTDGGSSQVALRKFIQDVIENVVV* |
| *Ii*UGT71B5b | MKIELVFIPSPGIGHLRSTVELAKQLVNGDDGLSITVIIIPRSSGGDATAQISSLFAASQDRLRYETISVADRPTADPLPTQFYIENQKPQVRDAVAKILDPARVGSPPRLAGFVVDMFCTSMIDVADEFGVPTYMVYTSNATFLGITLHLQLMIDEKKYDTSELDESVNELEFPCLTRPYPVDCLPHILTSKEWLPLFMDQARSFRKMKGILVNTVAELEPHALELFSGDGDLPRAYPVGPVLHLESGSDNSNDDGKQSEILRWLDEQPAKSVVFLCFGSMGGFNEEQAREIAVALDRSGHRFLWSLRRASPDIMKERPGDYTNLDEVLPEGFLDRTSERGKIIGWAPQVAVLAKPAIGGFVTHCGWNSMLESLWFGVPTVTWPLYAEQKVNAFEMVEELGLAVEIRRSLKGDLMAGRMETVAAEDIERAIRRVMEQDSDVRNRVKEMAEKCHVALTDGGSSKAALRKFIEDVVENVAA* |
| *Ii*UGT71B5c | mkielvfipfpeighlrstvelakqlvdrddrlsitviiiprssgedagqayienqkpqvrdavaklidparvdspsrlagfvvdmfciamidvadefgvptymvytsnatflgitlhlqlmydekkydttelgesvnelvfpcr* |
| *Ii*UGT71C5 | MKTSELIFVPLPETGHLLSTIEFGKRLLDLDRRISMITILSMKLPYAPHADASLASLTASEPGIRLISLPEIQDPPPIKLLDTSSETYILDFVEKNIPFLRKTIRDLVSSSGEDSNHVAGLILDFFCVDLIDIGREVNLPSYIFMTSNFGFLGFLQYLPERHRSISSEFDESSGDEELPIPAFVNRVPAKVLPPGVFDKLSYGTLVKIGERLNEAKGIFVNSFSEVEPYAAEHFSRGGDYPRAYPVGPVLNLTGRTNPGLASAQYAEMMKWLDDQPDSSVLFLCFGSMGVFSAAQITEIAHAIELVGFRFIWAIRTNMEGDGDPHEPLPEGFVDRTMGRGIVCSWAPQVDILAHKATGGFVSHCGWNSIQESLWYGVPIATWPMYAEQQLNAFEMVKELGLAVEIRLDYVADGDRVTLEIVSADEIAAAIRSLMDGDNLIRKKVREVSAAARKAVSDGGSSMVATGDFIRDILGDHF* |
| *Ii*UGT71C8 | MVTILTMKLPYAPNADAYLAALTASEPRIRYIRLPEVQDTSSETDILDLVDKNIPSVRKTIQDLVSSSSEGHVAALILDFFCVGFIDIGREVNLPSYIFMTSNFGFLGLLQYLPERHRLTPSEFDVSSGNEELSIPVFVNKVSAKVLPPAVFDKLYYKTYVKIGERLHEATGILVNSFSDVEPYAAEHFSRGRDYPRVYPIGPVLDLTGRSITCLSSEQYEEMMKWLDEQPESSVLFLCFGSMGVFSAAQIKQIAHALELVGFRFIWAIRTNMAGDGDPHEPLPEGFVDRTVGRGVVCSWAPQVEILAHKAIGGFVSHCGWNSIQESLWYGVPITTWPMYAEQQLNAFSMVKELGLAVEIRLDYVADGDNVTLEIVSAEEIATAVRSLMDGDNPVREKVKEISAMARKTLGDGGSSMVATGDFIRDVLGDHF* |
| *Ii*UGT71C4 | MAKETELIFIPVPSTGHLLVNIEFAKRLINLEHRIQTITIIQMDSPINPHASVFARSLVASQPQIRLHSLPVLHDPPPSDLYKRAPEAYIVQLVKKTTPLVKDAVSSIVESRGSDSVRVAGLVLDFFCNSLIKDVGNELNLPTYIFLTCNARYLSMMKYIPDRHRKMASKLDWSSGDEELPIPGFANPIPTKFMPPGLFNQEGYEAYVELAPRFAHAKGILVNSIAELEPHPFGYFSQQHNYPPVYPVGPILSLKDRASPNEEEAADRDRILRWLEDQPESSVVFLCFGSKGSVDEPQVKEIAQALEVVGCRFLWSIRMSLEEIKPSDVLPEGFMGRVAGRGLVCGWAPQVEVLAHKAIGGFVSHCGWNSTLESLWFGVPVATWPMYAEQQLNAFTLVKELGLAVDLRMDYVSGRGGLVTCDEIARAVRSLMDGGEGKRVKVKEMADAARKAMMDGGSSYLATARFIGELLDDGSS* |
| *Ii*UGT90A2a | MEPEKVHVVLFPYMSKGHMIPMLQLARLLLSHSFAGDISVTVFTTPSNRPFVADSLSGTKATIIAVPFPKNVPEIPPGVECTDKLPAISSLFVPFTRATKSMQPDFERELMSLPRVSFMVSDGFLWWTLESARKLGFPRLVFLGMNCASTSILDSVFQNQLLSKVKSETEPVSVPEFPWIKVRKCDFVKDLFDPKPTTDPGFKLILDQVTSTNQSQGIIFNTFDDLEPVFIDFYKRKRELKPWTLGPLCFVNNSLVDKGEEKFKSVWMKWLDEKRDRGCYVLYVAFGTQAEISREQVQEIAFGLEESKVNFLWVVKGNEVGEGFEGRVGDRGMVVKDEWVDQRKILEHESVRGFLSHCGWNSLMESICSEVPILAFPVAAEQPLNAILVVEELRVAERVVPASERIVRRKEIAEKVKELMEGEKGKELRRNVEAYGKMAKKALEEGIGSSWKNLDKLINEFCNNGA* |
| *Ii*UGT90A2b | MEPEKVHVVLFPYMSKGHMIPMLQLARLLLSHSFAGDISVTVFTTPSNRPFVADSLSGTKATIIAVPFPKNVPEIPPGVECTDKLPAISSLFVPFTRATKSMQPDFERELMSLPRVSFMVSDGFLWWTLESARKLGFPRLVFLGMNCASTSILDSVFQNQLLSKVKSETEPVSVPEFPWIKVRKCDFVKDLFDPKPTTDPGFKLILDQVTSTNQSQGIIFNTFDDLEPVFIDFYKRKRELKPWTLGPLCFVNNSLVDKGEEKFKSVWMKWLDEKRDRGCYVLYVAFGTQAEISREQVDEIAFGLEESKVNFLWVVKGNEIGEGFEGRVGDRGMVVKDEWVDQRKILEHESVRGFLSHCGWNSLMESICSEVPILAFPVAAEQPLNAILVVEELRVAERVVPASERIVRRKEIAEKVKELMEGEKGKELRRNVEAYGKMAKKALEEGTGSSWKNLDKLINEFCNNGA* |
| *Ii*UGT75D1 | MDNNNDSSKSPTGPHFLFVTYPTQGHINPSLELAKRIAAIITGARVTFAAPISACNRRMFSKENVPETLIFATYSDGHDDGYKASTSSDKSRQDTARQYMSEMRRRGRETLTELIEDNRRQNRPFTCVVYTILLTWVAELARDFHIPSALLWVQPVTVFSIFYHYFSGYADAISEMASNNPSGSIELPSLPPLRLRDLPTFIVPENTYAFLLSAFREQIESLKQEENPKILVNSFQELEQEALSSVLDNFKIIPIGPLITSRTDSGTGAEYVEWLDTKTDSSVLYISFGTLAVLSTRQLVELCMALIQSRRPFLWVITDKTYRIKEDGEETEEESIRSFREELDEIGMVVSWCDQFSVLKHRSIGCYVTHCGWNSSLESLVAGVPVVAFPQWTDQTTNAKLLEDCWRTGVRVMEKKEDEEVVVESGEIRRCIEEVMDKKSEEFRENAARWRDLAAETVREGGSSFNHLKAFVDEHM* |
| *Ii*UGT84A4 | MESSLTHVMLVSFPSQGHINPLLRLGKLIASKGLLVTFVTTEEPLGKKMRQANEIQDGLLKPVGSGFLRFEFFDDGYTHDDLENKHTSGLLLTDLEVAGKREIKNLVKRYEEQKQPVRCLIFNAFVPWVCDVADDLQIPSAVLWVQSCACLAAYYYYQNQLVKFPTKTEPEINVEVPFMPSVLKHDEIPSFLHPSSPFSVYAETILQQIKRLPKSVSVLIETFEELERDIIDHMSELCPEVIINPIGPLFMMAKNTSSDIKGDISGSTDQCMEWLDSKEPSSIVYISFGTVVHLKQEQIDEIAHGLLSSGLSFLWVVRPPMEGLSVEPYVLPRELEEKGKIVEWCPQDRVLAHPAVACFLSHCGWNSTIEALSSAVPVVCLPQWGDQVTNALYLVDVFKTGVRLGRGGTEEKIVSREVVAEKLLEAAIGEKAVELRENARRWKNKAEAAVGNGGSSDRNFGGFVDKLVANIW* |
| *Ii*UGT84A3 | MEPSLHAHVMLVSFPGQGHINPLLRLGKLIASKGLLVTFVTTEQPWGKKMRQANKIQDGVLKPVGLGFLRFEFFHDGLTDDDEKRTDFHAFRPHLELVGKREITSLIKRYEQEEKQPVRFLINNAFVPWVCDVADDLQIPSAVLWVQSCACLAAYYYYHHRLVKFPTKTEPETNVEIPSLPLLKHDEIPSFLHPSSVLAAYGEVILDQFKRLENHKPLFLFIDTFQELEKDIIGHMSNLCPQVNFSSVGPLFKMAQTISSDVKGDISAPASHCMEWLDSREPSSVVYISFGTVAHLKQEQIEEIAYGVLSSGLSFLWVVRPPMEGLSVEPYVLPRELEERGKIVEWCSQERVLAHPAIACFLSHCGWNSTMEALSSGVPVVCLPQWGDQVTDAVYLVDVFKTGLRLGRGAAEDKIVLREVVAEKLLEATVGKKAVELRENARRWKEQAEAAVAYGGSSDRNFQEFVDKLVTKPVVGKDDGKHE* |
| *Ii*UGT84A1a | MGSISVMEFETSSSPTPVHVMLVSFQGQGHVNPLLRLGKLIASKGALVTFVTTELWGKKMRQANKIVDGELKPVGSGSLRFEFFDEEWAEDDDRRGDFLVYISHLEQIGKREVSKLVRRYEEDKEPVSCLINNPFIPWVCDVAEEFNIPSAVLWVQSCVCFSAYYHYQTGSVPFPTETEPELDVEIPCVPLLKHDEVPSFLHPSSPLAGLGEAVLGQFKNLSKPFCVLVDSFDALEQEVIDYMSSLCPIKTVGPLFKVAKTVTTDVSGDICKPADQCLEWLDSRPKSSVVYISFGTVAYLKQEQIEEIAHGVLKAGLSFLWVIRPPPHELKVETHVLPQELEEESGKGKGKIVEWCPQEEVLAHPSVACFVTHCGWNSTMEVLSSGVPVVCFPQWGDQVTDAVYLVDVFKTGVRLGRGATEERVVPREEVAEKLLEATVGEKAEELRNNALKWKAEAEAAVAPGGSSDKNFQEFVEKLGVGVSKVKENGH* |
| *Ii*UGT84A1b | MRWRFYKSRILLLHVKFAITPSLLFFVDNKMEFETSSPPNPIHIMLVSFIGQGSVSPMLRLGKLIASKGTTVTFVTTEVWGKKMKQANKIVDGELKPVGSGSIRFEFFDDEWSEDDERRGNFALYIPQLEQIGKREVSKLVRRYEEKNEPVSCLINNPFVPWVGDVAEELNIPFAMLWIQSCACFSAFYHYQNGSVPFPTAEEPKLDVKLPCVPVLKYDEIHTFLHPFSPQTGLRKAILGQFETLSKPFCILVSSFDALEQEVIDYMSKLCPIKTVGPLFKVAKTVKSDVSGDFCKPSDQCLDWLDSRPKSSVVYISFGTVAYLQQEQMEEMARGVLKSGVSFLWVIRPPLRDLRLESHVVPPELKEASDRGKGKIVDWCPQEQVLAHPSVACFVTHCGWNSTTEALTSGVPVVCFPQWGDQVTNAVYLVDVFKTAVRLGRGAADGRIVPGEEVAEKLLEATVGEKAEELRKNALKLKAEAEAAVAPGGSSDKNFQEFMEKLGVSSGGSK* |
| *Ii*UGT84A1c | MQNPSTATQESAASAFGFFTRLSSLASRNRSPCSSSDGYALSTSNALFFNGSRTLPARRTRKPLASLSLNTKSGAGSSLRRFIGEFNSFIRFHCDKVVPDSFASVGGVGVSSDENGVRENDAGGVSGEEGLPLNGVEADRPKKVLILMSDTGGGHRASAEAIRAAFNEEFGDEYQVFITDLWTDHTPWPFNQLPRSYNFLVKHGTLWKMTYYGTAPRVIHQSNFAATSTFIAREIAQGLMKYQPDIIISVHPLMQHVPLRVLKSKGLLDKIVFTTVITDLSTCHPTWFHKLVTRCYCPSTEVAKRAQKAGLGTSQIKVYGLPVRPSFVKPVRPKVELRRELGMDENLPAVLLMGGGEGMGPIEATARALGDALYDESLGEAVGQVLIICGRNKKLQTRLSSLDWKIPVQVKGFVTIMEECMGACDCIITKAGPGTIAEAMIRGLPIILNGYIAGQEAGNVPYVVENGCGKFSKSPKEISKIVADWFGPGSKQLEIMSQNALRLARPEAVFKIVQDMHELVRQRNCLPQLSCTA* |
| *Ii*UGT73B2 | MRVATSCEPFVIPDLPGNIVITQGQIRDRDEETEMGKFMVEVIESEVKSSGVVVNSFYELEPDYADFYKSTIAKRAWHIGPLSIHNRGIEEKAERGKKANIDEVECLKWLDSKKPNSVIYISFGSVASIKNEQLIEIAAGLEASGTSFIWVVRKNGEKSGDKEEWLPEGFEERVKGRGMIIRGWAPQVLILDHQATGVFVTHCGWNSLLEGVAAGLPMVTWPVGAEQFYNEILVTQVLRTGVSVGTKKHMNMGDFVTRENVDKAVREVLVGEEAEERRRRAKKLAEMAKAAVEEGGSSYNDLSSFIEEFSS* |
| *Ii*UGT73B3a | MSGNPHRKLHVVFFPFMAYGHMIPTLDMAKLFSSRGAKSTIITTPLNSKILQKPIDVFKNLNPSLEIDIQIFDFPCVELGLPEGCENVDFFTSNNNDDTGYITFKFFLSTRFFKDQLEKLLEKTRPDCLIADMFFPWATEAAEKSHVPRFVFHGTGYFSLCAGYCIKVHKPQVRVASSCEPFVIPDLPGDIVITQEQIIDGDNESEMGKFMFDVRESELKSSGVVVNSFYELEPDYADFYKRFVAKRAWHIGPLSVINRGFEEKAERGKKASIDEAECLKWLDSKKPDSVIYISFGSVACFKKEQLIEIATGLEASGANFIWVVRKYTGDEEEWLPEGMEERTKGKGMIIRGWAPQVLILEHQATGGFVTHCGWNSLLEGVAAGLPMVTWPIAAEQFYNEKLVTQVLRTGVSVGATKHVKVMEDEVMSREKVEKAVREVLVGDEAEEMRKRAKKLAEMAKAAVEDGGSSFNDLNSFIQEFSS* |
| *Ii*UGT82A1 | MKVIQKPKIIFIPYPAQGHVTPMLHLASAFLSRGFSPVVMTPESIHRRISMTNEDLGITFLALSDGQERPDAPPSDFFSIEKSMENIMPSQLERFLLEEDVGVACVVVDLLASWAIRVADQCGVPVAGFWPVMFAAYRMIEAIPELVRTGIVCRKGCPRQLEKPLLLPEQPLLAAGDLLWLIGTPAARKGRFKFWQRTLERSKSLRWILVNSFKDEYEGEFNKEYNDRNPQIFYVGPLHNQQATTDKTLTKNPSFWEEDRSCLGWLQEQKPNSVIYISFGSWVSPIGESKIKTLALALEASGRPFIWALNRAWQEGLPPGFIRRVTIAKNQGRIVPWAPQIEVLKNDSVGCYVTHCGWNSTMEAVASSRRLVCYPVAGDQFVNCRYIVDVWKIGVRMSGFGEKEVEDGLRKVMEDQEMGERLKKLREKAMGHEARLCLDKSFTLFKDEIR* |
| *Ii*UGT72C1 | MGFHGTLVASPGMGHAVPILEFGKRLLNHHGFDRVTVFLVTDDDSRSKSLFGKMLRDKDPKFVIRFIQLDLSGQDLSGSLLTKLAEMMRKAIPQIRSAVMGLQLQPNVFVVDLLGTEALAVAKELEITKKHVLVTTSAWFLAFTVYMASLDKETLYKHLSSHGALLIPGCSPVKFDRVQDPSSYVRELAESQRIGAGVIAADGVFVNTWHSLEPVTIRSFSDPENLGRVMRGVPVYPVGPLVKSAEPDLKHDVLDWLDLQPKESVVYISFGSGGALTAEQTIELAYGLELTGGRFVWVVRPPAEDDPSASMFDKTTNQTEPLDFLPDGFLDRTKNIGLVVRTWAPQEEILAHESTGGFVTHCGWNSVLESIVNGVPMIAWPLYSEQKMNAWLVSEELKIAARVNVGNGIVKKEEIIVMVKRVMDEEEGKEMRKNVKELKKKTAEEALKKLPTP* |
| *Ii*UGT89A2c | MPGEMPPRPPQESKGCVLKPHIMVFPYPAQGHLLHMLDLTHQLCLNGVIVSIIVTPRNLPYLSSLLTAHPSAVSAVTLPFPHHPSLPSDVENVKELGGSGHLMIMASLRLLREPIMNWLSSHPNPPVALISDFFLGWTNDPGIPRFAFFTSGAFLASVLHFVSDKLHLFESTEPVCFSHLPRSPVFKTEHIPSVTPQSPSSRDIESVKDITMSFSSYGCIFNSCECLEEEYVEYAKQKVGHNLVFGVGPLYSIGLGEGDSGTDVDTKALVRWLDGCPDGSVLYICFGSQKVLSRDRPGPALSREQCDALALGLELSMIRFVWVAKTDPIPVVVLSHVAVGGFLCHCGWNSVLEATASGTMILAWPMEADQFVDARLLVEHMGVAVSVCEGGNTVLNPHELGRVIAETMGEKGREVRARAKEMGRDERVATEARGSSIVDLQRLVKELNSLTIKI* |
| *Ii*UGT74B1 | MAETRTNRSKGHVVVLPYPVQGHLNPMVQFAKRLVSKGVKVTIATTTYTASSISTPSVSVEPISDGYEFIPIGVPGVSIDAYSESFKLNGSETLTRVIEKFKSTDSPVDSLVYDSFLPWGLEVARSNSISGAAFFTNNLTVCSVLRKFAAGAFPLPADPNSAPYLVRGLPALSYDELPSFVGRHWLSHAEHGRVLLNQFPNHEDADWLFVNGFEGLETQDCETGESEAMKATLIGPVIPSAYLDGRIKDDESYGSSLMKPLSGECMEWLGTKPSQSVVFVSFGSFGILFEKQLAEVAIALQESNLNFLWVIKEAHIAKLPQGFVESTKDRALLVSWCNQLEVLAHESIGCFLTHCGWNSTLEGLSLGVPMVGVPQWSDQMNDAKFVEEVWKVGYRAKEEAGEGVVKSDEVVRCLKGVMEGETSVKIRESSKKWKCLAVKAMSEGGSSDRSINEFVESLGKKQ* |
| *Ii*UGT85A1 | MMNCLAPFEELICRINARDIVTPVSCIVSDDCMSFTLDAAEKLCVPHVLFWSTSACGLMAYLHFYHFIEKGLCPVKDESYITKEYLDTAIDWIPSMKNLKLKDIPSFIRTTDRDDVMLKFTLHEVERAKRTSAIILNTFDDLEHDVIQSIQSIISPVYSVGPLHLLEKQEIEDSSEVGRMGSNLWKEEMECLDWLDTKTPNSVVYVNFGSITVTSKKQLAEFAWGLAGSGKTFLWVIRPDLVAGEEAMVPPEFLVETRDRRMVVSWCPQEKVLSHQAIGGFLTHCGWNSMLESICAGVPMICWPFFAEQPTNCKFCCDEWGVGMEISEDVKREEIEAVVRELMDGEKGRTMREKAQKWRHLAEKATEHKFGDSYTNLETVVSKVLLRQGSED* |
| *Ii*UGT85A2b | MGSHAAFPAGKRHAVCVPYPAQGHINPMLKVAKLLHAKGFYVTFVNTIYNHKRLLRSRGPNALDGLPSFRFESIPDGLPETDVDVTQDIPALCESTMKNSLAPFKELLRRINAQDEVPPVSCIVSDGCMSFTLDAAEELGVPEVLFWTTSACGFLAYLHYHRFIEKGLSPLKVDLADDSYLTKEHLDTKIDWIPSMRNLRLKDIPSFIRTTNPNDIMLNFIIREAARATRASAIILNTFDDLEHDAIQSIQSILPPVYSIGPLHLLVKQEIVEDSEIGRMGSNLWKEETACMDWLNTKAQNSVVYVNFGSITVMTSKQLVEFAWGLAATGKDFLWVIRPDLVAGDVAMVPPQFLVETADRRMLASWCPQEKVLSHPAVGGFLTHSGWNSTLESITGGVPMVCWPFFAEQQTNCKYCCDEWEIGIEIGGDVKREEVEAVVRELMDGEKGKIMRERAEEWRRLAEKATECKHGSSVVNLDNVISKVLLRE* |
| *Ii*UGT85A7 | MASPTVCATQKPHAVCVPHPAQGHINPMLKVAKLLHAKGFHVTFVNTVYNHNRLLRSRGPNSLDGLPSFRFESIPDGLPETDGDKTQDIPALCISTMKNCLAPFKELLRRINARDDVPPVSCIVSDGVMTFTLDAAEELGIPDVVFWTASACGFMGFLQFHRFIEKGLSPFKDESSMSKEHLDTIIDWIPSMKNLRLKDIPSFIRTTDPDDVMLNFSIRELERCKRATAIILNTFDDLEHDVIQSMQSMLPPVYSIGPLHLLANREVDKASEMGQMGLNLWREETECLEWLDTKAPNSVVFVNFGCITVMSAKQLGEFAWGLAGSGKDFLWVVRPDLVAGEAAVVPPEFITDTMDRGMMASWCPQEKILSHPAIGGFLTHCGWNSTLESLCGGVPMICWPFFADQPTNCKFCCDEWGVGIEIGGDVKREEVEAVVRELMDGEKGKKLREKAEKWQRLAEEATEYPCGSSIVNFETLVDKVLLGTSHEILKG* |
| *Ii*UGT84A2 | MELSSSPLPPHVMLVSFPGQGHVNPLLRLGKLLASKGLLITFVTTESWGKKMRVANKIQDRVLKPIGKGYLRFDFFDDGLPEDDDASRTDFTILRPQLELVGQREIKNLVKRYEEVTKQPVTCFINNPFVSWVCDVAEDLQIPCAVLWVQSCACLASYYYYHHKLVDFPTKTHPETDVQIPCMPLLKHDEIPSFIHPSSPYSALREVIIDQIKRLHKPFAVLIDTFYSLEQEIIDHMTNLSLPGVVRPLGPLYKMAKTLICDDIKGDMSETTDHCIEWLDSQPVSSVVYISFGTVAYVKQEQINEIAFGVLNAGVSFLWVIRQQELGINKERHVLPEEVKGKGKIVEWCSQEKVLAHPSVVCFVTHCGWNSTMEALSSGVPTVCFPQWGDQVTDAVYMIDVLKTGVRLGRGETEERVVPREEVAERLREVTKGEKAMELKKNALKWKEEAEAAVARGGSSDRNLEEFVEKLGAKPVAKQNGSLDQNGSIQGLLLEKS* |
| *Ii*UGT88A1 | MEKQNAIVLYPSPPIGHLVSMVELGKLILSQNPSLSIHIILVPPPYQPESTATYISSVSSAFPSITFHRLPAVEPYSSTTSRKHHESLLLEILCFSNPNVHRTLFSISQTFNLCAMIIDFFCTAVLDVTADFTFPVYYFFTSGAACLASFFHLPILHETTAGKNLKDVHTLLSIPGVPPIKGSDMPKPVLDRKDEIYDAFIMFSKQLSNSSGIIINTFEELEKRAIKAITEELCFPNIYPIGPLIVNGRSGDKNGENADSCLNWLDSQPEQSVVFLCFGSLGLFSQEQLKEMAIGLERSEQRFLWVVRNPPELQNQTEPDLKSLLPEGFLDRTGNRGMVVKSWAPQVPVLNHKAIGGFVTHCGWNSILEAVCAGIPMVAWPLYAEQRFNRVVIVEEIKIAIPMNESETGFVSSTEVEKRVREIVEEGPVRERTKAMKNAAESALVETGSSRTALSALLQSWSPK* |
| *Ii*UGT76B1 | METRETKPVIFLFTFPLQGHLNPNFQLANILFNRGFSIIVIHTEFNSPNSSNFPHFTFISIPDGLSESEASNPDVIELLHDLNSKCVAPFGDCLKKLLSEEPTAACVIVDALWYFTDDLTEKFGIPRMVLRTVNLSAFVAFSKFHVLREKGYLSLQESQADLPVPELPHLRMKDLPWFQTEDPRSGDKLKTGVLKSLKSSSGIIFNAIEDLEPEQLDQSLKEFLVPFFCIGPFHRHVSPSSSSLLTQDMTCLSWLDKQEPKSVIYVSLGSIASIDESEFLEIAWGLRNSNQPFLWVVRPGSIHGTEWIEILPKGFIESLEGKGKIVKWAPQIEVLAHRATGGFLTHCGWNSTIESICEGVPMICKPSFGDQRVNARYISDVWRIGLHLENKIERLEIENAVRTLMTSSQGEEIRKRIMPMKETAEQCLKLGGSSFRNLENLIAYILSI* |
| *Ii*UGT80A2 | MPVEGSSSSDRAESSSSTKPRLNKSKTERQQKVTHILAEDAAKIFDDRISAGKKLKLLNRIATVKHDGTVEFEVPADAIPQPIAVDREESKNGVCPDESIDGIDLQYIPPMQIVMLIVGTRGDVQPFVAIAKRLQDYGHRVRLATHANFKEFVLTAGLEFYPLGGDPKVLAGYMVKNKGFLPSGPSEIPVQRNQMKDIIYSLLPACKEPDPDSGISFKADAIIANPPAYGHTHVAEALRIPIHVFFTMPWTPTSEFPHPLSRVKQPAGYRLSYQIVDSLIWLGIRDMVNDLRKKKLKLRPVTYLSGTQSSGSNIPHGYMWSPHLVPKPKDWGPQIDVVGFCFLDLASNYEPPAELVEWLEAGDKPIYIGFGSLPVQEPEVMTEIIVEALQRTKQRGIINKGWGGLGNLKEPKDFVYLLDNVPHDWLFPRCKAVVHHGGAGTTAAGLKAACPTTIVPFFGDQPFWGERVHARGVGPAPIPVDEFSLHKLEDAINFMLRDEVKSSAETLAKAMKDEDGVAGAVKAFFKHLPIMKQNISDPIPEPSGFLSFRKCFGLS* |
| *Ii*UGT72B2 | MAEANTTHIAIIPSPGVGHLIPLVEFAKRLVDHHRFTVTFIFLGESSPSRAQRSVLNSLPSSIASVFLHPVDLSDLPSTAGIETRISLTVTRSIPALRDLFASLSAEKRLPAVLVVDLFGTDAFDVAFEFHVSPYIFYPSNANVLSFMLHLPKLDELVSCEFRDLTEPLKIPGCVPITSKDFADPCQDRNDDAYKWLLHNAKRFKEAKGILLNSFAELEPNAIKALQEPAPDKPPVYPIGPLVNTGSSYADGSDEYECLKWLDNQPLGSVLYVSFGSGGTLTCEQLNELALGLAESGKRFIWVIRSPSGIANSSFFNSHSQTDPLTFLPPGFLDRTKGKGLVVPSWAPQVQILAHPSTGGFVSHCGWNSTLESIVNGVPLIAWPLYAEQKTNALLLVEDVRAALRARNSDDMIVRKEEVVRVVNSLMEGEEGKAIRNKTKELKEGVARVLRQDGLSTKALTEVSLKWKAHHREAEQDTTHTISSI* |
| *Ii*UGT75B1 | MAPPHFLLVTFPAQGHVNPSLRFARRLIRTTGARVTFVTCHSVFHRSMISKQSNLNNLSFLTFSDGFDERGISTDEDHQNRSVNLKINGDKTLSEFIEANRNGDSPVTCLVYTILLNWAPKVARRFQLPSALLWIQPALVFDIYYNHFNGSNSGFEFPKLPSLAILDLPSFLTPANTNKRAYAAFQELMEFLKEESNPKILVNTFDSLEQEALKAIPNIGMVAVGPLLPSDIFTGSESVKDLSRDESSYSRWLDSKTESSVIYVSFGTMVELSKKQIKELARALIEAKRPFLWVITDKPNRETKTEGEEEIEIEKIAGLRHELEEVGMIVSWCSQVEVLRHRAVGCFVTHCGWSSTLESLVLGVPVVAFPMWSDQPTNAKLLEDSWRTGVRVRENKEGLVEAGEIRRCLEAVVGEKAEELRKNAEEWQRLAVEAGREGGSLDKNMEAFVDEICGGIVQ* |
| *Ii*UGT89C1 | MAATTKKPHVLVVPFPQSGHMLPHLDLTHQLLIRGATVTVLVTPKNSPYLDPLRSLHSQEHFKTLILPFPSHPCIPSGVETLQQLPLQALPHMFEALSRLHDPLVDFLSRQPPSDLPDAILGSAFLSPWINKVADVFSLKSICFLPINAHSISIMWGQEDRGFFDELESSTTESYGLVVNTFYELEPEFIESVKTSFLTHHRAWTVGPLLAVKAGVDRGGRSSLPPEKVLAWLDTCGEDNSVVYIGFGSQIRLTVEQTAALAAALEKSGVRFIWAVRDAAKRVSSDDSPGEEDVIPAGFEERVKEKGLVIKGWAPQTMILEHRAVGSYLTHLGWGSVLEGMVGGVLLLAWPMQADHFYNTKLLVDDLGVAVRVGEDRDSVPDSDELARVLADSVKDDLPERVRLMKLRKKGMEAIKEGGSSCNSLDELVAEMCA* |
| *Ii*UGT86A2 | MADVKNPNNHHNHHLHALLIPYPFQGHVNPFVHLAIKLASQGITVTFVNTHYNHHQINSGDIFAGVRSESGLDIRYATVSDGLPIGFDRSLNHDQYQSSLLHVFSAHVEELVASLAGEGVNVIIADTFFVWPSVVARKFGLVCVSFWTEAALVFSLYYHMDLLRIHGHFGAQETRGDLIDYIPGVEAINPKDTASYLQETDISSVVHQIIFKAFEDVKKVDFVLCNTIHQFESKTIEALNSKIPFYAIGPIIPFNLKTGSSVTTSLWSESDCTQWLNTKPAGSVLYISFGSYAHVTKKDLVEIAHGILLSKVNFVWVVRPDIVSSDESNPLPEEFVSEAGDRGIVIPWCCQMTVLSHPSIGGFLTHCGWNSILESIWCEVPLLCFPLLTDQVTNRKLVVDDWEIGMNLCEDRSSVGRDEVKMSIDRLMCGKSREEVKGAIGRVKMSLEGALRCSGSSERNLGLFVDGLVAKVGLSNGKA* |
| *Ii*UGT187A2 | MDPTESQRVRNRHVVAMSWPGRGHINPMMNLCKRLVLRDPNLTVTFVVTEEWLGFIGSDPKPDRIHFATLPNLIPSELVRANDFTGFVNAVHTRLEEPFERLLDRLNSPPPTAIIADTYVIWGVRVGERKNIPVVSFWTMSATILSLFLHSDLLISHGHALFQPSEPKEEEIVDYIPGLPPTKLQDLPPVFDSKIHEGFKKSKLCFGEIPRAKSLLFTTAYELEPKAIDVFTSKLDIPVYATGPSIPFQELSVENERNKPDYIRWLDEQPESSVLYISQGSFLSVSEAQMEEIVAGVRESGVPFLWVGRGGESKLKEALEGSSGVVVSWCDQLRVLCHAAVGGFWTHCGYNSTLEGIYSGVAMLTFPLSWDQFLNAKMIVEEWRIGMRIEGKKKTIRRDEIKDMVKRFLDGESEEGKEMRRRVCDLSEICRGALAKTGSSDVNIDAFSKDITKNIV* |
| *Ii*UGT74D1 | MGEESKAKVLVFSFPIQGHINPLLQFSKRLISKNVTVTFLTTSSTHNTIIRRAVAGGATALPLSFVPLDDGFEEGHPATDSSPEYFAKFEQNVSRSLSQLISSMEPKPNAVVYDSCSPWILDVCRKYPGVAAASFFTQCSTVNAIYVHFLRGEFKEFQDDVVLPAMPPLKGRDLPVFLYDNNLCRPLFELISSQFVNVDHIDFFLVNSFDELEVEVLEWMKKQWPVKNIGPMIPSMYLDKRLADDKDYGISLFNAQVNECLDWLDSKPPGSVMYVSFGSLAVLKDDQMKEVAAGLKQTGHNFLWVVRETESQKLPSSYIEEIGEKGLIVNWSPQLQVLAHKSIGCFMTHCGWNSTLEALSLGVALIGMPAYSDQPTNAKFIEDVWKVGVRVKADKDGFVTKEEIVRCVGEVMEETSEKGKEIRKNALRLMEFAKEALSQGGNSDKNIDEFVAKIAR* |
| *Ii*UGT74C1 | MGEANGHVLFFPYPLQGHINPMIQLAKRLTKKGLTVSLIIASNNHREPYTSDDYPITVHTIHDGFLPHEHPLAKIKDPQRFNVSTSRSLTDFISRMKLSGSHPKALIYDPFMPFALDVAKDLGVYVVAYFTQPWLASLIYYHINEGTYDVPDDRHENPMLASFPAFPLLSQHDLPSFACEKGSYPLIYEVVVSQFSNLRRADLILCNTFDQLEPKVVKWMNDQWPVKNIGPVVPSKFLDNRLPEDKDYELGDFKTEPDDSVLRWLANKPAKSVVYVAFGTLVALSEEQMKETAMAIRQTGYNFLWSVRDSERNKLPSGFVEEALEKDCGLVAKWVPQLEVLSQDSIGCFVTHCGWNSTLEALCLGVPLVGMPQWTDQPTNAKFIEDVWKIGVRVKTDEEGFVSKEEIARCIVEVMEGEKGKVMRKNVEKLKVLAREAISEGGTSDKNIDDFVSILT* |
| *Ii*UGT89A2d | MASEESSRPHMMVFPFPAQGHLLPLLDLTHQLCLRGVNVSVVVTPGNLQYLSSLLSAHPSSVTSVVFPFPQQPSLPPGVENVKDLGNSGNLPIMASLRQLRDPITHWFRSHQNPPVALISDFFLGWTQDLCDQIGVPRFAFFSSGAFLASVLQFCFENIETLRSTTTDSIPFSDLPRVPNFKEEHLPSVVRRSLRSPSSDLETIKDENLMNFLSYGCVFNTAECLEAEYMEYVKQRVGHDRVFGVGPLSLLGLDPVKPDSGSVSENPLLSWLDGCPERSVLYICFGSQKALTKDQCDALSLGLEKSMTRFIWVVKKDPVPEGFEDRVAGRGMVVRGWAPQLELLRHVAVGGFLSHCGWNSVLEGLTSGTMILGWPMEADQFVNARLLVEDLGVAVQVCQGDGTVPDPDELGRVIGETMGELGRRVGARTEEIRRKVVGAVTDAKGSSFADLERLVKEFTLL* |
| *Ii*UGT86A1 | MERTKSRNPHAMMIPYPLQGHVIPFVHLAIKLASHGFTITFVNTESIHHHISTASQGDAGDIFSAARSSGNLDIRYTTVSDGFPLAFDRSLNHDQFFEGILHVFSAHVDDLITKISHRDGDPPVTCLIADTFFVWSSMICDKHNLVNVSFWTEPALVLNLYYHMDLLISNGHFKSLDNREDVIDYVPGVKAINPKDLMSYLQVSDKDVDTNTVVYRILFRAFKDVKKADFVLCNTVQELEPDSLSALQAKQPVYAIGPVFSTQSVVPTSLWAESDCTEWLKGRPTGSVLYVSFGSYAHVGKKEIVEIAHGLLLSGLSFIWVLRPDIVGSDVPDFLPTGFMDRAKGRGLVVQWCCQMEVISNRAIGGFLTHCGWNSILESVWCGLPLLCYPLLTDQFTNRKLVVDDWRIGINLSEKKMITREEVSVNVKLLMNGETSSELRNNVEKVKRHLKDAVTTVGSSETNFNSFVGHVRDSIETKLCNINGLEISRSD* |
| *Ii*UGT91A1a | MTNVKDNSGAKLHVAMLPWLAFGHMVPHFELSKLMAQKGHTVSFISTPRNIHRLLPRLPENLSSAINFVKLHLSAGVDSKLPEDGEATTDVPFDLIPYLKIAFDGLKNPVTEFLESSKPDWILVDFTANWLPPIARRLGIKTAFFSAFNGASLGVLKPPGFEEYRTSPADFLTPPKWVPFETPVAFKLFESWYMFNGFMTDTTEGNVPDIHRFAGVMDGCDVIAVRSCYEYEAEWLGLLQDLHRKPVIPVGVLPPKPEEKYEDTDTWLSIKRWLDSRESKSVVYVGFGSEAKPSQTELNAIALGLELSGLPFFWVLKTGRGPWDTEPVELPEGFEERTKERGMVWRGWVEQLRTLSHDSIGLVLTHPGWGSIIEAVRFSKPMAVLVFVYDQGLNARVIEEKKIGYMIPRDDTEGFFTKEDVAKSLRLVMEEEEGKVYRENVKEMKGVFGDMERQDRYVDAFLDYLVANR* |
| *Ii*UGT91A1b | MTNLKDNSGTKLHVAMFPWLAFGHMVPYFELSKLMAQKGHTVSFISTPRNIDRLLPRLPENLSSAINFVKLPLLGGVDSKLPEDGEATTDVPFDLIPYLKIAFDGLKIPLTEFLESSKPDWILQDFAAYWLPPISRRLGIKTGFFSAFNGATLGILKPPGFEEYRTSPADFLTPPKWVPFKTPVAFKLFECRYIFKGFMAETTEGNVPDIHRAAGVIDGCDVIAVRSCYEYEAEWLGLMQDLHRKRVIPVGVLPPKLEEKYEDTDTWLSIKDAIALGLELSGLPFFWVLKTRRGQWDTEPVELPEGFEERTKGRGMVWRGWVEQLRTLSHDSIGLVLTHPGWGTIIEAVRFSKPMAMLVFVYDQGLNARVIEEKKIGYMIPRDETEGVFTKEDVAKSLRLVMEEEEGKVYRENVKEMKGVFGDMDRQDRYVDSFLDYLVANR* |
| *Ii*UGT84B2a | MGSSEGQETHVLLVALPYQGHLNPMLKFAKHLSRPNLRFTLATTDHARDLLSTTTTAAEEHRSSVKLAFFPDGLPKDDPRVDVSVIMSLRNVGAKNLSKIVESKRFSCIVTVPFAPWVPGVAAAYNIPCALLWVEACGTFSVCYRYYMKTNTFPDDLEDLSQTVELPALPLLEVGDLPSFLLPSAGSHVNDLMVEFVDCLENVKWVLVNSFYELESEVINSMSDLKPIIPIGPLVSPFLLGADEDKTLDDEKNLDLWRADGDCLEWLDKQARSSVVYISFGSLLKSSENQVESIATALRNRGVSFLWVIRPKERAEAVYVLQEMVKEGQGVVIEWGPQERILSHVAISCFVTHCGWNSTMETVAGGVPVVAYPSSIDQPLDARLLVDVFGIGVRMRNDAADGELKVSEVERCIEAVTEGPSAEDMRRRATELKYAARSALAPGGSSARNLDSFISEITIT* |
| *Ii*UGT84B2b | MGSSEGPETHVLLVALAIQGHLNPMLKFAKHLSRPNLRFTLATTEQARDLLSTTTPAEEEHRSPVDLAFFSDGLPKDDPRGHDALIVSLRNVGAKNLSKIVESKRFSCIVTVPFAPWVPGVAAAYNIPCALLWVQACGAFSVHYRYYMKTNTFPNDLEDLSQTVVLPALPLLEVGDFPSFLLPSAGSHVNNLMADFVDCLKNVKWVLVNSFYELESEIIDSMSDLKPIIPIGPLVSPFLLGADEDKTLDDENNLDLWRADGDCMEWLDKQDRSSVVYISFGSLLKSSENQVESIATALRNKGVSFLWVIRPKERAQAVDVLQEMVKEGQGVVIEWGPQERILSHVAISCFVTHCGWNSTMETVATGVPVVAYPSWIDQPLDARLLVDVFGMGVRMRNDAVDGELKVAEVERCIEAVTEGPSAEDMRRRATELKHAARSALAPGGSSARNLDSFIGSITIT* |
| *Ii*UGT84B2c | MGSFECQETHVLMVTLPMQGHINPMLKFAKQLSRLNLRFTLATTEQARDLLSTTTTAAEEHRIPVDLAFISDGLPKDDPRADGTLLESLRNVGGENLSQIIGQKRFSCIISSPFTPWVPAVASAHDIPCGILWIQACGAFSVYYRYYMKTNTFPDDLEDLSQTVELPALPLLEVRDLPSFLLPSAGSHFNKLMADFASCLSDVKWFFVNSFYELESEIIESMSDLKPIIPIGPLVSPFLLGADEGKTLDGKNLDMWKSDDYCMEWLDKQARSSVVYISFGSLLKSSENQVESIATALRNRGVSFLWVVRPKERAQTVDVLQEMVKEGQGVVIEWGPQEMILSHVAVSCFVTHCGWNSTIETVTAGVPVVAYPSWTDQPIDARLLVDVFGMGVRMRNDAVDGELKVTEVERCIEAVTEGPDAADMRRRATELKHAARLAMAPGGSSAQNLDSFISDITIT* |
| *Ii*UGT81A1a | MLREIEKGLMKYQPDIIISVHPLMQHVPLRVLRSKGLLKKIVFTTVVTDLSTCHPTWFHKLVTRCYCPSTELAKRALKAGLRTSQIKVYGLPVRPSFVKPVRPKVELRRELGMDENLPAVLLMGGGEGMGPIEATARALGDALYDENLREPIGQILVICGRNKKLVSKLSSLDWKIPVQVKGFITKMDECMGACDCIITKAGPGTIAEAMIRGLPIILNDYIAGQEAGNVPYVVENGCGKFSKSPKEISKIVADWFGSGSEELEIMSQNALRLARPDAVFKIVHDLHELVRERNGLLSQLACTA* |
| *Ii*UGT81A1b | MLEAFHGHGNLEVANVQTAELGGSSKARKSLVFNTEEAPAEETEASLDMANLSVLSEMEAIDDLLEEDPKGTDVAGIEEVVMMDIVPKADKVEESTVGLHMGDEGLEEGRFINVPIVELGVADEEGDQRDDKVDSVIPPSGVAIADPKLGQRRKLTRIIPGTFGGSTKKRIVQTLVSPRKKGLQRNAIKGGEVSASEGDTVPLNTKKAVGTLVSYTICVEDVDDAEIEKGLMKYQPDIIISVHPLMQHVPLRVLRSKGLLKKIVFTTVVTDLSTCHPTWFHKLVTRCYCPSTELAKRALKAGLRTSQIKVYGLPVRPSFVKPVRPRVELRRELGMDENLPAVLLMGGGEGMGPIEATARALGDALYDENLREPIGQILVICGRNKKLVSKLSSLDWKIPVQVKGFITKMEECMGACDCIITKAGPGTIAEAMIRGLPIILNDYIAGQEAGNVPYVVENGCGKFSKSPKEISKIVADWFGSGSEELEIMSQNALRLARPDAVFKIVHDLHELVRERNGLLSQLACTA* |
| *Ii*UGT71C3a | MLKYLPERHRRIASELELSEEHHPIPGFVSSVPSKVLPSGQFVRESYEAWIEIAQKFPKAKGILVNSFTCLEQNAFDYFACLPENFPPVYPVGPVLSLEDRPSPDLDTSDQCRVMTWLDDQPESSVVYLCFGSFGVLGEPQIEEIARALEISSHRFLWSIRTEKATPYDLLPEGFMDRTVSKGLVCGWAPQVEVLAHKAVGGFVSHCGWNSVLESLWFDVPIATWPLYAEQQLNAYTMVKELGLSVELRLDYVSAKKVIVKADEIAGAIRSLMDGEDTPRRRVKKMAEAARMALMEGGSSFVAVKRFIDDLVGEDF* |
| *Ii*UGT91C1a | MEKGEPALNVVMFPWLAMGHLIPFLRFSKLLAQKGHKVSFISTPRNIERLPRLPSNLSSSITFVSFPLPPFPGSTPGSECSMDVPSNKQQSLKCAFDLLQLPLAEFLRQSSPDWVIYDYASHWLPPVAADLGISKAFFCISNAASLCFLGPPSSLIEDPRSKPEDFTVVPPWIPFRSNIAYRYHEINWYGEKIEEDPTGVSDVVRFGNSISKGDAVFLHSSQEFEPEYLGLLRDLYQKPVFPTGFLPTCTEAEVEGDTTWVDIKKWLDEQRVNSVVYVALGTEASLRPRELAELAHGLEKSEVPFIWVLRNESHIPDGFEKRVEGRGMVHVGWVPQVKILSHDSVGGFLTHCGWNSLVEGLGLGRVPILFPVMNEQGLNTRLLEGKGLGVTIPRDEKEGSFDSESVAHSVRLAMVDDAGELTRAKAKLMKGLFGNMNENFRNMDELVGYMTSKTQSHC* |
| *Ii*UGT71C3b | MEKQEIIFVPYPTPGHLLVTIELAKSLFKRDQRIHTITILHWTLPLAPHADLFAKSLIASEPRILSSRDGSDPVRFAGLVLDFFCVPLIEVGDEFNLPSYIFLTCNAGFMGMLKYLPERHRKIASELELSDEHHPIPGFVSSVPSKVLPSGQFVRESYEAWIEIAQKFPKAKGILVNSFTCLEQNAFDYFACLPENFPPVYPVGPVLSLEDRPSPDLDTSDQCRVMTWLDDQPESSVVYLCFGSFGVLGEPQIEEIARALEISSHRFLWSIRTEKATPYDLLPEGFMDRTVSKGIVCGWAPQVEVLGHKAVGGFVSHCGWNSVLESLWFGVPIATWPLYAEQQLNAYTMVKELGLAVELRLDYVSAKRVIVEAEEIVGAIRFLMDGEDTPRRRVKKMAEAARMALMDGGSLFVAVKRFIDDLVGEDF* |
| *Ii*UGT88A1a | MENQEAIVLYPAPPIGHLVSMVELGKLILSRNQSLSIHIILAPPPYQPESTATYISSLTSSFPSITFHHLPAVTPYSSTSTSRHHHETLLLEIISFSNPNVHRTLLSLSQNFNLRAMIIDFFCTAVLDVTADFTFPVYYFLTSGAAYLAASLYLPTIHETTAGKNLRDVETLHIPGVPPVKGSDMPKAVLDRDDEIYDAFIMFCERISTSSGIIVNTFDALENRAIKAITEDLCFRNLYPIGPLIVQGRTGENDNADSCLDWLDLQPEQSVVFLCFGSLGLFSEEQVKEIAIGLERSEQRFMWVVRNPPGLEHRTEPDLNSLLPEGFLNRTGNRGMVIKSWAPQVPVLNHKAIGGFVTHCGWNSILESVCAGVPMVAWPLYAEQRFNRVVIVEEIKIAVSMNESDTGFVSSMEVEKRVREIIQEGPVREKTNAMKNAAELALTETGSSHAALTVLLQSWSSK* |
| *Ii*UGT88A1b | MENQEAIVLYPAPPIGHLVSMVELGKLILSRNQSLSIHIILAPPPYQPESTATYISSLTFHHLPAVTPYSSTSTSRHHHETLLLEIISFSNANVHQTLLSLSQNFNLRAMIIDFFCTAVLDVTADFTFPVYYFLTSGAAYLAASLYLPTIHETTAGKNLRDVETLHIPGAPPVKGSDMPKAMLDRDDEIYDAFIMFCERISTSSGIIVNTFDALENRAIKAITEDLCFRNLYPIGPLIVQGRTGENDNADSCLDWLDLQPEQSVVFLCFGSLGLFSEEQVKEIAIGLERSEQRFMWVVRNPPGLEHRTEPDLNSLLPEGFLNRTGNRGMVIKSWAPQVPVLNHKAIGGFVTHCGWNSILESVCAGVPMVAWPLYAEQRFNRVVIVEEIKIAVSMNESDTGFVSSMEVEKRVREIIQEGPVREKTKAMKNAAELALTETGSSHAALTVLLQSWSSK* |
| *Ii*UGT76E12a | MEEKAARKRIVLVPIPAQGHITPMMQLAKALHLKGFSITVAQTKFNYFSPPDDLTDYQFITIQESLPESDLKNLGQIRLALKLNKECQVSFKDCLGQLLLQQGDEIACVVYDEFMYFAEAVAKELKLPSVIFSTTSAAAFLCRSVFDKVCSDNESKEKQDELVPEFHPLRYKDFPDSRWSSVEDIEELYRNAVDKRTASSVIINTASCLESSSLSCLQQELGVPVFPIGPLHMVASAPTSLLEENTSCIEWLNKKKQNSVIFISLGSLALMEVNEVMEMVSGLAATNQNFLWVIRPGSVRGSEWIESLPEEFSKMVSDRGYIVKWAPQKEVLAHPAVGGFWSHCGWNSTLESIGEGVPVICRPFTGDQKVNARYLECVWRIGIQVEGELDRGTVERALKRLMVSEEGEEMRKRAISLKEKLRASVRNGGSSHNSLEEFVHVMKTM* |
| *Ii*UGT76E12b | MQKAARKRVLLVPAPAQGHISPMMQLAKTLHLKGFTITVAQTKFNYFSPSEDFADFQFLTIPESLPETDFESLRPVLFLLKLNKECHVSFKEWLGQLLLQQGNEISCVIYDEFMYFAKAAAEEFKLPNVIFSTTNATTFVCRSVFEKLHANNFLAPLKDSKEQQEELVPGLHPLRYKDIPTSTFSSLESILELYRNAVDKQTASSVIINTVSCLESSSLSSLQQQLEIPVYPIGPLHMVASAPTSLLEENKSCIEWLNKQKQNSVIFVSLGSIALTEINEVMETASGLDSSNQHFLWVIRPGSVLGSEWIESFPEEFSKMVSGRGYIVKWAPQKEVLAHPAVGGFWSHCGWNSTLESIGEGVPMICRPFSADQKVNARYLECVWRIGIQAEGELDRGVVERAVKRLMVGEEGEEMRKRAIALKERLRASVRDGGSSHNSLDELVHFMKSL* |
| *Ii*UGT76E5 | MEKKAEKKKIVLVPFQAQGHVTPYMQLGKALSLKGFSITVVQGEFHQLSRTSGFQFVSIPFLPISQIKTLGPVDFLIKLNKTSEASFKDCISQLLQQQDNDIACIIYDELMYFSGVAAKELKIPSVIFSTVSATSQVCGCVLSRLNAEKFLIDMEDPEIQDKVVEDLHPLTYKDLPTSGMGPLDRFLEICREIFNRGTASGIIINTASCLESSSLSWLQQELKIPVFSLGPLHVTVSTNSSLLEEDMSCIEWLNKQKPRSVIYISLGSKADMETKEVLEMAWGLSNSNQAFLWVIRPGSVPDSESLLEEVSKIVSEKGYIVKWAPQSEVLGHPAVGGYWSHCGWNSTLESIVGGVPTICRPFNGEQKLNAKYIESVWRTGIQLQGEVERGGVERAVKRLIVDEEGAGMRERALVLKEKLEASVRCGGSSYEALDELVKYLETECYYRSLYC* |
| *Ii*UGT72E1a | MSIEEMQNTKPHAAMLTSPGMGHVIPVIQLGKRLAGFHGFHVTIFVLEADAASAQSQFLNSPGCDATTLVDVIGLPSPDISGLVEPSASFGTKLLTMMREAVPSIRSKIAEMQHKPTALIVDLLSLDALRLGGEFNMLTYLFIASNARFVALMMYFPTLDRDVEEEHIIKKKPLAIPGCEPLRFEDTFEIFLDPSSQMYQECVPLGLVYATVDGIIVNTWDDMEPKTLKSLQDPKLLGRIARVPIYPIGPLCRPVDPSKTNHPVLDWLNKQPDESVLYISFGSGGSLSAKQLTELAWGLELSQQRFVWVVRPPVDGSACSAYFSVNTGQVRDGTPDYLPEEFVSRTLERGLVVPSWAPQAEILAHTAVGGFLTHCGWNSILESVVIGVPMIAWPLFAEQMTNATLLNEELGIAVRSRRLPSEGLILREEIEALVRRIMVDEEGCVMRKKVKKLSDTAEKSLSCEGTLSRVAEECERRLEHDRSMARGA* |
| *Ii*UGT72E1b | MKITRPHAVMFASPGMGHVIPVIELGKRLVGSHGFQVTIFVLEADAASAQSQFLNSTGCDATLIDVICLPTPDISGLVDPSAFFAIKLLTMMRETIPTIRSKIEEMQHKPTALIVDLFGLDALRLGGEFNMLTYVFIASNARFVALTLYFPTLEKDAEEEHIIKKKPLAMPGCEPVRFEDTLEPFLDPTDQIYRIFVPFGLVYPTADGIIVNTWDDMEPKTLKSLQDPKLLGRIARVPVYPIGPLSRPVDPSKTNHPVLDWLNKQPEESVLYISFGSGGSLSAKQLTELAWGLELSQQRFVWVVRPPVDSSACSEYLSANSGEVQDGTPDYLPKEFISRTQERGLVVPSWAPQAEILAHQAVGGFLTHCGWNSVLESVVSGVPMITWPLFADQKMNATLLNEELGVAIRSRKLPSEEVTLRVEIESLVRRLMVEDEGREMREKVKKLRDTAEMSLRCDGGSSHESLSRVANECHRLLERARMARGA* |
| *Ii*UGT73D1 | MLCVLYLKIFDKPKKMETKMVSEAKRLHFILIPLMAQGHLIPMVDISKILARQGNIVTIVSTPQNASRFAKTVERARSESGVEINVVIFPIAYKEFDLPENCESLDTLPSKDLLRKFYDAVDKLQEPLERFLDQHETPPSCIISDKCLFWTSRTAKKFKIPRIVFHGMCCFSLLSSHNVHLHSPHLLVSSDSEPFSIPGMPHKIEIARDQLPGAFKKLANMDDVREKMREAESEAFGVIVNSFQELEPGYAEAYSEAIKKKVWFVGPVSLCNDRMMDLFDRGNNGNIAISEAECLNFLDSMRPRSVLYVCLGSLCRLIPSQLIELGLGLEESGKPFIWVIKTEERHMSELNEWLKCEKFEERVRGRGIVIKGWSPQAMILSHGSTGGFLTHCGWNSTIEGLCFGVPMITWPLFAEQFLNEKLVVEVLKVGIRVGVEIPVRWGDEERLGVLVRKHSVVKAIKLLMDEDCKRGTEDEDDSEFLRRRTRIQELAVMARKAVEGGGSSTTNVLILIQDILEQLSLR* |
| *Ii*UGT76F2 | MDLSGNFQVPFFPIGPFHKHRDEIPLKTKKKENYETTNWLDKHDPKSVVYASFGSLAAIEEKEFLDIAWGLRNSKQPFLWVVRPGLVRGTEWLESLPCGFMENIGHKGKIVKWTNQLEVLAHPAVGLFWTHCGWNSTLESICEGVPMICTPCFGDQLVNAKYIVDIWQVGMMLERGMMDKEKIEQTVRSAMIDKGDKMRDKCLPFKERADNCLQKNGSSSNYLDKLVNDVLSFDSYVITS* |
| *Ii*UGT85A4 | MLKLIDSTVNNCLSPFQELLIRLNSGSDVPPVSCIVSDASMSFTIDAAEELEIPVVLLWTNSATALILYLHYRKLIEKKIIPLKGMYVSLEIEFYHLNFITKFKISADERHLETEIDWIPSMKNIRLKDFPDFVSVTDHQDLMLNFILHVTGRSKRASAIMINTFGNLEGNVVSSLRSLLHPRIYPIGPLPVFENREIDRDSEIGRMRLNLWEEETESLDWLDTKAKNTVLYVNFGSLTVLTRELLLEFAWGLAGSGKEFLWVVRSGTVDGDASTLPSEFLSETADRGMLITGWCPQEKVLSHPAIGGFLTHCGWNSTLESMFAGVPMICWPFFADQLTNRKFCCEEWGMGIEIGDGVKREKVKAVVSELIDGVKGKKIREKVMEWRLHGGRSFDATVWFVVREF* |
| *Ii*UGT72D1 | MEQPHALLVASPGLGHLIPILELGNRLSSVLNIHVTVLAVPSGSSSSPTETEAIRAAVARGTCEIAELPSVDIEHLVEPDATVATRIFEKMRATRPAVQDAVKAMNRKPTVMIVDFFATGLMSVADDVGVTAKYVYVPSHAWFLAVMVYLPVLDKVVEGEYIDIKEPMKIPGCRSVGPDELMDTMFDRSDRQYRECVRCGEEIPMSDGILVNTWEELQGKTLAALREDGELSRVMKVPVYAIGPFVRSNGPIEKPKSIFEWLDKQRDRSVVYVCLGSGGTLSLEQTMELAWGLELSGQSFLWVLRRPTSYLEARSSDDDQVSAGLPEGFLDRTRGVGLVVTQWAPQVEILSHGSIGGFLSHCGWSSVLESLTKGVPIVAWPLYAEQWMNATLLTEEIGVAVRTSELPSKKVIGREEVASLVRKIVAEEDEEGRKVRAKAEEVKATSEAAWAQGGSSHGSLLKWAKRCRLVCDSQII* |
| *Ii*UGT76E4 | MQEKVVENLYPLRYKDLLPSGFGPVEPVLKIRREVVNKRTASAVIFNTTSCLESSSVSWLQQELGIQVYALGPLHITASTPSSLLEEDKGCIEWLNKQEPRPVIYVSVGTVAQIETKEVLEMAWGLCNSNQPFLWVIRPGSILGSDGIGTLPNEVSTMVSERGYILKRAPQIEVLRHPAVGGFWSHCGWNSTLESIVEGVPLICRPFQSEQKVNAAYLVSVWEIGIQLEGEVERGKVQRAVKKLLVDEEGAGMRERAFVLKEKLKASVRAGGSSYNALDELVSYLKTK* |
| *Ii*UGT90A1 | MAMSSHHVVLFPYMSKGHTIPLLQFARLLLRHRREQTTISVTVFTTPKNQPFVSDFLYYTPEIKVISLPFPEKISGIPPGVESTEKLPSMSLYVPFTRATKLLQPFFEESLKNLPEVSFMVSDGFLWWTSESAAKFKIPRLVFFGMNSYASAVPRSVFNHKLFTEPEIESDTEPITVPDFPWIRVKKCDINHGTLSGPEAELFMDQMMSTTTSQGFLVNSFYELESTFVDYNNNSEGPKSWCVGPLCLADPPKQERTAKPAWIHWLDRKRDQGRPVLYVAFGTQAEISNKQLNEIALGLEDSKVNFLWVTRKDVEEIVGSGFKDRIRETGMIVRDWVDQWEILSHDSVKGFLSHCGWNSAQESICVGVPLLAWPMMAEQPLNAKMVVEELKVGVRVETQDESVQGFVTREELSRKIRELMEGETGKAARKNVKEYSKMAKAALVQGTGSSWKNLNLLLEKLCKSREANDANREIADLC* |
| *Ii*UGT73B5a | MNSEVSERIHVCFFPFMAQGHMIPILDMAKLFSSRGAKSTIITTPSNSKILEKPIEAFKNQNPDLEIGIKIFDFPCVELGLPEGCENVDFITSYQKPDAGDLFLKLLFSTKHMKQQLESFIETTRPSCLVADMFFPWATQSAEKFGVPRLVFHGTSFFSLCCSYNMRIHKPHKKVATSATPFVIPGLPGDIVITAEQANVADEETPMGKFMKEVRESESISFGVLVNSFYELEPTYADFYRSFVAKRAWHIGPLSLCNRDLVEKAERGKKASIDEQECLKWLDSKTTGSVVYMSFGSGTNFTNEQLLEIAAGLEGSGQNFIWVVRKNENKGEKEEWLPEGFEERTKGKGLIIRGWAPQVLILDHKAVGGFVTHCGWNSAMEGIAAGLPMVTWPMGAEQFYNEKLLTKVLKTGVNVGATELVKKGKLISREEVEKAVREVIVGEEAEERRKRAKKLGEMAKAAVEEGGSSYNDLNKFMEEVNGRN* |
| *Ii*UGT73B5b | MNSEVSERIHVCFFPFMAQGHMIPILDMAKLFSSRGAKSTIITTPSNSKILEKPIEAFKNQNPDLEIGIKIFDFPCVELGLPEGCENVDFITSYQKPDAGDLFLKLLFSTKHMKQQLESFIETTRPSCLVADMFFPWATQSAEKFGVPRLVFHGTSFFSLCCSYNMRIHKPHKKVATSATPFVIPGLPGDIVITAEQANVADEETPMGKFMKEVRESESISFGVLVNSFYELEPTYADFYRSFVAKRAWHIGPLSLCNRDLVEKAERGKKASIDEQECLKWLDSKTTGSVVYMSFGSGTNFTNEQLLEIAAGLEGSGQNFIWVVRKNENKGEKEEWLPEGFEERTKGKGLIIRGWAPQVLILDHKAVGGFVTHCGWNSAMEGIAAGLPMVTWPMGAEQFYNEKLLTKVLKTGVNVGATELVKKGKLISREEVEKAVREVIVGEEAEERRKRAKKLGEMAKAAVEEGGSSYNDLNKFMEEVNGRN* |
| *Ii*UGT81A4 | MMKVASPRAQAGSITEKVFRRVYSNFSFSTVDDDYNHDRRRSRSGDYGKESLRKRGFEEKEDVMEMEQMGAERIKTVLILMSDTGGGHRASAEAIRDAFNIEFGDDYRIIIKDVWKEYTGWPLNDMERQYKFMVKHVGLWSVAFHGTSPKWIHRSYLSALAAYYAKEIETGLMEYKPDIIISVHPLMQHIPLWVMKRQGLQKKVIFVTVITDLNTCHRTWFHHGVSRCYCPSKEVAKRALVDGLGDSQIRVFGLPVRPSFPRTIIYKDELRKELGIDLNLPAVLLMGGGEGMGPVQKTAQALGNSLYDSKEKYPIGQLIVICGRNKILASTLASQEWKIPIKIRGFETQMEKWMGACDCIITKAGPGTIAEALICGLPIILNDYIPGQEKGNVPYVVDNGAGIFTRSSKETAKIVADWFSSNKNELYKMSENALKLAQPEAVFDIVKDIHHLSQQQQQHIQPFNDFS* |
| *Ii*UGT81A3 | MATSVVSITEKVLERVYGSSKSTLSVAGGGEGEKAQRHTHHHHVRRHSYDDSDDDVCYSDEDESAMELVQIGAERTKNVLILMSDTGGGHRASAEAIRDAFKIQFGDKYRIIVKDVWKEYTGWPLNDMERSYKFMVKHVQLWKVAFHSTSPKWIHSCYLAAIAAYYAKEVEAGLMEYKPEIIISVHPLMQHIPLWVLKWQELQKRVLFVTVITDLNTCHPTWFHPGVNRCYCPSQEVAKRALFDGLDESQVRVFGLPVRPSFARAVLVKDDLRIELGMDRNLRAVLLMGGGEGMGPVKETAKALEDSLYDKENKKPIGQMVVICGRNKKLASSLEAVEWKIPVKVRGFDTQMEKWMGACDCIITKAGPGTIAESLIRSLPIILNDYIPGQEKGNVPYVVENGAGVFTRSPKETARIVGEWFSTKTDELEQTSDNARKLAQPEAVFDIVKDIDELSEQRGPLAKVAYTLTSSFASLV* |
| *Ii*UGT78D2a | MANTSEPATDSHVAVLAFPFGTHAAPLLAVTRRLASAAPTTVFSFFNTAQSNSPLFLSSDDAGLPPNIRVHDVADGVPEGYVFAGRPQEAMELFLVAAQENFRKAIAAAGRKVTCMLTDAFFWFAADMAAEMSATWVAFWTAGPNSLSAHLYTDLIRESVGVEEVDGRMEQTLGFISGMEKMRVKDTPEGVVYGNLDSVFSNTLHQMGLALPRATAVFINSFEELDHTLTSNLKSEFKGYLNIGPLALLSSTSQTDTLVQDHHGCLAWMEKQSPASVAYISFGTVMTPPPGELVAIAEGLESSKVPFVWSLKEKNMVHLPKGFLDRTREQGIVVPWAPQVELLKHEATGVFVTHCGWNSVLESVSGGVPMICRPFFGDQRLNGRAVEVVWEIGMTIINGVFTKDGFEKCLHRILVEDDGKKMKGHAKKLKEQAYQAVSAKGSSVENFKGLLDAVVGILN* |
| *Ii*UGT78D2b | MANTSEPATDSHVAVLAFPFGTHAAPLLAVTRRLASAAPTTVFSFFNTAQSNSPLFLSSDDAGLPPNIRVHDVADGVPEGYVFAGRPQEAMELFLVAAQENFRKAIAAAGRKVTCMLTDAFFWFAADMAAEMSATWVAFWTAGPNSLSAHLYTDLIRESVGVEEVDGRMEQTLGFISGMEKMRVKDTPEGVVYGNLDSVFSNTLHQMGLALPRATAVFINSFEELDHTLTSNLKSEFKGYLNIGPLALLSSTSQTDTLVQDHHGCLAWMEKQSPASVAYISFGTVMTPPPGELVAIAEGLESSKVPFVWSLKEKNMVHLPKGFLDRTREQGIVVPWAPQVELLKHEATGVFVTHCGWNSVLESVSGGVPMICRPFFGDQRLNGRAVEVVWEIGMTIINGVFTKDGFEKCLHRILVEDDGKKMKGHAKKLKEQAYQAVSAKGSSVENFKGLLDAVVGILN* |
| *Ii*UGT90A4 | MAVSSSPRHVVLFPYMSKGHTIPLLQFARLLLRQRRVIPSSDDAEEPTISVTVFTTPKNQPFVSNFLSDDVTNTITSSIKIISLPFPENISGIPPGVESTDKLPSMSLYKHSRIFHKLLSWSPMASSGGRWNPPLNSRSRDSSFFGMNSYASASPLLRVRTPTLTKPEIVKSDTEPVTVPDFPWIRVKKCEFFPAVTEPDPSGPEFELFVDQIASTEKSRGVIVNSFYELESTFVEFRLGVEGEPKPWCVGPLCLVNPPKLESDKPSWIHWLDQKREERRPVLYLAFGTQAEISNEQLKELALGLEDSKVCFLWVTRKDVEELTGEIGFEKRVSERGMIVRDWVDQWEILLHESVKGFLSHCGWNSAQESICAGIPLLAWPMMAEQPLNAKLVVEELKIGVRIETEDGTIKGFVSREELSRKVKELMEGEMGKTARENVKEYAEMAKKALAQGTGSSWKNLNSLLQLYKSRETNDVNKLSKDD* |
| *Ii*UGT92A1 | MADAKHRNLNIVMFPFMAQGHMIPFVSLALRLEKMMMRKNRDDNLHLKPTISLVNTPLNIPKLRSKLPPQSSIRLIELPFTSSDHGLPHDAENLDSLPYSLVISLLQASRSLRGPFRDLMTKFLKEGDDVLVIGDFFLGWIGKVCKEMGVFSVIFSASGAFGLGCYRSVWLNLPHKETKQDQFLLHDFPEAGEIERTQLNFFLLEADGSDDWSVFMKKNLPGWSDFDGFLFNTVEEIDQIGLSYFRRITGGKPVWPVGPVLLSSWEKDAGWRSAEEGVKAWLDSKPDHSVVYVCFGSMNSISQAQMLELAKALESSEKNFIWVVRPPTGVEVKREFDLKEYLPEGFEERIKISERGVIVKKWAPQVDILSHKATCVFLSHCGWNSILESLSYGVPLLGWPMAGEQFFNSTLMEKHVGVSVEVARGKRCDIKCDEIVSKIKLVMEEKSEVGRYIRKKAEEVKELVRRAMDDGVNGSSVIGLEEFLGQAMFKKDKY* |
| *Ii*UGT76C3 | MENSNGRRVILFPLPLQGCINPMIQLAKILHSRGFSITVIHTRFNAPKASSHPLFTFLEIPDGLSETEARTDDVTLLLTLLNRSCECPFRDCLTKLLQSADSETEEEEQRISCLIDDSGWIFTQPLAQSINLPRFVLNTYNVSFFRGHFVLPQLRRERYLPQQGFFTYSEQDDPAEEFPPLRKKDLLRILDEETEVLDSYSDMILKTTKASSGIIFVSSCEELDQDSLSQARQDFQVPIFAIGPSHSYFPGSSSSLFTPDETCIQWLDKQEDNSVIYAIPEELMESLKEKGKIVKWAPQQDVLKHLAAGGFLTHNGWNSTVESVCEGIPMICLPFVWDQLLNARFVSEVWKVGLHLEGRIERYEIERAVRRLFLETEGDVIRERMKLLAEEVGRSVKPKGSAYRSLEHLVDHISSF* |
| *Ii*UGT76C2 | MVLRVILFPLPLQGCINPMLQLANILHSRGFSITVVHTRFNAPKASSHPLFTFLQIPDGLSENQINDDPTSNVMSLLAQINCNAESPFRDCLEKLLLQSQDSERISCLIDDCGWLFTQSVAESLNLPRIVLCTFKATFLNAYPILPLLRIREYLPVSDSEAEDSVLEFPPLQKRDLSRVFGEDGEKLDPFLQTVVETTMRSSGLIFMSCEELEKDSLTIAHEIFKVPVFAIGPFHSYFSASSSSLFTQDDTCIPWLDNQEDKSVIYVSLGSVVNITESEFLEIAWGLSNSKHPFLWVVRPGLVLGAQWIERLSEELMRSLEEKGKIVKWSPQQEVLAHRAIGGFLTHNGWNSTLESICEGVPMICLPGGWDQMLNSRFVSDVWRVGIHLEGQIERKEIEKAVRMLMVESGGEKIRERMKVLKDEVGRSVKDGGSSFRSIETLANHMQSL* |
| *Ii*UGT89A2a | MREEKPPESSRSEGSKPHVVVFPFPAQGHLLPLLDLTHQLCLRGVTVSVIVTPGNLPYLSPLISAHPSSVSAVVFPFPPHPALPPGVENVKDVGNFGNFPIMASLRQLRHPIILWFRSHPNPPVALISDFFLGWTHDLGNHIGIPRFAFFSISSFLVSVIQFCFSNVEQIKSKDPVHLLDLPRSPIFKEEHLPSVVRRCLQTPSPDLETIKDFSMNALSYGSVFNSSECLEDEYLEYVKQRMGHDRFFVIGPLCSIGSGLRSVKDPVDPELLSWLDGSPDGSVLYVCFGSQKALTKNQCDALALGLEKSMTRFVWVVKRDPVPDGFEDRVSGRGILVRGWVPQLLVLRHVAVGGFLSHCGWNSVLEGITSGAVILGWPMEADQFVNARLLVDHLGAAVRVCEGAETVPDPDELGRVIAETMGERGRQVAARAEEIRRKTSAEANGSSIAGLQRLVEEFGRV* |
| *Ii*UGT89A2b | MNALSYGSVFNSSECLEDEYLEYVKQRMGHDRFFVIGPLCSIGSGLRSVKDPVDPELLSWLDGSPDGSVLYVCFGSQKALTKNQCDALALGLEKSMTRFVWVVKRDPVPDGFEDRVSGRGILVRGWVPQLLVLRHVAVGGFLSHCGWNSVLEGITSGAVILGWPMEADQFVNARLLVDHLGAAVRVCEGAETVPDPDELGRVIAETMGERGRQVAARAEEIRRKTSAEANGSSIAGLQRLVEEFGRV* |
| *Ii*UGT85A2a | MSFTLDAAEELGIPQVLFWTTSACAFMAYLHFYLFIEKGICPFKDESYLTKEYLDTKIDWIPSMKNLQLKDIPTTIRTTDPDDIVLNFIIRETDRAKRASAIILNTFDDLEHDVIQSMQSIVPPVYSIGPLHRLVDKEIGEDSEIGQMALNLWREETECLDWLSTKSRRSVVYVNFGSVIMISAEQLVELAWGLAATGKEFLWVIRTDLVAGDVEMVPPELLKEAADRMMLAKWCPQEKVLSHPAIGMFLTHNGWNSTLESISAGVPMVCWPFVADQQLNCKFCCDEWEIGVEISGDVKREEVEAVVRELMDGEKGKKMKEKAEEWHHLAEEATEHKRGSSKLNFEMVVNKLLLKE* |
| *Ii*UGT91C1b | MEDKEDEALHVAMFPWLAMGHLLPFLNISKLLAQKGHKVSFISTPRNIQRLPKLPSNLSSSITFVSFPLPSFPNLPPSSESSMDVPYINQQSLKSAFDLLQPPLTEFLRSSSPDWIIYDYSSHWLPPIATELGISKAFFSLFNASVLCFMGPPSSLIEDIRSTPEDFTVVPPWIPFESKIVFRYHEVTRYVQKTEEDVTGVSDSVRFGYSIGGSDAVFVRSSPEFEPEWFGLLRDLYQKPVFPTGFLPPVREDHEDDGDDDATWVRIKKWLDKQRVNSVVYVSLGTEASLPQEELAELALGLEKSQVPFFWVLRKEPTLSEPLIPERVEERGMVHTGWVPQVKILSHESVGGFLTHCGWNSVVEGLGFGRVPIFLPVLNEQGLNTRLLEGEGIGVEVPRNERDGSFDSDSVAESVRLAMIDDAGELKRRKAKLMRGLFGDKDENIRYVDELVDYITSKGSSHV* |
| *Ii*UGT76D1a | MDKIRQKRVLMVPAPFEGHLPSMMNLASFLSSKGFSITIVRTRYNFYDISADFPSFNFFTIDDGLSESNMSYLGLLDFILELNSVCEPLLKEFITLNNDVDFIIHDEFVYFPRKVAEDLNLPKMVFSASSAATSISRCVLMENQDKWLLHAQEARSTLEDIVPMFPPFRYKDLPVTAYGSMERLMLLYRNVSKRDTSSGIIHNSSHCLEDSFTSAAEDRWGVPVYPVGPLYLTDSATPCPSLFKEERDCLAWLEKQETNSVIYISMGSLAMTQKKEFEEMAMGLVQSNQSFLWVVRPGSITGQNSLESLPEQFRQAVSHDGRGFVVKWAPQKEVLRHRAGGRVLEPLWMELDLGEYKQWRANDLQAVFRGPEGKCATYDTEIVEMAVRKLIEGDEGKEMRKTAITLREKIKDSVKIGGSSYNALDSRMLLFLAMSSINADHGMSPSRAGSGMSTSHAGHDKQQ* |
| *Ii*UGT76E6 | MMQLGKALDLKGFSITVVQGHFNQVSSSSHQPSGFHFITIPESLPESELETLGPTDFLMKLNETSLASFKDCIAQLLLQHDNDIDCIVYEDLMYFCGDAAKEFQLTSIIFTTTTAIAKVGCTVISKLNAKKFLIDMEDPEMQDKRSNQKRGASAVIINTVSCLESLSLSWLQRELEIRIYPLGPLHITASAPSYLLEEDKSCIEWLNKQMPRSVISISFGSMAQMDFKEVSEMVYGLSNSKQPFLWVIRPGSILGSDKMESIPKEVSKMLSEKGYIVKWAPQREILAHPALGGFWSHCGWTSTLESIAEGVPMICRPFISEQKLNAKYI* |
| *Ii*UGT79B9 | MGQKFHAFMFPWFAFGHMTPYLHLANKLAEKGHRVTFLLPTKALKQLEHHNLFPNNIVFHPLTIPPVDGLPAGAETASDIPISLGKFLSAAMDLTRDQVEAAVRALRPDLILFDFAYWIPELAKEHRVKSIMHNVVSATSIANGFVPGGALGVPPPGYPSSKVVYRAHDAHALLSFSVYYKRLYHRLTTGLTNCDFISIRTCEEVEDTSKPLEDQWSRWLAGFRQGSVVFCALGSQITLEKDQFQELCLGMELTGLPFLVAVTPPKGAKTIQEALPEGFKERVKDRGVVWGEWVQQPLILAHPSVGCFVSHCGFGSMWESLMNDCQIVLLPYLTDQILNTRLMTEELQVSVEVPREETGWFSKESLSAAITSVMDKDSELGNLVRKNHAKLKEILVSPGLLTGYTDKFEEALQDLVNDTNLE* |
| *Ii*UGT79B1a | MIGKESTKKQTKPMGDFGWNGSSSMSIVMYPWLAFGHMTAFLHLSNKLAEKGHKIIFLLPKKALNQLQPLNLYTNLITFHTITIPQVKGLPPGAETNSDVPFFLTHLLAVAMNQTMPEVETILRTTKPDLVFYDSADWIPEIAKPVGAKTVCFNTVSAASVALTLVPAAEREIIDGNEMSAEELAKPPLGYPSSKVVLRAHEAKTLSFVWRRHEGIGSFFDGKVTAMRNCDAIAIRTCHETEGKFCDYIGNQYNKPVYLTGPVLPVDEPNKPSLEPRWGDWLAKFNPGSVVFCAFGSQPVVDKIEQFQELCLGLESTGLPFLVAIKPPSGVSTVEEALPEGFQERVRGRGVVYGGWIQQPLVLDHPSVGCFVSHCGFGSMWESLMSDCQIVSVPQHGEQILNARLMTEEMEVAVEVEREENGWFSRRSLEDAVKSVMDEGSEVGEKVRKNHEKWRRVFSDSGFADGYISKFEQNLTDLVKS* |
| *Ii*UGT79B1b | MIGKESTKKQTKPMGDFGWNGSSSMSIVMYPWLAFGHMTAFLHLSNKLAEKGHKIIFLLPKKALNQLQPLNLYTNLITFHTITIPQVKGLPPGAETNSDVPFFLTHLLAVAMNQTMPEVETILRTTKPDLVFYDSADWIPEIAKPVGAKTVCFNTVSAASVALTLVPAAEREIIDGNEMSAEELAKPPLGYPSSKVVLRAHEAKTLSFVWRRHEGIGSFFDGKVTAMRNCDAIAIRTCHETEGKFCDYIGNQYNKPVYLTGPVLPVDEPNKPSLEPRWGDWLAKFNPGSVVFCAFGSQPVVDKIEQFQELCLGLESTGLPFLVAIKPPSGVSTVEEALPEGFQERVRGRGVVYGGWIQQPLVLDHPSVGCFVSHCGFGSMWESLMSDCQIVSVPQHGEQILNARLMTEEMEVAVEVEREENGWFSRRSLEDAVKSVMDEGSEVGEKVRKNHEKWRRVFSDSGFADGYISKFEQNLTDLVKS* |
| *Ii*UGT76D1b | MDKIRQKRVLMVPAPFEGHLPSMMNLASFLSSKAFSITIVRTRYNFYDISADFPSFRFFTIDDGLSESNMSSLGLLDFILELNSVCEPLLKEFLTLNNDVDFIIHDEFVYFPRQVAEDLNLPKMVFSASSAATSISRCVLMENQDKWLLHAQEAGSTLEDIVPMFPPFRYRDLPVTAYGSMERLMILYRNVSKRDTSSGIIHNSSHCLENSFTSAAEDRWGVPVYPVGPLYLTDSATPCPSLFTEERNCLAWLEKQETNSVIYISMGSLALTQKKEFEEMAMGLVQSNQSFLWVVRPGSITGQNSLESLPEQFRQAVTHDGRGFVVNWAPQKEVLRHRAVGGFWNHCGWNSTLESISNGVPMICRPYSGDQRVNARLMTHVWQTAFEVEGELNREVVEMAVRKLIEGNEGKEMRKTAITLREKIKDSVKIGGSSYSALERLVDSITSSCVP* |
| *Ii*UGT73B3b | MISSFRMLQIEVLDFPCVQLALPEGCENADFFTSNNNDDRQYMSFNFFLSTRFFKDQLEKLLETMRPDCLIADMFFPWATEAAEKFNVPRLVFHGTGYFSLCSDYCIRLHKPQKRVASSCEPFVIPDLPGNIVMTQEQINDRDEETEMGSLYSSFYELEPEYADFYKSVVAKRAWHIGPLSFVNRGFEEKAERGKKASIDEVQCLKWLDSKKPDSVVYISFGSVAFFKNEQLIEIAAGLEASGTNFIWVVRKNKGDNEEWLPEGMEERTKGRGMIIRGWAPQVLILDHQATGGFVTHCGWNSLLEGVAAGLPMVTWPVGAEQFYNEKLVTQVLRTGVTVGAKRQVRGLGDCISREKVEKAVREVLFGEEAEERRRRAKKLAEMANAAVEEGGSSFNDLNSFIQEFGS* |
| *Ii*UGT84A1d | MEFGTSSPPNPIHVMLVSYVGQGSVSPLLHLGKLIASKGTIVTFVTTEFWGKKMRQANKIVDGELKPVGSGSIRFEFFNDGWAEDDERRGNFALFIPQLEQAGKREVSKLVRRYEGKNEPVSCLINNPFMPWVGHVAEELNIPFAMLWIQSCACFSAFYHYQNGSVPFPTAAEPKLDVKLPCVPVLKYDEIHTFLHPFSPQAGLRKAILGQFETLSKPFCVLISSFDALEQEVINYMSKLCPIKTVGPLFKVAKSVNSDVSGDFCKPSDQCLGWLDSRPKSSIVYISFGTVAYLKQEQMEEMARGVLKSDLKEASDRGKGKIVDWCPQDQVLAHPSVACFVTHCGWNSTMEALTLGVPVVCFPQWGDQVTNAVYLVDVFKTAVRLGRGAADGRIVPGEEVAEKLLEATVGEKAEELRKNALKLKAEAEAAVAPGGN* |
| *Ii*UGT72E3 | MQITKPHAAMFSSPGMGHLIPVIELAKRLSANHGFRVTVFVLEADAASAQSKFLNSTGVDVVNLPSPDISELVDPADHVVTKIGVIMREAVPALRSKIAEMNQKPTALIIDLFGTDALCLAAEFKILTYVLIASNARYLGVAMYYPTLDKHVKEEHTVQRKPLEIPGCEPVRFEDTMDAYLVPDEPLYRDFVRHSLAYPKADGILVNTWDEMEPKSLKSLQDPTLLGRVARLPVYPVGPLCRPVEPSKTGHPVLDWLNEQPDESVLYVSFGSGGSLTAKQLTELAWGLEQSQQRFVWVVRPPVDGSSCCEYFSVNGGGTKESTPEYLPEGFVTRTCDRGLVVPSWAPQAEVLAHRAVGGFLTHCGWNSTLEGVVSGVPMIAWPLFAEQNMNAALLSDELGIAVRADNLKEAVTRLEIEAIVRKVMEEEEGEEMRRKVKKLTGKAEMLLSSDGGGSAHESLCRVTEECERFLERDMDLARGA* |
| *Ii*UGT71B2 | MEESKAAHVALIPSPGMGHLIPLVQFAKRLVERHGFSITFIVVGEGPPSTSQRTVLDSLPSSISSVFLPPADLTDLPPTTRIETRISLTVNRSNPGLRRVFETFAAEGRLPTALFVDLFGTDAFDVAAEFHVPPYIFFPSTANVLSFFLHVPKLDETMSCEFRELTEPVKLPGCVPFSGKDVLDPANDRKNDAYKCLLHNTKRYKEAQGILINTFLELEPNALKALQEPEPGLDKPPVYPIGPLVNIGKQEGSNGTEESECLKWLDKQPLGSVVYVSFGSGGVLTLEQLNELALGLADSEQRFIWVIRSPSQIADASFFNSHSESDPLTFLPPGFLERTKGRGFMIPLWAPQAQILVHPSTGGFLTHCGWNSTLESIVSGVPLIAWPLYAEQKMNAVLLAEDVGVALRAHAGEDGVVRREEVTRVVKGLMEGDEGKVVRNKMKEMKEGASRVLSDDGSSTKALSLLALKWKDHKKELEQNGKP* |
| *At*UGT74E2  (AT3G50740) | MREGSHLIVLPFPGQGHITPMSQFCKRLASKGLKLTLVLVSDKPSPPYKTEHDSITVFPISNGFQEGEEPLQDLDDYMERVETSIKNTLPKLVEDMKLSGNPPRAIVYDSTMPWLLDVAHSYGLSGAVFFTQPWLVTAIYYHVFKGSFSVPSTKYGHSTLASFPSFPMLTANDLPSFLCESSSYPNILRIVVDQLSNIDRVDIVLCNTFDKLEEKLLKWVQSLWPVLNIGPTVPSMYLDKRLSEDKNYGFSLFNAKVAECMEWLNSKEPNSVVYLSFGSLVILKEDQMLELAAGLKQSGRFFLWVVRETETHKLPRNYVEEIGEKGLIVSWSPQLDVLAHKSIGCFLTHCGWNSTLEGLSLGVPMIGMPHWTDQPTNAKFMQDVWKVGVRVKAEGDGFVRREEIMRSVEEVMEGEKGKEIRKNAEKWKVLAQEAVSEGGSSDKSINEFVSMFC* |
| *At*UGT71C1  (AT2G29750) | Mgkqedaelviipfpfsghilatielakrlisqdnprihtitilywglpfipqadtiaflrslvkneprirlvtlpevqdpppmelfvefaesyileyvkkmvpiirealstllssrdesgsvrvaglvldffcvpmidvgnefnlpsyifltcsagflgmmkylperhreiksefnrsfneelnlipgyvnsvptkvlpsglfmketyepwvelaerfpeakgilvnsytalepngfkyfdrcpdnyptiypigpilcsndrpnldsserdriitwlddqpessvvflcfgslknlsatqineiaqaleivdckfiwsfrtnpkeyaspyealphgfmdrvmdqgivcgwapqveilahkavggfvshcgwnsileslgfgvpiatwpmyaeqqlnaftmvkelglalemrldyvsedgdivkadeiagtvrslmdgvdvpkskvkeiaeagkeavdggssflavkrfigdlidgvsisk* |
| *At*UGT71B5  (AT4G15280) | mgqfsivykytipllsltptihsrkvrtqkqkmkielvfiplpgighlrptvklakqligsenrlsitiiiipsrfdagdasaciaslttlsqddrlhyesisvakqpptsdpdpvpaqvyiekqktkvrdavaarivdptrklagfvvdmfcssmidvanefgvpcymvytsnatflgtmlhvqqmydqkkydvselensvtelefpsltrpypvkclphiltskewlplslaqarcfrkmkgilvntvaelephalkmfningddlpqvypvgpvlhlengndddekqseilrwldeqpsksvvflcfgslggfteeqtretavaldrsgqrflwclrhaspniktdrprdytnleevlpegflertldrgkvigwapqvavlekpaiggfvthcgwnsileslwfgvpmvtwplyaeqkvnafemveelglaveirkylkgdlfagemetvtaedierairrvmeqdsdvrnnvkemaekchfalmdggsskaalekfiqdvienmd* |
| *At*UGT89C1  (AT1G06000) | MTTTTTKKPHVLVIPFPQSGHMVPHLDLTHQILLRGATVTVLVTPKNSSYLDALRSLHSPEHFKTLILPFPSHPCIPSGVESLQQLPLEAIVHMFDALSRLHDPLVDFLSRQPPSDLPDAILGSSFLSPWINKVADAFSIKSISFLPINAHSISVMWAQEDRSFFNDLETATTESYGLVINSFYDLEPEFVETVKTRFLNHHRIWTVGPLLPFKAGVDRGGQSSIPPAKVSAWLDSCPEDNSVVYVGFGSQIRLTAEQTAALAAALEKSSVRFIWAVRDAAKKVNSSDNSVEEDVIPAGFEERVKEKGLVIRGWAPQTMILEHRAVGSYLTHLGWGSVLEGMVGGVMLLAWPMQADHFFNTTLIVDKLRAAVRVGENRDSVPDSDKLARILAESAREDLPERVTLMKLREKAMEAIKEGGSSYKNLDELVAEMCL* |
| *At*UGT71C5  (AT1G07240) | MKTAELIFVPLPETGHLLSTIEFGKRLLNLDRRISMITILSMNLPYAPHADASLASLTASEPGIRIISLPEIHDPPPIKLLDTSSETYILDFIHKNIPCLRKTIQDLVSSSSSSGGGSSHVAGLILDFFCVGLIDIGREVNLPSYIFMTSNFGFLGVLQYLPERQRLTPSEFDESSGEEELHIPAFVNRVPAKVLPPGVFDKLSYGSLVKIGERLHEAKGILVNSFTQVEPYAAEHFSQGRDYPHVYPVGPVLNLTGRTNPGLASAQYKEMMKWLDEQPDSSVLFLCFGSMGVFPAPQITEIAHALELIGCRFIWAIRTNMAGDGDPQEPLPEGFVDRTMGRGIVCSWAPQVDILAHKATGGFVSHCGWNSVQESLWYGVPIATWPMYAEQQLNAFEMVKELGLAVEIRLDYVADGDRVTLEIVSADEIATAVRSLMDSDNPVRKKVIEKSSVARKAVGDGGSSTVATCNFIKDILGDHF* |
| *At*UGT74B1  (AT1G24100) | MAETTPKVKGHVVILPYPVQGHLNPMVQFAKRLVSKNVKVTIATTTYTASSITTPSLSVEPISDGFDFIPIGIPGFSVDTYSESFKLNGSETLTLLIEKFKSTDSPIDCLIYDSFLPWGLEVARSMELSAASFFTNNLTVCSVLRKFSNGDFPLPADPNSAPFRIRGLPSLSYDELPSFVGRHWLTHPEHGRVLLNQFPNHENADWLFVNGFEGLEETQDCENGESDAMKATLIGPMIPSAYLDDRMEDDKDYGASLLKPISKECMEWLETKQAQSVAFVSFGSFGILFEKQLAEVAIALQESDLNFLWVIKEAHIAKLPEGFVESTKDRALLVSWCNQLEVLAHESIGCFLTHCGWNSTLEGLSLGVPMVGVPQWSDQMNDAKFVEEVWKVGYRAKEEAGEVIVKSEELVRCLKGVMEGESSVKIRESSKKWKDLAVKAMSEGGSSDRSINEFIESLGK* |
| *At*UGT78D1  (AT1G30530) | MTKFSEPIRDSHVAVLAFFPVGAHAGPLLAVTRRLAAASPSTIFSFFNTARSNASLFSSDHPENIKVHDVSDGVPEGTMLGNPLEMVELFLEAAPRIFRSEIAAAEIEVGKKVTCMLTDAFFWFAADIAAELNATWVAFWAGGANSLCAHLYTDLIRETIGLKDVSMEETLGFIPGMENYRVKDIPEEVVFEDLDSVFPKALYQMSLALPRASAVFISSFEELEPTLNYNLRSKLKRFLNIAPLTLLSSTSEKEMRDPHGCFAWMGKRSAASVAYISFGTVMEPPPEELVAIAQGLESSKVPFVWSLKEKNMVHLPKGFLDRTREQGIVVPWAPQVELLKHEAMGVNVTHCGWNSVLESVSAGVPMIGRPILADNRLNGRAVEVVWKVGVMMDNGVFTKEGFEKCLNDVFVHDDGKTMKANAKKLKEKLQEDFSMKGSSLENFKILLDEIVKV* |
| *At*UGT73C6  (AT2G36790) | MAFEKNNEPFPLHFVLFPFMAQGHMIPMVDIARLLAQRGVLITIVTTPHNAARFKNVLNRAIESGLPINLVQVKFPYQEAGLQEGQENMDLLTTMEQITSFFKAVNLLKEPVQNLIEEMSPRPSCLISDMCLSYTSEIAKKFKIPKILFHGMGCFCLLCVNVLRKNREILDNLKSDKEYFIVPYFPDRVEFTRPQVPVETYVPAGWKEILEDMVEADKTSYGVIVNSFQELEPAYAKDFKEARSGKAWTIGPVSLCNKVGVDKAERGNKSDIDQDECLEWLDSKEPGSVLYVCLGSICNLPLSQLLELGLGLEESQRPFIWVIRGWEKYKELVEWFSESGFEDRIQDRGLLIKGWSPQMLILSHPSVGGFLTHCGWNSTLEGITAGLPMLTWPLFADQFCNEKLVVQILKVGVSAEVKEVMKWGEEEKIGVLVDKEGVKKAVEELMGESDDAKERRRRAKELGESAHKAVEEGGSSHSNITFLLQDIMQLAQSNN* |
| *At*UGT73C5  (AT2G36800) | MVSETTKSSPLHFVLFPFMAQGHMIPMVDIARLLAQRGVIITIVTTPHNAARFKNVLNRAIESGLPINLVQVKFPYLEAGLQEGQENIDSLDTMERMIPFFKAVNFLEEPVQKLIEEMNPRPSCLISDFCLPYTSKIAKKFNIPKILFHGMGCFCLLCMHVLRKNREILDNLKSDKELFTVPDFPDRVEFTRTQVPVETYVPAGDWKDIFDGMVEANETSYGVIVNSFQELEPAYAKDYKEVRSGKAWTIGPVSLCNKVGADKAERGNKSDIDQDECLKWLDSKKHGSVLYVCLGSICNLPLSQLKELGLGLEESQRPFIWVIRGWEKYKELVEWFSESGFEDRIQDRGLLIKGWSPQMLILSHPSVGGFLTHCGWNSTLEGITAGLPLLTWPLFADQFCNEKLVVEVLKAGVRSGVEQPMKWGEEEKIGVLVDKEGVKKAVEELMGESDDAKERRRRAKELGDSAHKAVEEGGSSHSNISFLLQDIMELAEPNN* |
| *At*UGT74F2  (AT2G43820) | MEHKRGHVLAVPYPTQGHITPFRQFCKRLHFKGLKTTLALTTFVFNSINPDLSGPISIATISDGYDHGGFETADSIDDYLKDFKTSGSKTIADIIQKHQTSDNPITCIVYDAFLPWALDVAREFGLVATPFFTQPCAVNYVYYLSYINNGSLQLPIEELPFLELQDLPSFFSVSGSYPAYFEMVLQQFINFEKADFVLVNSFQELELHENELWSKACPVLTIGPTIPSIYLDQRIKSDTGYDLNLFESKDDSFCINWLDTRPQGSVVYVAFGSMAQLTNVQMEELASAVSNFSFLWVVRSSEEEKLPSGFLETVNKEKSLVLKWSPQLQVLSNKAIGCFLTHCGWNSTMEALTFGVPMVAMPQWTDQPMNAKYIQDVWKAGVRVKTEKESGIAKREEIEFSIKEVMEGERSKEMKKNVKKWRDLAVKSLNEGGSTDTNIDTFVSRVQSK* |
| *At*UGT74F1  (AT2G43840) | MEKMRGHVLAVPFPSQGHITPIRQFCKRLHSKGFKTTHTLTTFIFNTIHLDPSSPISIATISDGYDQGGFSSAGSVPEYLQNFKTFGSKTVADIIRKHQSTDNPITCIVYDSFMPWALDLAMDFGLAAAPFFTQSCAVNYINYLSYINNGSLTLPIKDLPLLELQDLPTFVTPTGSHLAYFEMVLQQFTNFDKADFVLVNSFHDLDLHEEELLSKVCPVLTIGPTVPSMYLDQQIKSDNDYDLNLFDLKEAALCTDWLDKRPEGSVVYIAFGSMAKLSSEQMEEIASAISNFSYLWVVRASEESKLPPGFLETVDKDKSLVLKWSPQLQVLSNKAIGCFMTHCGWNSTMEGLSLGVPMVAMPQWTDQPMNAKYIQDVWKVGVRVKAEKESGICKREEIEFSIKEVMEGEKSKEMKENAGKWRDLAVKSLSEGGSTDININEFVSKIQIK* |
| *At*UGT76B1  (AT3G11340) | METRETKPVIFLFPFPLQGHLNPMFQLANIFFNRGFSITVIHTEFNSPNSSNFPHFTFVSIPDSLSEPESYPDVIEILHDLNSKCVAPFGDCLKKLISEEPTAACVIVDALWYFTHDLTEKFNFPRIVLRTVNLSAFVAFSKFHVLREKGYLSLQETKADSPVPELPYLRMKDLPWFQTEDPRSGDKLQIGVMKSLKSSSGIIFNAIEDLETDQLDEARIEFPVPLFCIGPFHRYVSASSSSLLAHDMTCLSWLDKQATNSVIYASLGSIASIDESEFLEIAWGLRNSNQPFLWVVRPGLIHGKEWIEILPKGFIENLEGRGKIVKWAPQPEVLAHRATGGFLTHCGWNSTLEGICEAIPMICRPSFGDQRVNARYINDVWKIGLHLENKVERLVIENAVRTLMTSSEGEEIRKRIMPMKETVEQCLKLGGSSFRNLENLIAYILSF* |
| *At*UGT84A2  (AT3G21560) | MELESSPPLPPHVMLVSFPGQGHVNPLLRLGKLLASKGLLITFVTTESWGKKMRISNKIQDRVLKPVGKGYLRYDFFDDGLPEDDEASRTNLTILRPHLELVGKREIKNLVKRYKEVTKQPVTCLINNPFVSWVCDVAEDLQIPCAVLWVQSCACLAAYYYYHHNLVDFPTKTEPEIDVQISGMPLLKHDEIPSFIHPSSPHSALREVIIDQIKRLHKTFSIFIDTFNSLEKDIIDHMSTLSLPGVIRPLGPLYKMAKTVAYDVVKVNISEPTDPCMEWLDSQPVSSVVYISFGTVAYLKQEQIDEIAYGVLNADVTFLWVIRQQELGFNKEKHVLPEEVKGKGKIVEWCSQEKVLSHPSVACFVTHCGWNSTMEAVSSGVPTVCFPQWGDQVTDAVYMIDVWKTGVRLSRGEAEERLVPREEVAERLREVTKGEKAIELKKNALKWKEEAEAAVARGGSSDRNLEKFVEKLGAKPVGKVQNGSHNHVLAGSIKSF* |
| *At*UGT72E1  (AT3G50740) | MKITKPHVAMFASPGMGHIIPVIELGKRLAGSHGFDVTIFVLETDAASAQSQFLNSPGCDAALVDIVGLPTPDISGLVDPSAFFGIKLLVMMRETIPTIRSKIEEMQHKPTALIVDLFGLDAIPLGGEFNMLTYIFIASNARFLAVALFFPTLDKDMEEEHIIKKQPMVMPGCEPVRFEDTLETFLDPNSQLYREFVPFGSVFPTCDGIIVNTWDDMEPKTLKSLQDPKLLGRIAGVPVYPIGPLSRPVDPSKTNHPVLDWLNKQPDESVLYISFGSGGSLSAKQLTELAWGLEMSQQRFVWVVRPPVDGSACSAYLSANSGKIRDGTPDYLPEGFVSRTHERGFMVSSWAPQAEILAHQAVGGFLTHCGWNSILESVVGGVPMIAWPLFAEQMMNATLLNEELGVAVRSKKLPSEGVITRAEIEALVRKIMVEEEGAEMRKKIKKLKETAAESLSCDGGVAHESLSRIADESEHLLERVRCMARGA* |
| *At*UGT72B1  (AT4G01070) | MEESKTPHVAIIPSPGMGHLIPLVEFAKRLVHLHGLTVTFVIAGEGPPSKAQRTVLDSLPSSISSVFLPPVDLTDLSSSTRIESRISLTVTRSNPELRKVFDSFVEGGRLPTALVVDLFGTDAFDVAVEFHVPPYIFYPTTANVLSFFLHLPKLDETVSCEFRELTEPLMLPGCVPVAGKDFLDPAQDRKDDAYKWLLHNTKRYKEAEGILVNTFFELEPNAIKALQEPGLDKPPVYPVGPLVNIGKQEAKQTEESECLKWLDNQPLGSVLYVSFGSGGTLTCEQLNELALGLADSEQRFLWVIRSPSGIANSSYFDSHSQTDPLTFLPPGFLERTKKRVRAKWQPLNI* |
| *At*UGT84A1  (AT4G15480) | MGSISEMVFETCPSPNPIHVMLVSFQGQGHVNPLLRLGKLIASKGLLVTFVTTELWGKKMRQANKIVDGELKPVGSGSIRFEFFDEEWAEDDDRRADFSLYIAHLESVGIREVSKLVRRYEEANEPVSCLINNPFIPWVCHVAEEFNIPCAVLWVQSCACFSAYYHYQDGSVSFPTETEPELDVKLPCVPVLKNDEIPSFLHPSSRFTGFRQAILGQFKNLSKSFCVLIDSFDSLEQEVIDYMSSLCPVKTVGPLFKVARTVTSDVSGDICKSTDKCLEWLDSRPKSSVVYISFGTVAYLKQEQIEEIAHGVLKSGLSFLWVIRPPPHDLKVETHVLPQELKESSAKGKGMIVDWCPQEQVLSHPSVACFVTHCGWNSTMESLSSGVPVVCCPQWGDQVTDAVYLIDVFKTGVRLGRGATEERVVPREEVAEKLLEATVGEKAEELRKNALKWKAEAEAAVAPGGSSDKNFREFVEKLGAGVTKTKDNGY* |
| *At*UGT84A3  (AT4G15490) | MDPSRHTHVMLVSFPGQGHVNPLLRLGKLIASKGLLVTFVTTEKPWGKKMRQANKIQDGVLKPVGLGFIRFEFFSDGFADDDEKRFDFDAFRPHLEAVGKQEIKNLVKRYNKEPVTCLINNAFVPWVCDVAEELHIPSAVLWVQSCACLTAYYYYHHRLVKFPTKTEPDISVEIPCLPLLKHDEIPSFLHPSSPYTAFGDIILDQLKRFENHKSFYLFIDTFRELEKDIMDHMSQLCPQAIISPVGPLFKMAQTLSSDVKGDISEPASDCMEWLDSREPSSVVYISFGTIANLKQEQMEEIAHGVLSSGLSVLWVVRPPMEGTFVEPHVLPRELEEKGKIVEWCPQERVLAHPAIACFLSHCGWNSTMEALTAGVPVVCFPQWGDQVTDAVYLADVFKTGVRLGRGAAEEMIVSREVVAEKLLEATVGEKAVELRENARRWKAEAEAAVADGGSSDMNFKEFVDKLVTKHVTREDNGEH* |
| *At*UGT84A4  (AT4G15500) | MEMESSLPHVMLVSFPGQGHISPLLRLGKIIASKGLIVTFVTTEEPLGKKMRQANNIQDGVLKPVGLGFLRFEFFEDGFVYKEDFDLLQKSLEVSGKREIKNLVKKYEKQPVRCLINNAFVPWVCDIAEELQIPSAVLWVQSCACLAAYYYYHHQLVKFPTETEPEITVDVPFKPLTLKHDEIPSFLHPSSPLSSIGGTILEQIKRLHKPFSVLIETFQELEKDTIDHMSQLCPQVNFNPIGPLFTMAKTIRSDIKGDISKPDSDCIEWLDSREPSSVVYISFGTLAFLKQNQIDEIAHGILNSGLSCLWVLRPPLEGLAIEPHVLPLELEEKGKIVEWCQQEKVLAHPAVACFLSHCGWNSTMEALTSGVPVICFPQWGDQVTNAVYMIDVFKTGLRLSRGASDERIVPREEVAERLLEATVGEKAVELRENARRWKEEAESAVAYGGTSERNFQEFVDKLVDVKTMTNINNVV* |
| *At*UGT73B2  (AT4G34135) | MGSDHHHRKLHVMFFPFMAYGHMIPTLDMAKLFSSRGAKSTILTTSLNSKILQKPIDTFKNLNPGLEIDIQIFNFPCVELGLPEGCENVDFFTSNNNDDKNEMIVKFFFSTRFFKDQLEKLLGTTRPDCLIADMFFPWATEAAGKFNVPRLVFHGTGYFSLCAGYCIGVHKPQKRVASSSEPFVIPELPGNIVITEEQIIDGDGESDMGKFMTEVRESEVKSSGVVLNSFYELEHDYADFYKSCVQKRAWHIGPLSVYNRGFEEKAERGKKANIDEAECLKWLDSKKPNSVIYVSFGSVAFFKNEQLFEIAAGLEASGTSFIWVVRKTKGIEIDV* |
| *At*UGT89A2  (AT5G03490) | MTEVLLLPGTKSENSKPPHIVVFPFPAQGHLLPLLDLTHQLCLRGFNVSVIVTPGNLTYLSPLLSAHPSSVTSVVFPFPPHPSLSPGVENVKDVGNSGNLPIMASLRQLREPIINWFQSHPNPPIALISDFFLGWTHDLCNQIGIPRFAFFSISFFLVSVLQFCFENIDLIKSTDPIHLLDLPRAPIFKEEHLPSIVRRSLQTPSPDLESIKDFSMNLLSYGSVFNSSEILEDDYLQYVKQRMGHDRVYVIGPLCSIGSGLKSNSGSVDPSLLSWLDGSPNGSVLYVCFGSQKALTKDQCDALALGLEKSMTRFVWVVKKDPIPDGFEDRVSGRGLVVRGWVSQLAVLRHVAVGGFLSHCGWNSVLEGITSGAVILGWPMEADQFVNARLLVEHLGVAVRVCEGGETVPDSDELGRVIAETMGEGGREVAARAEEIRRKTEAAVTEANGSSVENVQRLVKEFEKV* |
| *At*UGT76C2  (AT5G05860) | MEEKRNGLRVILFPLPLQGCINPMLQLANILHVRGFSITVIHTRFNAPKASSHPLFTFLQIPDGLSETEIQDGVMSLLAQINLNAESPFRDCLRKVLLESKESERVTCLIDDCGWLFTQSVSESLKLPRLVLCTFKATFFNAYPSLPLIRTKGYLPVSESEAEDSVPEFPPLQKRDLSKVFGEFGEKLDPFLHAVVETTIRSSGLIYMSCEELEKDSLTLSNEIFKVPVFAIGPFHSYFSASSSSLFTQDETCILWLDDQEDKSVIYVSLGSVVNITETEFLEIACGLSNSKQPFLWVVRPGSVLGAKWIEPLSEGLVSSLEEKGKIVKWAPQQEVLAHRATGGFLTHNGWNSTLESICEGVPMICLPGGWDQMLNSRFVSDIWKIGIHLEGRIEKKEIEKAVRVLMEESEGNKIRERMKVLKDEVEKSVKQGGSSFQSIETLANHILLL* |
| *At*UGT78D2  (AT5G17050) | MTKPSDPTRDSHVAVLAFPFGTHAAPLLTVTRRLASASPSTVFSFFNTAQSNSSLFSSGDEADRPANIRVYDIADGVPEGYVFSGRPQEAIELFLQAAPENFRREIAKAETEVGTEVKCLMTDAFFWFAADMATEINASWIAFWTAGANSLSAHLYTDLIRETIGVKEVGERMEETIGVISGMEKIRVKDTPEGVVFGNLDSVFSKMLHQMGLALPRATAVFINSFEDLDPTLTNNLRSRFKRYLNIGPLGLLSSTLQQLVQDPHGCLAWMEKRSSGSVAYISFGTVMTPPPGELAAIAEGLESSKVPFVWSLKEKSLVQLPKGFLDRTREQGIVVPWAPQVELLKHEATGVFVTHCGWNSVLESVSGGVPMICRPFFGDQRLNGRAVEVVWEIGMTIINGVFTKDGFEKCLDKVLVQDDGKKMKCNAKKLKELAYEAVSSKGRSSENFRGLLDAVVNII* |
| *At*UGT72E3  (AT5G26310) | MHITKPHAAMFSSPGMGHVLPVIELAKRLSANHGFHVTVFVLETDAASVQSKLLNSTGVDIVNLPSPDISGLVDPNAHVVTKIGVIMREAVPTLRSKIVAMHQNPTALIIDLFGTDALCLAAELNMLTYVFIASNARYLGVSIYYPTLDEVIKEEHTVQRKPLTIPGCEPVRFEDIMDAYLVPDEPVYHDLVRHCLAYPKADGILVNTWEEMEPKSLKSLQDPKLLGRVARVPVYPVGPLCRPIQSSTTDHPVFDWLNKQPNESVLYISFGSGGSLTAQQLTELAWGLEESQQRFIWVVRPPVDGSSCSDYFSAKGGVTKDNTPEYLPEGFVTRTCDRGFMIPSWAPQAEILAHQAVGGFLTHCGWSSTLESVLCGVPMIAWPLFAEQNMNAALLSDELGISVRVDDPKEAISRSKIEAMVRKVMAEDEGEEMRRKVKKLRDTAEMSLSIHGGGSAHESLCRVTKECQRFLECVGDLGRGA* |
| *At*UGT79B6  (AT5G54010) | MGSKFHAFMFPWFGFGHMTAFLHLANKLAEKDHKITFLLPKKARKQLESLNLFPDCIVFQTLTIPSVDGLPDGAETTSDIPISLGSFLASAMDRTRIQVKEAVSVGKPDLIFFDFAHWIPEIAREYGVKSVNFITISAACVAISFVPGRSQDDLGSTPPGYPSSKVLLRGHETNSLSFLSYPFGDGTSFYERIMIGLKNCDVISIRTCQEMEGKFCDFIENQFQRKVLLTGPMLPEPDNSKPLEDQWRQWLSKFDPGSVIYCALGSQIILEKDQFQELCLGMELTGLPFLVAVKPPKGSSTIQEALPKGFEERVKARGVVWGGWVQQPLILAHPSIGCFVSHCGFGSMWEALVNDCQIVFIPHLGEQILNTRLMSEELKVSVEVKREETGWFSKESLSGAVRSVMDRDSELGNWARRNHVKWKESLLRHGLMSGYLNKFVEALEKLVQNINLE* |
| *At*UGT79B1  (AT5G54060) | MGVFGSNESSSMSIVMYPWLAFGHMTPFLHLSNKLAEKGHKIVFLLPKKALNQLEPLNLYPNLITFHTISIPQVKGLPPGAETNSDVPFFLTHLLAVAMDQTRPEVETIFRTIKPDLVFYDSAHWIPEIAKPIGAKTVCFNIVSAASIALSLVPSAEREVIDGKEMSGEELAKTPLGYPSSKVVLRPHEAKSLSFVWRKHEAIGSFFDGKVTAMRNCDAIAIRTCRETEGKFCDYISRQYSKPVYLTGPVLPGSQPNQPSLDPQWAEWLAKFNHGSVVFCAFGSQPVVNKIDQFQELCLGLESTGFPFLVAIKPPSGVSTVEEALPEGFKERVQGRGVVFGGWIQQPLVLNHPSVGCFVSHCGFGSMWESLMSDCQIVLVPQHGEQILNARLMTEEMEVAVEVEREKKGWFSRQSLENAVKSVMEEGSEIGEKVRKNHDKWRCVLTDSGFSDGYIDKFEQNLIELVKS* |
| *At*UGT72E2  (AT5G66690) | MHITKPHAAMFSSPGMGHVIPVIELGKRLSANNGFHVTVFVLETDAASAQSKFLNSTGVDIVKLPSPDIYGLVDPDDHVVTKIGVIMRAAVPALRSKIAAMHQKPTALIVDLFGTDALCLAKEFNMLSYVFIPTNARFLGVSIYYPNLDKDIKEEHTVQRNPLAIPGCEPVRFEDTLDAYLVPDEPVYRDFVRHGLAYPKADGILVNTWEEMEPKSLKSLLNPKLLGRVARVPVYPIGPLCRPIQSSETDHPVLDWLNEQPNESVLYISFGSGGCLSAKQLTELAWGLEQSQQRFVWVVRPPVDGSCCSEYVSANGGGTEDNTPEYLPEGFVSRTSDRGFVVPSWAPQAEILSHRAVGGFLTHCGWSSTLESVVGGVPMIAWPLFAEQNMNAALLSDELGIAVRLDDPKEDISRWKIEALVRKVMTEKEGEAMRRKVKKLRDSAEMSLSIDGGGLAHESLCRVTKECQRFLERVVDLSRGA* |
| *At*UGT76C4  (AT5G05880) | MEKSNGLRVILFPLPLQGCINPMIQLAKILHSRGFSITVIHTCFNAPKASSHPLFTFIQIQDGLSETETRTRDVKLLITLLNQNCESPVRECLRKLLQSAKEEKQRISCLINDSGWIFTQHLAKSLNLMRLAFNTYKISFFRSHFVLPQLRREMFLPLQDSEQDDPVEKFPPLRKKDLLRILEADSVQGDSYSDMILEKTKASSGLIFMSCEELDQDSLSQSREDFKVPIFAIGPSHSHFPASSSSLFTPDETCIPWLDRQEDKSVIYVSIGSLVTINETELMEIAWGLSNSDQPFLWVVRVGSVNGTEWIEAIPEYFIKRLNEKGKIVKWAPQQEVLKHRAIGGFLTHNGWNSTVESVCEGVPMICLPFRWDQLLNARFVSDVWMVGIHLEGRIERDEIERAIRRLLLETEGEAIRERIQLLKEKVGRSVKQNGSAYQSLQNLINYISSF* |
| *At*UGT71B6  (AT3G21780) | MITSLTSNNRLRYEIISGGDQQPTELKATDSHIQSLKPLVRDAVAKLVDSTLPDAPRLAGFVVDMYCTSMIDVANEFGVPSYLFYTSNAGFLGLLLHIQFMYDAEDIYDMSELEDSDVELVVPSLTSPYPLKCLPYIFKSKEWLTFFVTQARRFRETKGILVNTVPDLEPQALTFLSNGNIPRAYPVGPLLHLKNVNCDYVDKKQSEILRWLDEQPPRSVVFLCFGSMGGFSEEQVRETALALDRSGHRFLWSLRRASPNILREPPGEFTNLEEILPEGFFDRTANRGKVIGWAEQVAILAKPAIGGFVSHGGWNSTLESLWFGVPMAIWPLYAEQKFNAFEMVEELGLAVEIKKHWRGDLLLGRSEIVTAEEIEKGIICLMEQDSDVRKRVNEISEKCHVALMDGGSSETALKRFIQDVTENIAWSETES* |
| *Pl*UGT88E20  (KU311040) | MKDTIVLYPNIGRGHLVSMVELGKLILTHHPSLSITILILTPSTTPSTTTFACDSNAQYIATVTATIPAITFHHVPLATLPSNTPSLPPHLVSLELARHSTQNVAVAFQTLAKASNLKAIIIDLLNFNDPKTLTQNLNKNIHTYFYYTSGASTLALLLHYPTIHETLTKNYVKDQPLQIQIPGLRANITTDDFAKDSKDPSNYSSQAFLKIAETMRGSFGIIINTFEAIEEELIRALSEDGTVPPLFCIGPVISAPYGEDDKGCLSWLDSQPSQSVVLLCFGSMGSFSRTQLKEIAVGLEKSEQRFLWVVRAELDCADSVDEQPSLDELMPGGFLERTKEKGLVVRDWAPQVQILSHDSVGGFVTHCGWNSVLEAVCEGVPMAAWPLYAEQRVNRVIMVEDMKVALAVNEDKAGFVSATELGDRVRELMESDKGKEIRQRTFKMKISAAEAMAEGGTSRVALDKLAKLWKES* |
| *Pg*UGT84A24  (KT159807) | MGSESLVHVFLVSFPGQGHVNPLLRLGKRLASKGLLVTFTTPESIGKQMRKASNIGEEPSPIGDGFIRFEFFEDGWDEDEPRRQDLDQYLPQLEKVGKEVIPRMIKKNEEQNRPVSCLINNPFIPWVSDVAESLGLPSAMLWVQSCACFAAYYHYYHGLVPFPSESAMEIDVQLPCMPLLKHDEVPSFLYPTTPYPFLRRAIMGQYKNLDKPFCVLMDTFQELEHEIIEYMSKICPIKTVGPLFKNPKAPNANVRGDFMKADDCISWLDSKPPASVVYVSFGSVVYLKQDQWDEIAFGLLNSGLNFLWVMKPPHKDSGYQLLTLPEGFLEKAGDKGKVVQWSPQEQVLAHPSVACFVTHCGWNSSMEALSSGMPVVAFPQWGDQVTDAKYLVDVFKVGVRMCRGEAENKLIMRDVVEKCLLEATVGPKAAEVKENALKWKAAAEAAVAEGGSSDRNIQAFVDEVKRRSIAIQSNKSEPKPVVQNAAVADHFGAKATTNGVAADLAGSNADGKVELVA* |
| *Fk*UGT71A18  (AB524718) | MAETKKSELVFIPAPGIGHLISTIELAKLLTDRDEHLSITVLILKLPMESKTDSYSQKSNSRIRFIELSLNQPITPNNFVTDFIEGHKDPIRDAVTKIVRDESNSIRLAGFVIDMFCTTMIDVANEFGVPTYVFFTTTAAMLGFIFYLQSRGDEQKLDVTEYKNSNTKLLIPTYINPVPANVFPSKLFDKDSLAPFVSMARRFRETKGILINTFLDLEAYALKSLSDDHTIPPVYSIGPILHVKVENDDKKKDYDEIINWLHEQPVSSVVFLCFGSLGCFDVEQVKEIAVALEKSGHRFLWSLRKPPPKDFEHPSDYENFEEVLPEGFLQRTAGIGKVIGWAPQVAVLSHHSVGGFVSHCGWNSTLESVWCGVPIAAWPMYAEQQTNAFELVKDLGIAVEIKMDYRKGSDVIVKAEEIEKGIKHLMEPDSEMRNKMKQMKSKSRLALMEGGSSYNFLRRFIDNIPMTD* |
| *Lu*UGT74S1  (JX011632) | mtvtaqsgihidtisdgfdhsglilqdpehysqtfrrvgsetltdlirkqsesrhpvhciiydasmpwfldvakrfgivgaafltqscavnaiyyhlregtikrpvvsdpaagtlvidglpplevsdlpsfiwddlhteflaahlrqfsndgadwvfcntvyqleleavdwltkqwlinfrtigptipsfyldkqipddkdydisifnpqnqtcmnwlqskpdgsvvyvsfgslarlspqqteelyfglknsnhyflwvvresevaklpkeeylsgekglvvswcsqlqvlasgkvgcfvthcgwnstlealslgvpmvampecgdqltnakfikdvwktgvraeaddgkgimwgmikreviercirevmegeetrrnadkwgkiikeavveggssdkntedfatslinfaetfqfsc* |
| *Si*UGT71A9  (AB293960) | MSADQKLTSLVFVPFPIMSHLATAVKTAKLLADRDERLSITVLVMKLPIDTLISSYTKNSPDARVKVVQLPEDEPTFTKLMKSSKNFFFRYIESQKGTVRDAVAEIMKSSRSCRLAGFVIDMFCTTMIDVANELGVPTYMFFSSGSATLGLMFHLQSLRDDNNVDVMEYKNSDAAISIPTYVNPVPVAVWPSPVFEEDSGFLDFAKRFRETKGIIVNTFLEFETHQIRSLSDDKKIPPVYPVGPILQADENKIEQEKEKHAEIMRWLDKQPDSSVVFLCFGTHGCLEGDQVKEIAVALENSGHRFLWSLRKPPPKEKVEFPGEYENSEEVLPEGFLGRTTDMGKVIGWAPQMAVLSHPAVGGFVSHCGWNSVLESVWCGVPMAVWPLSAEQQANAFLLVKEFEMAVEIKMDYKKNANVIVGTETIEEAIRQLMDPENEIRVKVRALKEKSRMALMEGGSSYNYLKRFVENVVNNIS* |
| *Si*UGT94D1  (AB333799) | MDTRKRSIRILMFPWLAHGHISAFLELAKSLAKRNFVIYICSSQVNLNSISKNMSSKDSISVKLVELHIPTTILPPPYHTTNGLPPHLMSTLKRALDSARPAFSTLLQTLKPDLVLYDFLQSWASEEAESQNIPAMVFLSTGAAAISFIMYHWFETRPEEYPFPAIYFREHEYDNFCRFKSSDSGTSDQLRVSDCVKRSHDLVLIKTFRELEGQYVDFLSDLTRKRFVPVGPLVQEVGCDMENEGNDIIEWLDGKDRRSTVFSSFGSEYFLSANEIEEIAYGLELSGLNFIWVVRFPHGDEKIKIEEKLPEGFLERVEGRGLVVEGWAQQRRILSHPSVGGFLSHCGWSSVMEGVYSGVPIIAVPMHLDQPFNARLVEAVGFGEEVVRSRQGNLDRGEVARVVKKLVMGKSGEGLRRRVEELSEKMREKGEEEIDSLVEELVTVVRRRERSNLKSENSMKKLNVMDDGE* |
| *Bn*UGT71B5  (XP_013672739) | MTMELVFIPSPGIGHLRSTVELAKQLVNGDERLSITVIIIPRSSGGDASDFAQISSFFTPSQDRLRHETISVADNPTGERLPTQVYIANQKPQVRDAVAKLLDPTGVNSPSPPRLAGFVIDMFCISMMDVADEFGVPTYMVYTSNAAFLGFSLHLQKMYDEKKLDTSELSESVNELEVPCFSRPYPVGCLPYIFVSKEWLPLFLAQARNFRKMKGILVNTVAELEPQALNVLSRDDGGDLPRAYPIGPVLHLQNGSRHDDGGKESEILRWLDEQPAKSVVFLCFGSLGGFSDEQTREIAVALDRSGHRFLWSLRRASPNILTEGPGDYTNLEEVLPEGFLDRTSDRGKVIGWAPQVAVLAKPAIGGFVTHCGWNSMLESLWFGVPMVTWPLYAEQKVNAFEMVEELGLAVEIRRFIKGDLLEGVMETVTAEDLERAITRVMEEDSDVRNKVNEVAEKCHVALMDGGSSKTALRKFIQDVVENVVV* |
| Notes: These sequence data of *Ii*UGTs have been submitted to the GenBank databases under accession number: PRJNA668701. The informations of *At*UGTs retrieved from http://www.p450.kvl.dk/UGT.shtml. *Ii*, *Isatis indigotica* Fort.; *Fk*, *Forsythia koreana*; *Lu*, *Linum usitatissimum* L.; *Si*, *Sesamum indicum* Linn.; *Bn*, *Brassica napus*; *Pg*, *Punica granatum*; *Pl*, *Pueraria lobata*; *At*, *Arabidopsis thaliana*. | |

| **Supplementary Table 5.** Abundance of lignans in different tissues of *Isatis indigotica* | | | | | |
| --- | --- | --- | --- | --- | --- |
| **Abundance (μg/g)** | **Leaf** | **Root** | **Epidermis and Cortex** | **Phloem** | **Xylem and Cambium** |
| Pinoresinol | -- | -- | -- | -- | -- |
| (+)-Pinoresinol-4-*O*-glucoside | 0.55±0.09 | 4.30±3.46 | 2.56±1.75 | 0.76±0.42 | 0.20±0.11 |
| Pinoresinol diglucoside | 0.06±0.10 | 82.16±56.39 | 57.28±68.91 | 14.89±6.81 | 4.45±2.97 |
| Lariciresinol | 5.98±3.08 | 2.24±1.15 | -- | -- | 2.43±0.64 |
| Secoisolariciresinol | 2.87±1.87 | -- | -- | -- | -- |
| Secoisolariciresinol monoglucoside | 1.59±1.20 | -- | -- | -- | -- |
| Secoisolariciresinol diglucoside | -- | -- | -- | -- | -- |
| Matairesinol | -- | -- | -- | -- | -- |
| Matairesinol monoglucoside | -- | -- | -- | -- | -- |
| Isolariciresinol | -- | -- | -- | -- | -- |
| (-)-Isolariciresinol-9′-*O*-glucoside | -- | -- | -- | -- | -- |
| Phillygenin | -- | -- | -- | -- | -- |
| Forsythin | -- | -- | -- | -- | -- |
| Sesaminol | -- | -- | -- | -- | -- |
| Coniferyl alcohol | -- | -- | -- | -- | -- |
| trans-Coniferin | -- | 3200.50±303.84 | 3427.04±608.65 | 3754.86±253.32 | 2156.60±564.52 |
| Note: "--" mean not detected, n=3. | | | | | |

| **Supplementary Table 6.** The reaction time of recombined UGTs to different substrates | | | | |
| --- | --- | --- | --- | --- |
| **UGT** | **Sugar receptor** | **Reaction temperature（℃）** | **Rotating speed（rpm）** | **Reaction time (min)** |
| *Ii*UGT71B5a | Pinoresinol | 30 | 300 | 5 |
|  | (+)-Pinoresinol-4-*O*-glucoside | 30 | 300 | 20 |
|  | Clemaphenol A | 30 | 300 | 10 |
|  | Sesaminol | 30 | 300 | 5 |
|  | Matairesinol | 30 | 300 | 5 |
|  | Isolariciresinol | 30 | 300 | 5 |
|  | Secoisolariciresinol | 30 | 300 | 5 |
|  | Lariciresinol | 30 | 300 | 40 |
|  | Phillygenin | 30 | 300 | 60 |
| *Ii*UGT71B5b | Sesaminol | 30 | 300 | 40 |
|  | Secoisolariciresinol | 30 | 300 | 40 |
|  | Pinoresinol | 30 | 300 | 40 |
|  | Phillygenin | 30 | 300 | 60 |
